# Interfacial Force‐Focusing Effect in Mechanophore‐Linked Nanocomposites

**DOI:** 10.1002/advs.201903464

**Published:** 2020-02-26

**Authors:** Tae Ann Kim, Caterina Lamuta, Hojun Kim, Cecilia Leal, Nancy R. Sottos

**Affiliations:** ^1^ Department of Materials Science and Engineering University of Illinois at Urbana‐Champaign Urbana IL 61801 USA; ^2^ Photo‐Electronic Hybrids Research Center Korea Institute of Science and Technology (KIST) Seoul 02792 Republic of Korea; ^3^ Beckman Institute for Advanced Science and Technology University of Illinois at Urbana‐Champaign Urbana IL 61801 USA; ^4^ Department of Mechanical Engineering University of Iowa Iowa City IA 52242 USA; ^5^ Department of Materials Science and Engineering Beckman Institute for Advanced Science and Technology University of Illinois at Urbana‐Champaign Urbana IL 61801 USA

**Keywords:** force focusing, interfacial effects, mechanophores, nanocomposites

## Abstract

Enhanced force transmission to mechanophores is demonstrated in polymer nanocomposite materials. Spiropyran (SP) mechanophores that change color and fluorescence under mechanical stimuli are functionalized at the interface between SiO_2_ nanoparticles and polymers. Successful mechanical activation of SP at the interface is confirmed in both solution and solid states. Compared with SP‐linked in bulk polymers, interfacial activation induces greater conversion of SP to its colored merocyanine form and also significantly decreases the activation threshold under tension. Experimental observations are supported by finite element simulation of the interfacial stress state. The interfacial force‐focusing strategy opens a new way to control the reactivity of mechanophores and also potentially indicates interfacial damage in composite materials.

## Graphical abstract

Functionalized nanoparticle interfaces enhance mechanochemical activity in solid polymers. The particles create a force‐focusing effect, causing significantly more mechanophores to activate at lower threshold strains compared to mechanophores linked directly into the polymer chain.

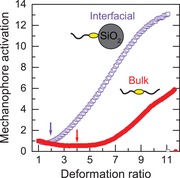

Mechanophore‐linked polymers translate destructive forces into productive chemical reactions including changes in optical properties,^[^ 1, 2, 3, 4 ^]^ small molecule or catalyst release,^[^ 5, 6, 7 ^]^ and remodeling.^[^ 8, 9 ^]^ To fully utilize these beneficial mechanochemical reactions, we need to better understand how the macroscopic force is transferred to the molecular level in a polymer.^[^ 10, 11 ^]^ In a dilute polymer solution under extensional flow field, the force is maximum at the center of the polymer chain, and can enhance mechanophore activation.^[^ 12, 13, 14 ^]^ In a bulk polymer, force activation is more complicated due to the inhomogeneous force distribution controlled by the polymer chain morphology, that is, chain entanglement and configuration.^[^ 15 ^]^ Mechanophores have been conjugated in a polymer backbone^[^ 16 ^]^ or incorporated as cross‐linkers,^[^ 17 ^]^ but only small fraction of mechanophores in the polymer are mechanically activated. Researchers have identified several possible methods to enhance mechanophore activation in a bulk polymer including alignment of polymer chains along the direction of a macroscopic force^[^ 18, 19 ^]^ and design of multi‐armed polymer architectures.^[^ 20 ^]^

Polymer composites provide an interesting platform to demonstrate force‐focusing strategies for achieving enhanced force transmission to mechanophores. In composites, external forces are transferred through the interfaces between filler and matrix,^[^ 21, 22 ^]^ suggesting mechanophores located at the interfaces will experience maximum force transmission. Kosuge et al. reported indirect evidence that diarylbibenzofuranone (DABBF) mechanophores at organic/inorganic interfaces are preferentially activated.^[^ 23 ^]^ They hypothesized that mobility of the chain attached to the DABBF results in different activation response as well as changes in recombination kinetics of the mechanically cleaved DABBF species. However, the mechanophore‐functionalized interface in this study was not well defined and the activation of the interfacial mechanophores was not compared to the mechanophores linked in bulk polymers. Recently, Park et al. reported a hierarchical nanoparticle‐in‐micropore architecture that enhanced the mechanochemical activation of mechanophores in polymeric composites.^[^ 24 ^]^ The introduction of pores and nanoparticles to bulk polymers created a stress concentration effect that increased the mechanochemical sensitivity of the composites.

Here, we demonstrate the mechanical activation of spiropyran (SP) mechanophores at the interface between poly(methyl acrylate) (PMA) and SiO_2_ nanoparticles both in solution and solid states (**Figure** **1**a). SP mechanophores, which undergo 6‐π electrocyclic ring‐opening reaction under UV or mechanical stimuli, are functionalized on the surface of SiO_2_ particles. Then, linear or cross‐linked PMA (xPMA) is synthesized from the opposing side of the spiro junction that enables efficient force transmission across the spiro C–O bond. Three SP mechanophores (**1**, **2**, and **3** in Figure 1b) and three SP controls (**4**, **5**, and **6** in Figure 1b) are designed and synthesized. Control samples are prepared to confirm the observed color or fluorescence change originated from mechanical force and not from other factors (**A‐Int** vs **C‐I** vs **C‐II** in Figure 1c). Finally, we compare the reactivity of SP mechanophores functionalized at the interface between the SiO_2_ particles and the polymer matrix (**A‐Int** in Figure 1c) with that of SPs covalently linked to a bulk polymer (**A‐Bulk** in Figure 1c).

**Figure 1:**
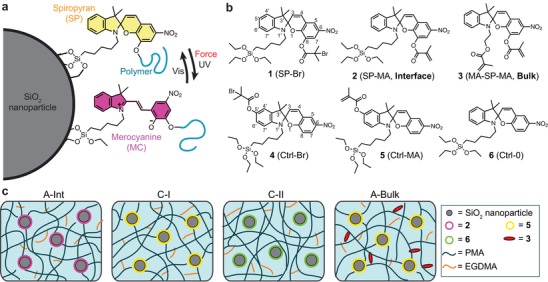
Mechanophore‐linked polymer nanocomposites. a) Schematic of SP‐linked nanoparticle. Under force or UV exposure, SP located at the particle interface is activated and changes color. b) Chemical structures of three mechanically active SPs (**1**, **2**, and **3**) and three control SPs (**4**, **5**, and **6**). **4** and **5** form covalent bonds with both SiO_2_ and polymer chains but the force cannot be transferred across spiro (C–O) junction. **6** is able to bond to SiO_2_ but not to polymers. c) Schematics of different specimen types: **A‐Int** is the active interfacial specimen in which **2** is functionalized on the SiO_2_ surfaces and also forms a cross‐linking network with the PMA chains. External force should trigger the ring‐opening reaction of the interfacial SPs. **C‐I** is a control specimen in which **5** is covalently linked with SiO_2_ and PMA, but the transferred force cannot break spiro junction that causes the change in color and fluorescence. **C‐II** is a control specimen with **6** covalently linked only with SiO_2_ so that no force transmission occurs to **6**. **A‐Bulk** is an active bulk polymer specimen in which **3** acts as additional cross‐linker for PMA chains. Mechanically active SPs are only located in bulk PMA matrix. Additional cross‐linker, EGDMA is added to control the overall cross‐linking density.

SP molecules were immobilized to SiO_2_ particles using the silylation reaction between hydrolyzed triethoxysilane groups and the silanol from the surfaces of SiO_2_.^[^ 25 ^]^ The silylation reaction was performed in a nonpolar solvent (toluene) with organic base (triethylamine). The conventional method to increase the functionalized surface coverage uses ammonia as a catalyst;^[^ 26, 27 ^]^ however, MC isomers of SP with nitro functionality are easily hydrolyzed by this process, resulting in loss of photochromic behavior.^[^ 28 ^]^ This hydrolytic decomposition is accelerated at higher pH conditions. Indeed, when ammonia was added to the SP‐dissolved solution, the solution did not change its color after UV exposure, indicating the destruction of the SP chemical structure (Figure S1, Supporting Information). Alternatively, triethylamine can promote the silylation reaction of SiO_2_ with alkoxysilanes.^[^ 29 ^]^ We also confirmed that the addition of triethylamine did not trigger the degradation of SP in organic solvents.

Both micron‐sized particles and nanoparticles were successfully functionalized with this improved method. Micron‐sized particles were used exclusively to facilitate fluorescence imaging. Investigations of mechanochemical response focused on nanoparticles. Optical and fluorescence images of bare SiO_2_ and SP‐functionalized SiO_2_ (SiO_2_@SP) particles were observed after UV exposure (Figure S2, Supporting Information). Only SiO_2_@SP particles exhibited strong red fluorescence signals unlike bare SiO_2_.

For SiO_2_ particles functionalized with **1** or **4**, linear PMA was grown at the particle surface using a single‐electron transfer living radical polymerization method. Particles with PMA exhibited a distinct change in the dried morphology compared with bare SiO_2_ (Figure S3, Supporting Information). Successful surface‐initiated polymerization from SiO_2_ nanoparticles was also confirmed by transmission electron microscopy (TEM) (Figure S4, Supporting Information). The grafting density of the PMA was calculated from the similar method described by Li et al.,^[^ 30 ^]^ which was 0.01–0.04 chains nm^–2^ (Figure S5, Supporting Information). We also found that attached PMA on the SiO_2_ has a higher glass transition temperature than free PMA with the same molecular weight (Figure S6, Supporting Information).

We first tested the ability to mechanically activate our functionalized nanoparticles in solution using a well‐established sonication technique.^[^ 13 ^]^ For sonication, polymer chains with higher molecular weight result in improved activation.^[^ 5, 31 ^]^ In this work, we conducted sonication experiments only on our highest molecular weights of PMA‐functionalized particles (277 kg mol^−1^, grafting density = 0.01 chains nm^–2^). Both active and control particle dispersed solutions (2.5 mg mL^–1^ in methyl ether ketone) were prepared and subjected to pulsed ultrasound (Figure S7, Supporting Information). Only active particles changed to a visible pink color after ultrasonication, indicating successful mechanochemical ring opening of the SP mechanophores. UV–vis spectra also confirmed that a new absorbance band centered at around 540 nm increased with the sonication time. Although control particles are mechanically inactive, photochromic behavior is maintained.

The mechanical activation of the particles in a host polymer was demonstrated by blending the SP‐functionalized particles and pristine PMA (*M*_n_ = 271 kg mol^−1^, *Đ* = 1.25) to make composites. We hypothesized that the chain entanglement between the PMA matrix and the surface‐initiated PMA on the particles can transfer force to SP at the interface, leading to color change only for the mechanically active SP. Composites were prepared with 16 wt% (10 vol%) of active (**1**) or control (**4**) nanoparticles grafted with PMA in the linear PMA matrix. The materials were molded into tensile specimens. Additional control specimens were also fabricated with SiO_2_ particles containing **6**, which had no surface grafted PMA, therefore no force transfer occurred to SP. All the composites changed color under UV light, confirming the presence of active SP molecules (Figure S8, Supporting Information). However, in contrast to bifunctional (**4**) or monofunctional (**6**) control SPs, only active (**1**) samples exhibited color change in the gauge area after stretching (Figure S8 and S9, Supporting Information). Because of the low grafting density of SP‐PMA (0.01 chains nm^–2^), intense color and fluorescence change were not observed from this system.

To increase the amount of SP available for mechanical activation, we investigated SiO_2_ nanoparticles functionalized with cross‐linkable SP (**2** and **5**). An additional cross‐linking agent, ethylene glycol dimethacrylate (EGDMA), was added to control overall cross‐linking density of PMA. As expected, only xPMA containing active SP particles (**A‐Int**) changed color after mechanical loading (**Figure** **2**a; Figure S10, Supporting Information). As expected, both **C‐I** and **C‐II** control samples showed no color change under applied tension. In addition, the active xPMA specimens generated more intense color than the linear PMA composites with active SP particles. The xPMA specimens with control SP only exhibited photochromic behavior.

**Figure 2:**
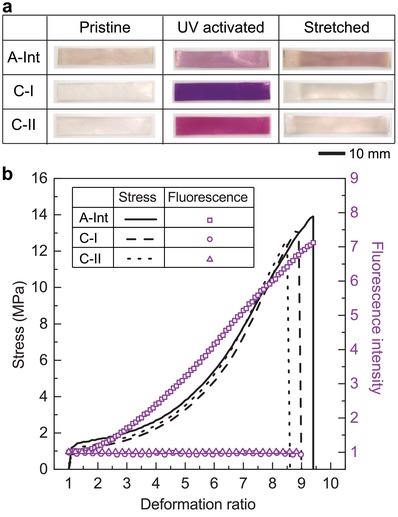
Cross‐linked PMA with SP‐linked SiO_2_ nanoparticles. a) Optical images of cross‐linked PMA specimens (strips) incorporating SP‐functionalized SiO_2_ (1.5 wt%) and 0.8 mol% of EGDMA. Only the **A‐Int** sample changes color under applied tensile loading. b) Combined stress and fluorescence intensity data as a function of deformation ratio for **A‐Int**, **C‐I**, and **C‐II**. Change in fluorescence intensity is only observed from the **A‐Int** sample. Also, the **A‐Int** sample exhibits enhanced mechanical properties compared to other control samples.

The intensity of fluorescence signal was simultaneously measured with tensile specimens under uniaxial load to monitor the activation of the SP mechanophores (Figure 2b).^[^ 32 ^]^ With increasing deformation ratio, the fluorescence intensity began to increase only for the **A‐Int** specimen, indicating the mechanical activation of SP to the fluorescent MC form. Interestingly, depending on the characteristics of attached SPs, the mechanical properties of xPMA were slightly changed. Both **A‐Int** and **C‐I** samples showed improved mechanical properties in terms of ultimate stress and the maximum deformation ratio compared to **C‐II** that cannot form covalent linkages with the PMA matrix. Other researchers have also confirmed that the formation of strong covalent bonds between filler and matrix enhances the mechanical properties of composites.^[^ 33 ^]^ Interestingly, mechanophore‐linked composites have the highest values for the deformation ratio at failure and tensile strength. We hypothesize that the increase in strain energy is due to the early breaking of intrinsically weaker bonds (C–O spiro bond in this system) prior to the main bonds, causing the material to dissipate more energy before the rupture. This toughening mechanism has been reported previously using multiple‐network gels^[^ 34, 35, 36 ^]^ and mechanophore‐incorporated hydrogels.^[^ 37 ^]^

We further investigated the effect of the amount of SP‐functionalized SiO_2_ on the mechanical properties of the xPMA composites (Figure S11 and S12, Supporting Information). All the samples showed increased elastic modulus and tensile strength when higher concentrations of SiO_2_ particles were incorporated. Similarly, **A‐Int** samples have the highest deformation ratio at failure and tensile strength relative to the control SP samples. The strain energy (calculated from the area under the stress–strain curve) was slightly enhanced for **A‐Int** specimens but was more sensitive to particle concentration (Figure S13, Supporting Information). An increase in weight fraction of SP‐functionalized SiO_2_ from 1.5% to 4.5% resulted in a 50% increase in strain energy. We assume that the effect of the mechanophore is minimized due to the significant increase in toughness by introducing more nanoparticles in the elastomeric composites.

We compared the mechanical reactivity of SP functionalized at the interface between the SiO_2_ particles and the polymer matrix (interfacial activation, **A‐Int**) with that of SP covalently linked to a bulk polymer (bulk activation, **A‐Bulk**). To maintain similar cross‐linking density for interfacial activation and bulk activation of the xPMA composites, we characterized the amount of attached SPs for active (**2**) and control (**5**) particles using TGA (Figure S14, Supporting Information). Under the same synthetic condition, the amount of attached SPs was estimated as 2.5–3 wt%. A similar concentration of **3** was incorporated in xPMA for bulk activation. The overall cross‐linking density was maintained by controlling the amount of EGDMA.

We measured the mechanical and fluorescence behaviors of **A‐Int** and **A‐Bulk** samples with varying cross‐linking density (0.5, 1.5, and 3.0 mol%) as a function of deformation ratio (**Figure** **3**a). The mechanical properties of the two specimen types were similar (Figure S15 and S16, Supporting Information). Following the work of Beiermann et al.,^[^ 19 ^]^ the onset of activation was defined as the lowest deformation ratio with a positive slope of fluorescence intensity versus deformation ratio, as indicated with red arrows.

**Figure 3:**
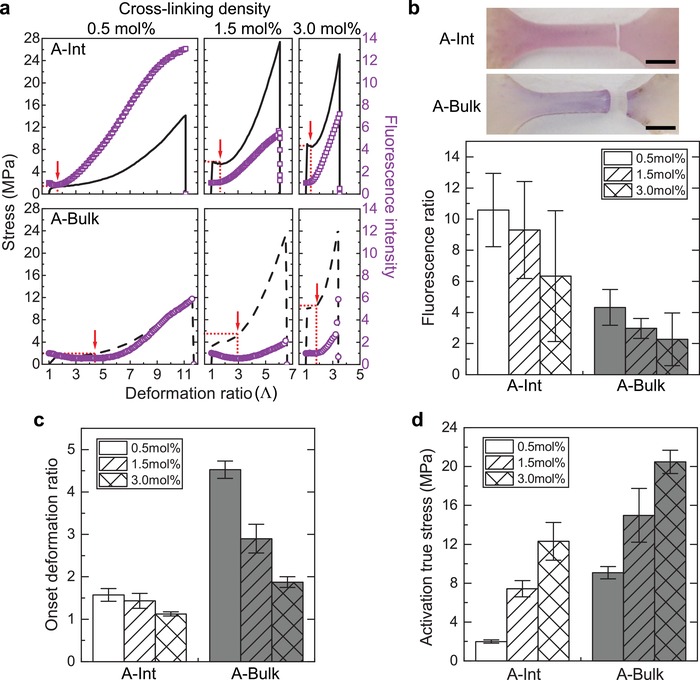
Comparison of mechanical activation behavior of SP depending on attached phases. a) Combined stress and fluorescence intensity data as a function of deformation ratio for **A‐Int** (upper row) and **A‐Bulk** (lower row). The same amount of SP‐functionalized SiO_2_ (1.5 wt%) was incorporated and the overall cross‐linking density was controlled by the amount of EGDMA. The red arrows indicate the activation onset point that is defined where the derivative of fluorescence intensity to deformation ratio starts to change a positive value. b) Images of **A‐Int** and **A‐Bulk** incorporated samples (1.5 mol% cross‐linking density) after failure (upper). The change in fluorescence ratio (maximum fluorescence value/initial fluorescence value) with different cross‐linking density for **A‐Int** and **A‐Bulk** (lower). Scale bar = 2 mm. c) Activation onset deformation ratio and d) activation onset true stress for **A‐Int** and **A‐Bulk** with different cross‐linking density.

We found that more SP was mechanically activated when located at the interface between the filler and the polymeric matrix (Figure 3b). The optical images taken right after failure also revealed that more intense color was generated for **A‐Int** samples. The average fluorescence intensity was calculated for each case by dividing the maximum fluorescence intensity by the initial fluorescence value. Regardless of cross‐linking density, interfacial activation resulted in a higher fluorescence ratio than bulk activation, indicating more efficient mechanical activation of SP.

In addition, **A‐Int** activated at a lower deformation ratio than **A‐Bulk** (Figure 3c, Movie S1, Supporting Information). Increasing cross‐linking density reduced the onset deformation ratio for both **A‐Int** and **A‐Bulk** specimens. However, **A‐Bulk** specimens showed a more dramatic change in the onset deformation ratio than **A‐Int**. We hypothesize that chain alignment (associated with larger activation strain) is more critical for SP directly linked to the polymer.^[^ 19, 38 ^]^ Increased cross‐linking density leads to an increase in stiffness and reduction in polymer chain mobility, ultimately leading to reduced mechanophore activation prior to rupture (Figure 3b).^[^ 39, 40 ^]^ We also compared corresponding activation stress and true stress values at onset deformation ratio for **A‐Int** and **A‐Bulk** (Table S2, Supporting Information, Figure 3d). **A‐Int** required significantly lower true stress values for mechanical activation compared with **A‐Bulk**. These data provide direct evidence that mechanophores at the interfaces of composites activate more efficiently than mechanophores linked in bulk polymers.

The enhanced mechanical reactivity of mechanophores is attributed to the high stress concentration at the matrix/particles interface. Finite element (FE) simulations were performed to analyze the stress concentration phenomenon at the PMA/SiO_2_ interface (computational details can be found in the Supporting Information). The FE computational approach was chosen over theoretical analysis^[^ 41 ^]^ to consider the effect of both the hyperelastic mechanical behavior of PMA, and the interaction between clustered SiO_2_ particles. A generic area was selected from the TEM image of the cross section of the xPMA/SiO_2_ composite (**Figure** **4**a) and then modeled for FE simulations. A linear displacement was applied to the two free edges along the *y* direction until reaching a final deformation ratio of 3.5. We observed a significant stress concentration at the interfacial region along the loading direction (Figure 4b). The stress value recorded at the particle/matrix interface was up to 50 times higher than that in the bulk matrix. The stress concentration was enhanced in the clustered regions of SiO_2_. A similar trend was observed for the normalized strain contour plot (Figure S17, Supporting Information). We believe the large difference in the activation onset between **A‐Int** and **A‐Bulk** shown in Figure 3 can be attributed to the significant stress/strain concentration at the interfacial regions. In addition, a mapping of the first invariant of the Cauchy Green tensor (*I*_1_) was calculated to describe the increased fluorescence intensity for **A‐Int** (Figure 4c). Wang et al. demonstrated that the *I*_1_ profile is directly proportional to the fluorescence intensity mapping of highly deformed elastomers containing SP.^[^ 42 ^]^ For xPMA/SiO_2_ composites under tension, high *I*_1_ values were observed at the interfacial regions (i.e., up to seven times higher than the values in the PMA matrix). The *I*_1_ concentration observed in FE simulations is highly correlated with the higher fluorescence intensity ratio of **A‐Int** measured during experimental testing (Figure 3b).

**Figure 4:**
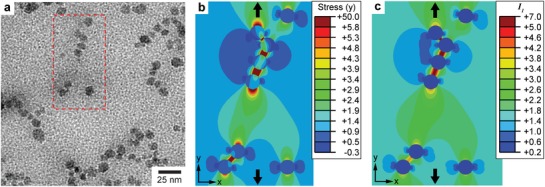
Enhanced activation and stress concentration at the interface between SiO_2_ nanoparticles and PMA matrix under tension. a) TEM image of cross section of the xPMA/SiO_2_ composites. The region in the red box was selected for FE simulations. b) Normalized stress (y) contour plot for xPMA/SiO_2_ composites under tension. Arrows indicate the loading direction. c) Normalized first invariant of the Cauchy Green tensor (*I*_1_) for xPMA/SiO_2_ composites under tension.

In conclusion, we demonstrated the mechanical activation of SP at the interface between PMA and SiO_2_ nanoparticles in solution and solid states. To incorporate SP at the composite interface, we synthesized asymmetrically functionalized SP and optimized the surface functionalization condition of SP on SiO_2_ nanoparticles. Linear or xPMA was grown from the opposing side of the spiro junction enabling force transmission across the spiro C–O bond. With cross‐linkable SP‐functionalized nanoparticles, xPMA nanocomposites were fabricated and the change in optical properties was measured using an in situ optical/mechanical testing setup. In addition, two mechanical activation modes, interfacial activation (**A‐Int**) and bulk activation (**A‐Bulk**), were compared to demonstrate the interfacial force‐focusing effects. For the case of interfacial activation, more SP mechanophores mechanically activated and the threshold for activation were significantly reduced compared to bulk activation. Our experimental observations were supported by the computational simulations, which predicted large stress concentrations and enhanced activation at the nanoparticle interfaces. This interfacial force‐focusing strategy has potential to enhance the mechanical reactivity of mechanophores and import improved damage‐sensing capabilities to polymer composites.

## Experimental Section

##### Synthesis of xPMA/SiO_2_ Composites

The xPMA composite (cross‐linking density = 0.5 mol%) containing SP at nanoparticle interfaces in the composite (**A‐Int**) was prepared by the following methods. Benzoyl peroxide (16.96 mg, 0.07 mmol, 1 eqiv.) and methacrylate‐functionalized SiO_2_ particles (SiO_2_@**2**, 30 mg, 1.3 × 10^−3^ mmol of SP‐MA attached) were combined in a sealed vial and several cycles of vacuum purging and backfilling with N_2_ were conducted. Then, methyl acrylate (2 mL, 22.21 mmol, 336 eqiv.) was added to the vial and the solution was sonicated until it became a well‐dispersed solution. EGDMA (22 µL, 0.12 mmol, 1.68 eqiv.) and *N*,*N*‐dimethylaniline (4 µL, 0.032 mmol, 0.45 eqiv.) were added to the vial and sonicated.

The xPMA (0.5 mol%) containing SP at the PMA matrix (**A‐Bulk**) was fabricated using the similar method. Benzoyl peroxide (16.96 mg, 0.07 mmol, 1 eqiv.), methacrylate‐functionalized SiO_2_ particles (SiO_2_@**5**, 30 mg), and **3** (1 mg, 1.3 × 10^−3^ mmol, the same amount of active SP from the **A‐Int**) were combined in a sealed vial and several cycles of vacuum purging and backfilling with N_2_ were conducted. Then, methyl acrylate (2 mL, 22.21 mmol, 336 eqiv.) was added to the vial and the solution was sonicated until it became a well‐dispersed solution. EGDMA (21 µL, 0.11 mmol, 1.68 eqiv.) and *N*,*N*‐dimethylaniline (4 µL, 0.032 mmol, 0.45 eqiv.) were added to the vial and sonicated.

The final mixture was injected into the glass molds while flushing with N_2_ in a zip‐lock bag. The samples were stored at least 12 h in a freezer then removed from the mold and washed with methanol to remove residual monomers. The final films were dried in a vacuum oven and laser cut into dog‐bone shaped tensile specimens (gauge length: 5 mm, width: 2 mm, thickness: 0.7 mm).

##### Mechanical Activation Tests under Tension

Tensile specimens were tested with a combined mechanical and optical testing setup.^[^ 19 ^]^ A 532 nm laser (0.6 mW) was used for the excitation light source for fluorescence measurements. The specimen was uniaxially deformed at a strain rate of 0.1 s^−1^ by two opposing actuators. Load was recorded using a 50 lb capacity load cell (Honeywell Sensotec) attached to one of the actuators. Excitation light was excluded by a long‐pass emission filter (580 nm cutoff) and fluorescence images were captured with a color CCD (AVT Stingray F504c) on every second. The acquired load–displacement data were converted to engineering stress and deformation ratio. Fluorescence intensity was calculated by averaging the red channel intensity of the gauge section of the sample.

## Conflict of Interest

The authors declare no conflict of interest.

## Supporting information

Supporting Information

Supplemental Movie 1
