# Supplementary material for: Interfacial Force‐Focusing Effect in Mechanophore‐Linked Nanocomposites
Source: Adv Sci (Weinh). 2020 Feb 26;7(7):1903464. doi: 10.1002/advs.201903464 (PMC7141001; doi:10.1002/advs.201903464)
Supplement: Supplementary file 1 — Supporting Information [file ADVS-7-1903464-s001.pdf]

Copyright WILEY-VCH Verlag GmbH & Co. KGaA, 69469 Weinheim, Germany, 2020.

Supporting Information

for *Adv. Sci.,* DOI: 10.1002/advs.201903464

Interfacial Force-Focusing Effect in Mechanophore-Linked Nanocomposites

*Tae Ann Kim, Caterina Lamuta, Hojun Kim, Cecilia Leal, and Nancy R. Sottos**

Copyright WILEY-VCH Verlag GmbH & Co. KGaA, 69469 Weinheim, Germany, 2018.

Supporting Information

Interfacial Force-focusing Effect in Mechanophore-linked Nanocomposite

*Tae Ann Kim, Caterina Lamuta, Hojun Kim, Cecilia Leal, Nancy R. Sottos**

Table of Contents

1. General Experimental Details 1
1.1 Materials 1
1.2 Characterization 1
2. Small Molecules Synthesis 3
2.1 Mechanically active spiropyran (SP) 3
2.2 Control SPs 9
2.3 Di-functionalized mechanically active SP (MA-SP-MA) 15
2.4 NMR spectra 17
3. Surface Functionalization of SiO_2 Particles 31
3.1 Instability of SP in ammonia solution 31
3.2 Optimized procedure for surface functionalization with SP 31
4. Surface-initiated polymerization and composite 33
4.1 Surface-initiated SET-LRP from SP functionalized SiO_2 particles 33
4.1.1 Surface-initiated polymerization from micron-sized particles 33
4.1.2 TEM images of bare SiO_2 nanoparticle 34
4.2 Calculation of grafting density 35
4.3 Characterization of glass transition temperature (T_g) of PMA 36
4.4 Solution activation 38
4.5 Composites from linear PMA and SP functionalized SiO_2 nanoparticles 39
4.5.1 Fabrication method 39
4.5.2 Tension test 39
5. Mechanophore-linked cross-linked PMA 41
5.1 Cross-linked PMA (xPMA) with SP-functionalized SiO_2 particles 41
5.1.1 Polymerization procedure 41
5.1.2 Tensile activation test: Optical and fluorescence images 41
5.1.3 Effect of the amount of SiO_2 on the mechanical properties 42
5.2 Comparison of the mechanical reactivity of A-Int to that of A-Bulk. 44
5.2.1 Characterization of attached SP to SiO_2 particles 44
5.2.2 Polymerization procedure for bulk specimens 44
5.2.3 Mechanical properties of A-Int and A-Bulk specimens with increasing concentrations of crosslinker 46
5.2.4 Activation stress and activation true stress 48
6. Finite Elements Smulations 49
7. References 50

1. General Experimental Details

1.1 Materials

Unless otherwise states, all reagents were purchased from commercial source and used as received. Deuterated solvents (chloroform-d, dimethyl sulfoxide-d_6) were purchased from Cambridge Isotope Laboratories, Inc. Methyl acrylate (MA) was passed through a basic alumina filled column to remove inhibitors and subsequent bubbling with nitrogen to eliminate any oxygen. Copper wire was purchased from Fisher scientific. Micron-sized SiO_2 particles were purchased from Polysciences, Inc. SiO_2 nanoparticles were donated by Nissan Chemical in a dispersion of SiO_2 nanoparticles with a size of 10–15 nm in methyl isobutyl ketone (30–31 wt% SiO_2)

1.2 Characterization

Column chromatography was performed on a Biotage Isolera system using SiliCycle SiliaSep HP flash cartridges. NMR spectra were recorded using a Varian 500 MHz spectrometer or Carver-Bruker 500 spectrometer. Spectra were referenced to the residual solvent peak: Dimethyl sulfoxide (^{1}H NMR: 2.50 ppm, ^{13}C NMR: 39.52 ppm) or chloroform (^{1}H NMR: 7.24 ppm, ^{13}C NMR: 77.23 ppm). Mass spectra were obtained from the Mass Spectrometry Laboratory, School of Chemical Sciences, at the University of Illinois.

Thermal gravimetric analysis (TGA) was performed using TA instrument Q500 under a nitrogen atmosphere (Flow rate: 90.0 ml/min). 5–10 mg of a sample in a platinum pan was equilibrated at 40 °C and the temperature was ramped up to 800 °C at a rate of 20 °C/min.

Gel permeation chromatograph (GPC) analyses were performed with a Waters 1515 Isocratic HPLC pump, a Waters (2998) Photodiode Array Detector, a Waters (2414) Refractive Index Detector, a Waters (2707) 96-well autosampler, and a series of 4 Waters HR Styragel columns (7.8 × 300mm, HR1, HR3, HR4, and HR5) in THF at 30 °C. The instrument was calibrated with monodisperse polystyrene standards.

Differential scanning calorimetry (DSC) experiments were conducted using a TA instruments Q20 DSC. The ramping rate of temperature was fixed to 10 °C. At first, the samples were heated to 80 °C and cooled to -20 °C. Glass transition temperature (T_g) was obtained from the second heating cycle from -20 °C to 80 °C.

Ultrasound experiments were performed on a Vibra Cell 505 liquid processor with a diameter solid probe from Sonics and Materials. The distance between the titanium tip and bottom of the Suslick cell was 1 cm. The Suslick cells were made by the School of Chemical Sciences'

Glass Shop at the University of Illinois. UV-Vis spectra were recorded using a Shimadzu UV-2401PC.

Digital images of tensile specimens were taken using a digital camera (Canon G16, 12.1 megapixels). Fluorescence images were acquired with a confocal microscope (Leica TCS SP8). The surface morphologies of micron-sized SiO_2 particles were observed with an environmental scanning electron microscope (ESEM, FEI Quanta FEG 450). Transmission electron microscope (TEM) micrographs were taken using a JEOL 2100 cryo-TEM at an accelerating voltage of 120 kV. For examining the cross-sectional morphology of composites, the specimen was sectioned using a Leica microtome Ultracut UCT and placed on copper grids.

2. Small Molecules Synthesis

2.1 Mechanically active spiropyran (SP)

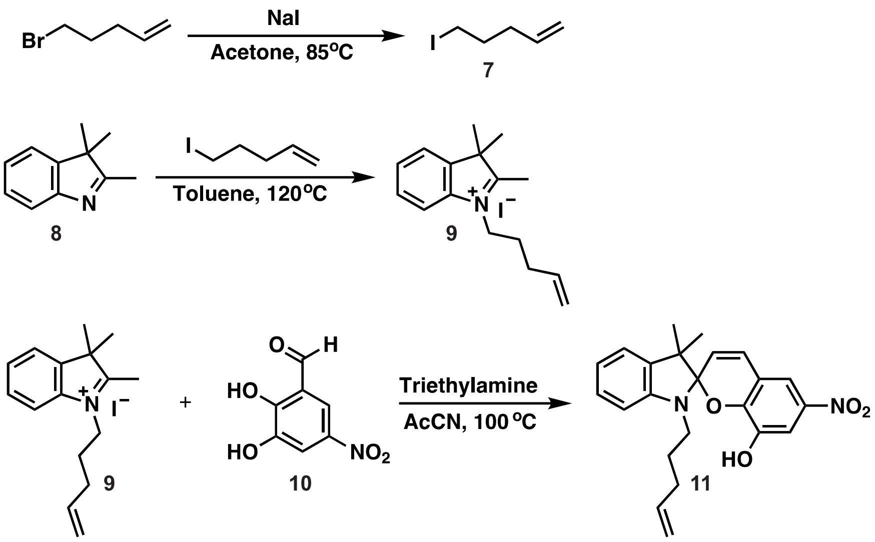

Scheme 1. Synthesis of asymmetrically functionalized SP **11**

Synthesis of 5-iodopent-1-ene (**7**)

NaI (51.0 g, 338 mmol, 2 equiv) was dispersed in 300 ml of dry acetone under N_2 atmosphere. 5-bromo-1pentene (20.0 ml, 169 mmol, 1 equiv) was added to the solution and refluxed at 85 °C for 2 h. Precipitation was formed and the solution became yellow dispersion. The mixture was cooled to room temperature and diluted with 100 ml of distilled water. The product was extracted with dichloromethane three times, washed with brine, and dried over sodium sulfate. After filtering, the filtrate was concentrated *in vacuo* to yield a slightly yellowish liquid (27.7 g, 141 mmol, 84%). ^{1}H NMR (500 MHz, $CDCl_3$): δ 5.75 (ddt, *J* = 17.0, 10.2, 6.7 Hz, 1H), 5.13 – 4.97 (m, 2H), 3.19 (t, *J* = 6.9 Hz, 2H), 2.17 (dtt, *J* = 7.6, 6.5, 1.4 Hz, 2H), 2.00 – 1.83 (m, 2H). ^{13}C NMR (126 MHz, $CDCl_3$): δ 136.68, 116.06, 34.42, 32.56, 6.45.

Synthesis of 2,3,3-trimethyl-1-(pent-4-en-1yl)-3H-indol-1-ium iodide5-iodipent-1-ene (**9**)

8 (2.5 g, 16 mmol, 1 equiv) was dissolved in 18 ml of dry toluene, followed by adding **7** (4.0 ml, 33 mmol, 2 equiv) to the solution. After refluxing at 120 °C for 23h, the solution was cooled in an ice bath. The precipitate was collected and washed with toluene and cold diethyl ether several times. Further drying under high vacuum yielded redish powder (4.6 g, 13 mmol, 82%)

^1H NMR (500 MHz, d_6-DMSO) δ 7.98 (d, *J* = 6.7 Hz, 1H), 7.93 – 7.77 (m, 1H), 7.72 – 7.52 (m, 2H), 5.87 (dd, *J* = 17.2, 9.3 Hz, 1H), 5.06 (dd, *J* = 29.3, 13.8 Hz, 2H), 4.45 (t, *J* = 7.7 Hz, 2H), 2.84 (s, 3H), 2.22 (q, *J* = 7.2 Hz, 2H), 2.06 – 1.85 (m, 2H), 1.54 (s, 6H). ^{13}C NMR (126 MHz, d_6-DMSO) δ 196.61, 141.85, 141.05, 137.14, 129.41, 128.95, 123.52, 115.85, 115.40, 54.18, 47.20, 29.90, 26.17, 22.01, 14.02. HRMS-ESI (m/z): [M+H-I] calcd for $C_{16}H_{22}N$, 228.1752; found, 228.1756.

Synthesis of 2,3-dihydroxy-5-nitro-benzaldehyde (**10**)

Prepared according to literature procedure.[1]

^1H NMR (400 MHz, d_6-DMSO): δ 11.19 (br, 2H), 10.29 (s, 1H), 7.97 (d, *J* = 2.8 Hz, 1H), 7.76 (d, *J* =3.2 Hz, 1H). ^{13}C NMR (126 MHz, d_6-DMSO): δ 189.8, 156.0, 147.2, 139.2, 121.8, 114.6, 113.2. HRMS-EI (m/z): $[M]^+$ calcd for $C_7H_5NO_5$, 183.0168; found, 183.0164.

Synthesis 3',3'-dimethyl-6-nitro-1'-(pent-4-en-1-yl)spiro[chromene-2,2'-indolin]-8-ol (**11**)

A round-bottom flask equipped with a condenser, and N_2 inlet adapter was charged with **9** (0.89 g, 2.5 mmol, 1 equiv), **10** (0.46 g, 2.5 mmol, 1 equiv), and 6 ml of acetonitrile. Then, triethylamine (0.37 ml, 2.6 mmol, 1.1 equiv) was added and heated to reflux at 100 °C for 4h. Once the solution was cooled to room temperature, the flask wrapped with aluminum foil kept in a freezer overnight. The precipitate was filtered and washed with minimum amount of cold acetonitrile, followed by drying at 40 °C in a high vacuum oven. **11** was collected as red needles (0.41 g, 1.0 mmol, 40%).

^1H NMR (500 MHz, d_6-DMSO, HCl(g)) δ 8.58 (d, *J* = 2.7 Hz, 1H), 8.53 (d, *J* = 16.4 Hz, 1H), 8.02 – 7.97 (m, 1H), 7.92 (d, *J* = 2.5 Hz, 2H), 7.68 – 7.62 (m, 2H), 7.10 (dd, *J* = 8.8, 7.4 Hz, 1H), 6.00 – 5.76 (m, 1H), 5.17 – 4.91 (m, 2H), 4.65 (t, *J* = 7.6 Hz, 2H), 2.23 (t, *J* = 7.2 Hz, 2H), 1.98 (t, *J* = 7.5 Hz, 2H), 1.79 (s, 6H). ^{13}C NMR (126 MHz, d_6-DMSO, HCl(g)) δ 219.86, 191.94, 185.66, 184.20, 181.45, 178.39, 177.20, 174.78, 167.26, 166.84, 160.77, 158.37, 154.99, 153.67, 153.07, 151.89, 149.89, 89.98, 84.22, 67.65, 64.78, 63.58. HRMS-ESI (m/z): [M+H] calcd for $C_{23}H_{25}N_2O_4$, 393.1814; found, 393.1814.

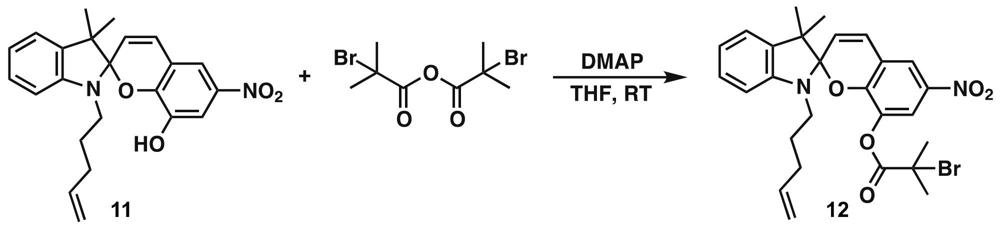

Scheme 2. Synthesis of asymmetrically functionalized SP for a SET-LRP initiator.

<u>Synthesis of 3',3'-dimethyl-6-nitro-1'-(pent-4-en-1-yl)spiro[chromene-2,2'-indolin]-8-yl 2-bromo-2-methylpropanoate (**12**)</u>

11 (600 mg, 1.53 mmol, 1 equiv) and 4-dimethylaminopyridine (169 mg, 1.38 mmol, 0.9 equiv) were dissolved in 20 ml of THF. Then, a solution of 2-bromo-2-methylpropanic anhydride (625 mg, 1.99 mmol, 1.3 equiv) in 10 ml of THF was added dropwise to the mixture and stirred overnight. The solvent was completely removed and the crude product was purified by column chromatography (0–25% EtOAc/hexane). Recrystallization from boiling hexane yielded **12** as greenish yellow crystals (594 mg, 1.10 mmol, 72%).

TLC (25% EtOAc/Hexane) R_f: 0.75 (UV)

^{1}H NMR (500 MHz, $CDCl_3$): δ 7.97 (d, *J* = 2.7 Hz, 1H), 7.91 (d, *J* = 2.6 Hz, 1H), 7.11 (td, *J* = 7.7, 1.3 Hz, 1H), 7.06 – 7.00 (m, 1H), 6.96 (d, *J* = 10.5 Hz, 1H), 6.82 (td, *J* = 7.4, 1.0 Hz, 1H), 6.53 (d, *J* = 7.8 Hz, 1H), 5.94 (d, *J* = 10.4 Hz, 1H), 5.78 (ddt, *J* = 16.8, 10.2, 6.5 Hz, 1H), 5.04 – 4.93 (m, 2H), 3.12 – 3.00 (m, 2H), 2.14 – 1.97 (m, 2H), 1.78 – 1.57 (m, 2H), 1.52 (s, 3H), 1.48 (s, 3H), 1.25 (s, 5H), 1.20 (s, 3H). ^{13}C NMR (126 MHz, $CDCl_3$): δ 169.15, 151.08, 147.18, 140.14, 138.06, 137.37, 136.20, 128.37, 127.83, 121.96, 121.67, 120.48, 119.86, 119.56, 119.12, 115.18, 108.27, 107.25, 54.02, 52.35, 43.36, 31.75, 31.41, 30.32, 30.23, 28.25, 26.09, 22.81, 19.40, 14.28. HRMS-ESI (m/z): [M+H] calcd for $C_{27}H_{30}N_2O_5Br$, 541.1338; found, 541.1337.

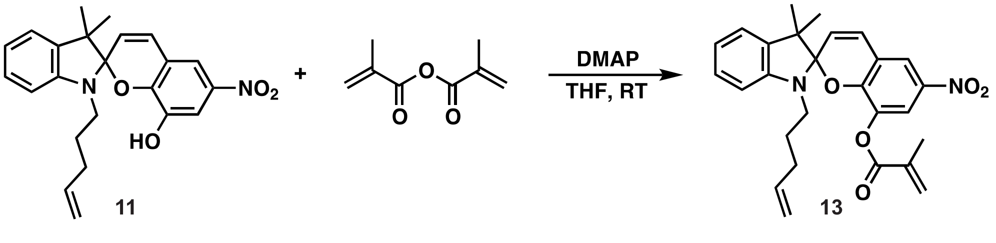

Scheme 3. Synthesis of asymmetrically functionalized SP for a cross-linker.

Synthesis of 3',3'-dimethyl-6-nitro-1'-(pent-4-en-1-yl)spiro[chromene-2,2'-indolin]-8-yl methacrylate (**13**)

To a solution of **11** (400 mg, 1.0 mmol, 1 equiv) and 4-dimethylaminopyridine (110 mg, 0.92 mmol, 0.9 equiv) in 10 ml of THF was added methacrylic anhydride (200 μL, 1.3 mmol, 1.3 equiv) dropwise. The solution was stirred overnight and the solvent was completely evaporated. The crude product was purified by column chromatography (0–5% EtOAc/hexane) to yield **13** as a purple, viscous oil (340 mg, 0.74 mmol, 72%).

TLC (5% EtOAc/Hexane) R_f: 0.33 (UV)

^{1}H NMR (500 MHz, $CDCl_3$): δ 7.95 (d, *J* = 2.6 Hz, 1H), 7.90 (d, *J* = 2.6 Hz, 1H), 7.10 (td, *J* = 7.7, 1.3 Hz, 1H), 7.00 (dd, *J* = 7.2, 1.2 Hz, 1H), 6.94 (d, *J* = 10.4 Hz, 1H), 6.80 (td, *J* = 7.4, 0.9 Hz, 1H), 6.51 (d, *J* = 7.7 Hz, 1H), 5.93 (d, *J* = 10.4 Hz, 1H), 5.87 (t, *J* = 1.0 Hz, 1H), 5.77 (ddt, *J* = 16.9, 10.2, 6.6 Hz, 1H), 5.37 (t, *J* = 1.5 Hz, 1H), 5.01 (q, *J* = 1.7 Hz, 1H), 4.99 – 4.92 (m, 1H), 3.13 – 2.96 (m, 2H), 2.13 – 1.97 (m, 3H), 1.75 – 1.66 (m, 1H), 1.63 (dd, *J* = 1.6, 1.0 Hz, 3H), 1.24 (s, 3H), 1.20 (s, 3H). ^{13}C NMR (126 MHz, $CDCl_3$): δ 165.00, 151.11, 147.00, 140.16, 138.18, 138.17, 136.16, 134.75, 128.30, 127.59, 127.42, 121.86, 121.44, 119.97, 119.61, 119.42, 119.27, 115.08, 107.75, 107.12, 52.12, 43.25, 31.43, 28.25, 25.90, 19.65, 17.89. HRMS-ESI (m/z): [M+H] calcd for $C_{27}H_{29}N_2O_5$, 461.2076; found, 461.2078.

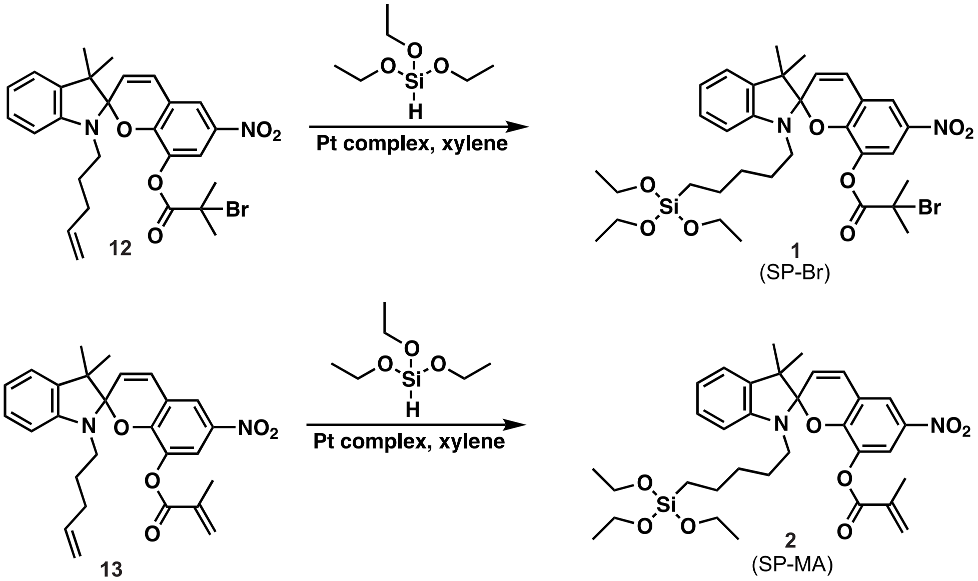

Scheme 4. Synthesis of ethoxysilane functionalized SP for SiO_2 surface functionalization

General procedure for hydrosilylation of alkene-terminated SP

The alkene functionalized SP was dissolved in dry toluene, followed by adding platinum(0)-1,3-divinyl-1,1,3,3-tetramethyldisiloxane complex solution (in xylenes, Pt ~2%, referred to as Pt catalyst solution). Then, triethoxysilane was added to the solution and stirred under N_2 for more than 2 h. The excess triethoxysilane and solvent were removed under vacuum to yield the desired product, which was used without further purification.

Synthesis of 3',3'-dimethyl-6-nitro-1'-(5-(triethoxysilyl)pentyl)spiro[chromene-2,2'-indolin]-8-yl 2-bromo-2-methylpropanoate (**1**)

Following the general procedure, **12** (140 mg, 0.27 mmol, 1 equiv) was dissolved in 5 ml of dry toluene and 15 µL of Pt catalyst solution was added. After an excess triethoxysilane (99 µL, 0.54 mmol, 2 equiv) was added, the mixture was further stirred for 2 h. Evaporation of all the volatile parts from the solution yielded a purple, viscous oil.

^{1}H NMR (500 MHz, $CDCl_3$): δ 7.96 (t, *J* = 2.5 Hz, 1H), 7.90 (d, *J* = 2.6 Hz, 1H), 7.11 (tdd, *J* = 7.7, 4.3, 1.3 Hz, 1H), 7.02 (dt, *J* = 7.4, 1.6 Hz, 1H), 6.99 – 6.92 (m, 1H), 6.81 (dddd, *J* = 9.6, 5.2, 2.2, 0.9 Hz, 1H), 6.56 – 6.49 (m, 1H), 5.99 – 5.86 (m, 1H), 4.00 – 3.50 (m, 8H), 3.18 – 2.94 (m, 2H), 2.37 – 2.04 (m, 2H), 1.64 – 1.43 (m, 8H), 1.28 – 1.15 (m, 15H), 0.75 – 0.42 (m, 2H). ^{13}C NMR (126 MHz, $CDCl_3$): δ 169.13, 151.13, 147.26, 140.09, 137.37, 136.18, 128.29, 127.81, 122.01, 121.62, 120.46, 119.74, 119.56, 119.08, 108.34, 107.21, 58.47, 54.04, 52.32, 43.77, 30.84, 30.32, 30.23, 28.76, 26.11, 22.80, 19.40, 18.47, 10.60. HRMS-ESI (m/z): [M+H] calcd for $C_{33}H_{46}N_2O_8BrSi$, 705.2207; found, 705.2205.

Synthesis of 3',3'-dimethyl-6-nitro-1'-(5-(triethoxysilyl)pentyl)spiro[chromene-2,2'-indolin]-8-yl methacrylate (**2**)

Following the general procedure, **13** (100 mg, 0.22 mmol, 1 equiv) was dissolved in 5 ml of dry toluene and 15 µL of Pt catalyst solution was added. After triethoxysilane (60 µL, 0.33 mmol, 1.5 equiv) was added, the mixture was further stirred for 2 h. Evaporation of all the volatile parts from the solution yielded a purple, viscous oil.

^{1}H NMR (500 MHz, $CDCl_3$): δ 7.94 (d, *J* = 2.6 Hz, 1H), 7.89 (d, *J* = 2.6 Hz, 1H), 7.10 (td, *J* = 7.7, 1.3 Hz, 1H), 7.00 (dd, *J* = 7.3, 1.3 Hz, 1H), 6.94 (d, *J* = 10.4 Hz, 1H), 6.79 (td, *J* = 7.4, 0.9 Hz, 1H), 6.50 (d, *J* = 7.8 Hz, 1H), 5.93 (d, *J* = 10.4 Hz, 1H), 5.89 – 5.84 (m, 1H), 5.38 – 5.34 (m, 1H), 3.79 (q, *J* = 7.0 Hz, 6H), 3.11 – 2.96 (m, 2H), 1.62 (dd, *J* = 1.6, 1.0 Hz, 3H), 1.27 – 1.16 (m, 20H), 0.66

– 0.52 (m, 2H). ^{13}C NMR (126 MHz, $CDCl_3$): δ 164.99, 151.17, 147.10, 140.10, 138.18, 136.15, 134.76, 128.22, 127.56, 127.37, 121.90, 121.38, 119.95, 119.49, 119.43, 119.22, 107.82, 107.09, 58.46, 52.11, 43.66, 30.86, 28.75, 25.93, 22.81, 19.61, 18.46, 17.88, 10.60. HRMS-ESI (m/z): [M+H] calcd for $C_{33}H_{45}N_2O_8Si$, 625.2945; found, 625.2939.

2.2 Control SPs

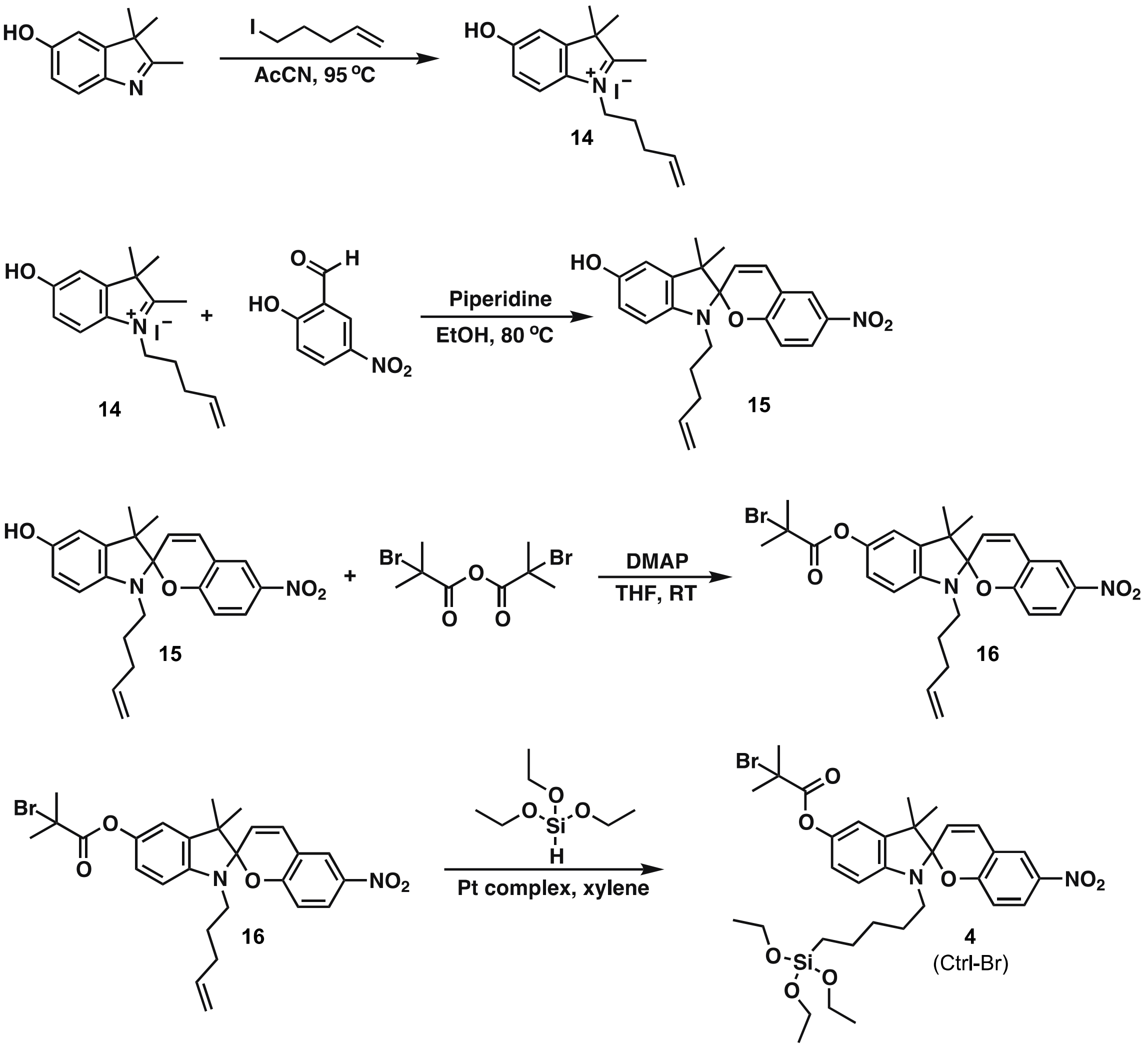

Scheme 5. Synthesis of mechanically inactive SP initiator for SiO_2 surface functionalization

<u>Synthesis of 5-hydroxy-2,3,3-trimethyl-1-(pent-4-en-1-yl)-3H-indol-1-ium iodide (**14**)</u>

A round-bottom flask equipped with a condenser was charged with 2,3,3-trimethyl-3H-indol-5-ol (1.0 g, 5.7 mmol, 1 equiv, prepared from literature procedure[1]) and 10 ml of acetonitrile under N_2 atmosphere. 5-iodo-pent-1-ene (0.93 ml, 7.4 mmol, 1.3 equiv) was added to the solution and stirred at reflux (95 °C) overnight. After the solvent and unreacted reagent were evaporated under vacuum, the solid part was thoroughly washed with toluene and dried under vacuum to yield **14** as purple powders (1.7 g, 4.5 mmol, 79%).

^{1}H NMR (500 MHz, d_6-DMSO) δ 10.28 (s, 1H), 7.77 (d, *J* = 8.8 Hz, 1H), 7.17 (d, *J* = 23.2 Hz, 1H), 6.95 (d, *J* = 8.9 Hz, 1H), 5.85 (ddt, *J* = 17.0, 11.8, 7.0 Hz, 1H), 5.05 (dd, *J* = 30.6, 13.8 Hz,

2H), 4.56 – 4.22 (m, 2H), 2.76 (s, 3H), 2.30 – 2.11 (m, 2H), 1.99 – 1.83 (m, 2H), 1.48 (s, 6H). ^{13}C NMR (126 MHz, d_6-DMSO) δ 192.32, 159.03, 144.02, 137.13, 132.98, 116.47, 115.83, 115.20, 110.47, 53.68, 47.15, 29.87, 26.28, 22.17, 13.80. HRMS-ESI (m/z): [M+H-I] calcd for $C_{16}H_{22}NO$, 244.1701; found, 244.1708.

Synthesis of 3',3'-dimethyl-6-nitro-1'-(pent-4-en-1-yl)spiro[chromene-2,2'-indolin]-5'-ol (**15**)

A round-bottom flask equipped with a condenser, and N_2 inlet adapter was charged with **14** (0.74 g, 2.0 mmol, 1 equiv), 2-hydroxy-5-nitrobenzaldehyde (0.33 g, 2.0 mmol, 1 equiv), and 8 ml of anhydrous ethanol. Then, piperidine (0.21 ml, 2.1 mmol, 1.1 equiv) was added and heated to reflux at 80 °C for 3h. Once the solution was cooled to room temperature, the flask wrapped with aluminum foil kept in a freezer overnight. The precipitate was isolated and washed with minimum amount of cold ethanol. After drying at 40 °C in a high vacuum oven, **15** was collected as dark green powders (0.48 g, 1.2 mmol, 61%).

^{1}H NMR (500 MHz, d_6-DMSO, HCl(g)) δ 8.98 (d, *J* = 2.8 Hz, 1H), 8.35 (d, *J* = 16.4 Hz, 1H), 8.27 (dd, *J* = 9.2, 2.8 Hz, 1H), 7.89 (d, *J* = 16.5 Hz, 1H), 7.80 (d, *J* = 8.8 Hz, 1H), 7.44 (d, *J* = 9.2 Hz, 1H), 7.25 (d, *J* = 2.3 Hz, 1H), 7.06 (dd, *J* = 8.7, 2.3 Hz, 1H), 5.87 (ddt, *J* = 16.8, 10.2, 6.4 Hz, 1H), 5.15 – 5.01 (m, 2H), 4.58 (t, *J* = 7.6 Hz, 2H), 2.29 – 2.14 (m, 2H), 1.97 (qd, *J* = 7.0, 6.0, 3.9 Hz, 2H), 1.75 (s, 6H). ^{13}C NMR (126 MHz, d_6-DMSO, HCl(g)) δ 178.59, 164.25, 159.97, 146.37, 145.03, 140.03, 137.04, 132.71, 128.78, 127.36, 121.49, 117.38, 116.75, 116.16, 116.01, 114.87, 110.06, 51.94, 46.57, 30.01, 27.28, 25.97. HRMS-ESI (m/z): [M+H] calcd for $C_{23}H_{25}N_2O_4$, 393.1814; found, 393.1809.

Synthesis of 3',3'-dimethyl-6-nitro-1'-(pent-4-en-1-yl)spiro[chromene-2,2'-indolin]-5'-yl 2-bromo-2-methylpropanoate (**16**)

15 (120 mg, 0.31 mmol, 1 equiv) and 4-dimethylaminopyridine (34 mg, 0.28 mmol, 0.9 equiv) were dissolved in 5 ml of THF. Then, a solution of 2-bromo-2-methylpropanic anhydride (130 mg, 0.40 mmol, 1.3 equiv) in 2 ml of THF was added dropwise to the mixture and stirred overnight. The solvent was completely removed and the crude product was purified by column chromatography (0–50% EtOAc/hexane). Recrystallization from boiling hexane with minimum amount of THF yielded **16** as yellow crystals (150 mg, 0.28 mmol, 91%).

TLC (50% EtOAc/Hexane) R_f: 0.82 (UV)

^{1}H NMR (500 MHz, $CDCl_3$): δ 8.05 (dd, *J* = 8.9, 2.7 Hz, 1H), 8.02 (d, *J* = 2.7 Hz, 1H), 6.97 – 6.90 (m, 2H), 6.87 (d, *J* = 2.4 Hz, 1H), 6.77 (d, *J* = 8.9 Hz, 1H), 6.54 (d, *J* = 8.3 Hz, 1H), 5.87 (d, *J* = 10.3 Hz, 1H), 5.84 – 5.73 (m, 1H), 5.07 – 4.95 (m, 2H), 3.26 – 3.09 (m, 2H), 2.10 (s, 6H), 1.92 – 1.85 (m, 1H), 1.84 – 1.60 (m, 2H), 1.57 (s, 1H), 1.29 (s, 3H), 1.22 (s, 3H). ^{13}C NMR (126 MHz, $CDCl_3$): δ 171.04, 159.59, 145.22, 144.07, 141.15, 137.90, 137.36, 128.45, 126.12, 122.88, 121.76, 119.79, 118.53, 115.69, 115.32, 115.22, 107.10, 106.74, 55.78, 52.82, 43.49, 31.43, 30.87, 28.10, 25.98, 19.90. HRMS-ESI (m/z): [M+H] calcd for $C_{27}H_{30}N_2O_5Br$, 541.1338; found, 541.1351.

Synthesis of 3',3'-dimethyl-6-nitro-1'-(5-(triethoxysilyl)pentyl)spiro[chromene-2,2'-indolin]-5'-yl 2-bromo-2-methylpropanoate (**4**)

Following the general procedure for the hydrosilylation, **16** (96 mg, 0.18 mmol, 1 equiv) was dissolved in 3 ml of dry toluene and 15 µL of Pt catalyst solution was added. After triethoxysilane (65 µL, 0.36 mmol, 2 equiv) was added, the mixture was further stirred for 2 h. Evaporation of all the volatile parts from the solution yielded a purple, viscous oil.

^{1}H NMR (500 MHz, $CDCl_3$): δ 8.02 (ddd, *J* = 8.9, 2.7, 1.2 Hz, 1H), 7.99 (q, *J* = 2.7, 2.0 Hz, 1H), 6.94 – 6.86 (m, 2H), 6.84 (td, *J* = 2.4, 1.0 Hz, 1H), 6.74 (d, *J* = 8.9 Hz, 1H), 6.51 (ddd, *J* = 8.4, 2.8, 1.1 Hz, 1H), 5.92 – 5.74 (m, 1H), 3.98 – 3.69 (m, 7H), 3.25 – 2.95 (m, 1H), 2.07 (d, *J* = 1.1 Hz, 6H), 1.73 – 1.47 (m, 2H), 1.44 – 1.03 (m, 19H), 0.68 – 0.44 (m, 2H). ^{13}C NMR (126 MHz, $CDCl_3$): δ 171.05, 159.60, 145.32, 143.91, 141.02, 137.31, 128.36, 126.08, 122.86, 121.80, 119.74, 118.50, 115.66, 115.15, 107.12, 106.68, 58.46, 55.78, 52.79, 43.95, 30.84, 28.66, 25.93, 22.77, 19.87, 18.46, 18.13, 10.53. HRMS-ESI (m/z): [M+H] calcd for $C_{33}H_{46}N_2O_8BrSi$, 705.2207; found, 705.2197.

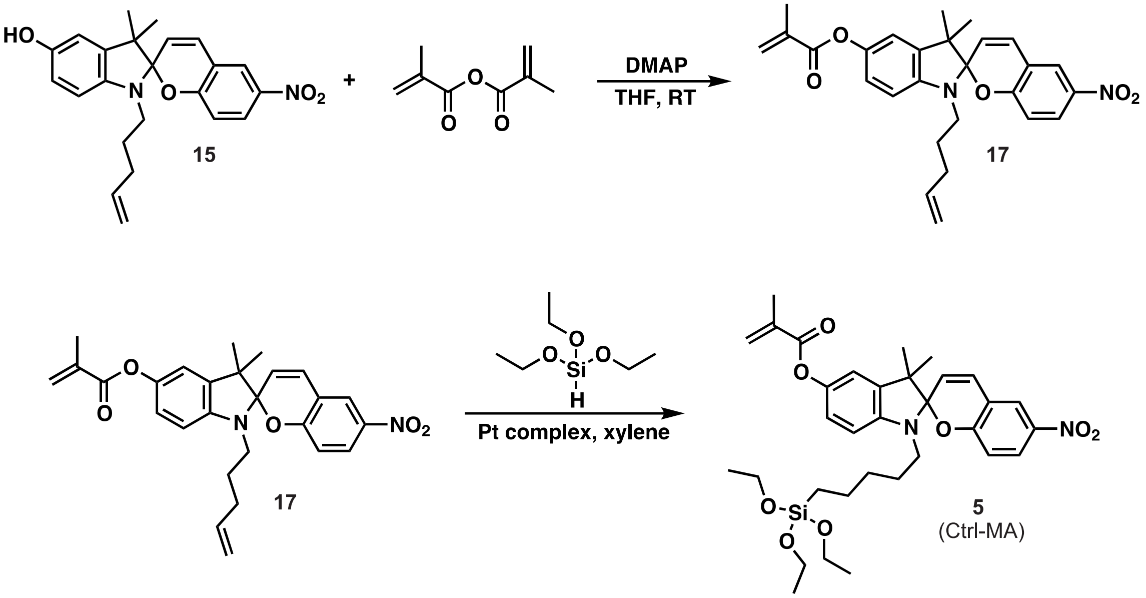

Scheme 6. Synthesis of mechanically inactive SP cross-linker for SiO_2 surface functionalization

Synthesis of 3',3'-dimethyl-6-nitro-1'-(pent-4-en-1-yl)spiro[chromene-2,2'-indolin]-5'-yl methacrylate (**17**)

To a solution of **15** (200 mg, 0.51 mmol, 1 equiv) and 4-dimethylaminopyridine (56 mg, 0.46 mmol, 0.9 equiv) in 5 ml of THF was added methacrylic anhydride (99 μL, 0.66 mmol, 1.3 equiv) dropwise. The solution was stirred overnight and the solvent was completely evaporated. The crude product was purified by column chromatography (0–50% EtOAc/hexane) to yield **17** as a yellowish powder (120 mg, 0.26 mmol, 51%).

TLC (50% EtOAc/Hexane) R_f: 0.5 (UV)

^{1}H NMR (500 MHz, $CDCl_3$): δ 8.03 (dd, *J* = 8.9, 2.7 Hz, 1H), 8.00 (d, *J* = 2.6 Hz, 1H), 6.91 (d, *J* = 2.3 Hz, 1H), 6.90 – 6.88 (m, 1H), 6.85 (d, *J* = 2.4 Hz, 1H), 6.75 (d, *J* = 8.9 Hz, 1H), 6.52 (d, *J* = 8.3 Hz, 1H), 6.34 (t, *J* = 1.2 Hz, 1H), 5.85 (d, *J* = 10.4 Hz, 1H), 5.81 – 5.71 (m, 2H), 5.04 – 4.93 (m, 2H), 3.23 – 3.06 (m, 2H), 2.16 – 1.98 (m, 5H), 1.82 – 1.59 (m, 2H), 1.25 (s, 3H), 1.19 (s, 3H).

^{13}C NMR (126 MHz, $CDCl_3$): δ 166.70, 159.65, 144.93, 144.12, 141.03, 137.94, 137.22, 136.17, 128.39, 127.10, 126.10, 122.87, 121.84, 120.30, 118.51, 115.82, 115.69, 115.28, 107.12, 106.76, 52.80, 43.49, 31.44, 28.07, 25.99, 19.90, 18.63. HRMS-ESI (m/z): [M+H] calcd for $C_{27}H_{29}N_2O_5$, 461.2076; found, 461.2085.

Synthesis of 3',3'-dimethyl-6-nitro-1'-(5-(triethoxysilyl)pentyl)spiro[chromene-2,2'-indolin]-5'-yl methacrylate (**5**)

Following the general procedure for the hydrosilylation, **17** (60 mg, 0.13 mmol, 1 equiv) was dissolved in 5 ml of dry toluene and 15 µL of Pt catalyst solution was added. After triethoxysilane (72 µL, 0.39 mmol, 3 equiv) was added, the mixture was further stirred for 2 h. Evaporation of all the volatile parts from the solution yielded a yellowish brown oil.

^{1}H NMR (500 MHz, $CDCl_3$): δ 8.02 (dd, *J* = 9.0, 2.7 Hz, 1H), 7.99 (d, *J* = 2.8 Hz, 1H), 6.91 – 6.88 (m, 2H), 6.84 (d, *J* = 2.3 Hz, 1H), 6.75 (d, *J* = 9.0 Hz, 1H), 6.51 (d, *J* = 8.3 Hz, 1H), 6.35 – 6.31 (m, 1H), 5.84 (d, *J* = 10.3 Hz, 1H), 5.74 (t, *J* = 1.5 Hz, 1H), 3.79 (q, *J* = 7.0 Hz, 6H), 3.21 – 3.03 (m, 2H), 2.07 (t, *J* = 1.3 Hz, 4H), 1.71 – 1.48 (m, 3H), 1.39 (ddd, *J* = 7.7, 5.2, 2.5 Hz, 1H), 1.36 – 1.11 (m, 17H), 0.65 – 0.52 (m, 2H). ^{13}C NMR (126 MHz, $CDCl_3$): δ 166.72, 159.71, 145.03, 144.05, 140.99, 137.20, 136.19, 128.30, 127.08, 126.07, 122.85, 121.91, 120.28, 118.52, 115.77, 115.68, 107.17, 106.72, 58.47, 52.81, 52.79, 43.97, 30.87, 28.68, 26.01, 22.77, 19.90, 18.63, 18.46, 10.54. HRMS-ESI (m/z): [M+H] calcd for $C_{33}H_{45}N_2O_8Si$, 625.2945; found, 625.2946.

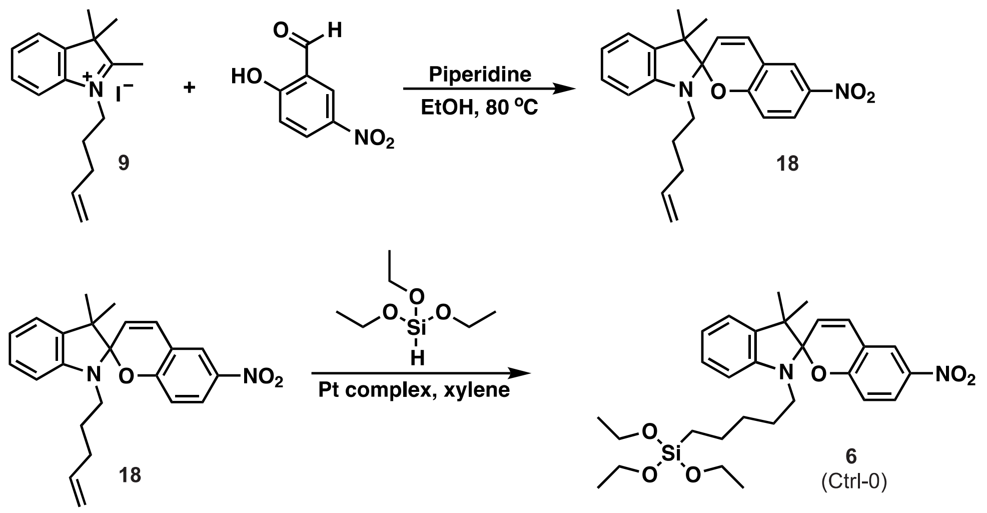

Scheme 7. Synthesis of mono-functionalized SP for SiO_2 surface functionalization

Synthesis of 3',3'-dimethyl-6-nitro-1'-(pent-4-en-1-yl)spiro[chromene-2,2'-indoline] (**18**)

A round-bottom flask equipped with a condenser, and N_2 inlet adapter was charged with **9** (0.36 g, 1.0 mmol, 1 equiv), 2-hydroxy-5-nitrobenzaldehyde (0.17 g, 1.0 mmol, 1 equiv), and 5 ml of anhydrous ethanol. Then, piperidine (0.10 ml, 1.1 mmol, 1.1 equiv) was added and heated to reflux at 80 °C for 3h. Once the solution was cooled to room temperature, the flask wrapped with aluminum foil kept in a freezer overnight. The precipitate was filtered and washed with minimum

amount of cold ethanol. After drying at 40 °C in a high vacuum oven, **18** was collected as a light green powder (0.48 g, 1.2 mmol, 61%).

^{1}H NMR (500 MHz, $CDCl_3$) δ 8.07 – 7.92 (m, 2H), 7.19 (td, *J* = 7.6, 1.3 Hz, 1H), 7.09 (dd, *J* = 7.3, 1.3 Hz, 1H), 6.94 – 6.84 (m, 2H), 6.74 (d, *J* = 8.8 Hz, 1H), 6.58 (d, *J* = 7.8 Hz, 1H), 5.86 (d, *J* = 10.4 Hz, 1H), 5.77 (ddt, *J* = 16.9, 10.2, 6.5 Hz, 1H), 5.04 – 4.92 (m, 2H), 3.27 – 3.08 (m, 2H), 2.16 – 1.98 (m, 2H), 1.82 – 1.58 (m, 2H), 1.28 (s, 3H), 1.18 (s, 3H). ^{13}C NMR (126 MHz, $CDCl_3$) δ 159.80, 147.21, 141.04, 138.01, 136.07, 128.26, 127.88, 126.02, 122.84, 122.16, 121.83, 119.50, 118.62, 115.69, 115.22, 106.87, 106.84, 52.80, 43.36, 31.48, 28.21, 26.16, 20.00. HRMS-ESI (m/z): [M+H] calcd for $C_{23}H_{25}N_2O3$, 377.1865; found, 377.1862.

Synthesis of 3',3'-dimethyl-6-nitro-1'-(5-(triethoxysilyl)pentyl)spiro[chromene-2,2'-indolin]-5'-yl methacrylate (**6**)

Following the general procedure for the hydrosilylation, **18** (49 mg, 0.13 mmol, 1 equiv) was dissolved in 5 ml of dry toluene and 20 µL of Pt catalyst solution was added. After triethoxysilane (72 µL, 0.39 mmol, 3 equiv) was added, the mixture was further stirred for 2 h. Evaporation of all the volatile parts from the solution yielded a yellow powder.

^{1}H NMR (500 MHz, $CDCl_3$): δ 8.04 – 7.96 (m, 2H), 7.21 – 7.15 (m, 1H), 7.08 (dt, *J* = 7.2, 1.6 Hz, 1H), 6.94 – 6.82 (m, 2H), 6.77 – 6.71 (m, 1H), 6.57 (dd, *J* = 7.7, 3.2 Hz, 1H), 5.89 – 5.81 (m, 1H), 3.94 – 3.71 (m, 6H), 3.26 – 3.05 (m, 2H), 2.34 (d, *J* = 19.7 Hz, 1H), 1.68 – 1.58 (m, 1H), 1.29 – 1.14 (m, 19H), 0.71 – 0.47 (m, 2H). ^{13}C NMR (126 MHz, $CDCl_3$): δ 159.84, 147.28, 140.91, 136.01, 129.16, 128.17, 127.85, 125.98, 122.81, 122.18, 121.77, 119.35, 118.59, 115.66, 106.89, 59.29, 58.45, 52.76, 43.86, 32.24, 30.88, 28.75, 26.10, 22.77, 19.96, 18.45, 18.26, 18.13, 10.53. HRMS-ESI (m/z): [M+H] calcd for $C_{29}H_{41}N_2O_6Si$, 541.2734; found, 541.2739.

2.3 Di-functionalized mechanically active SP (MA-SP-MA)

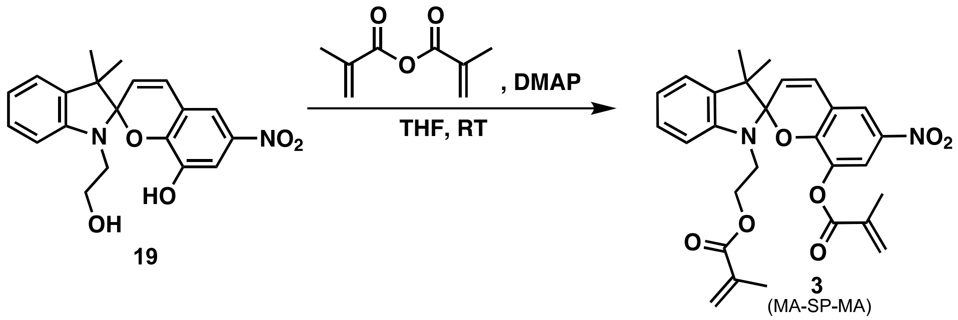

Scheme 8. Synthesis of di-functionalized SP for cross-linkers

Synthesis of 1'-(2-hydroxyethyl)-3',3'-dimethyl-6-nitrospiro[chromene-2,2'-indolin]-8-ol (**19**)

Dihydroxy SPs were synthesized following a similar procedure by Gossweiler et al.[2]

^{1}H NMR (500 MHz, d_6-DMSO): δ 8.55 (d, *J* = 2.7 Hz, 1H), 8.52 (d, *J* = 16.6 Hz, 1H), 8.00 – 7.93 (m, 2H), 7.93 – 7.87 (m, 2H), 7.67 – 7.61 (m, 2H), 4.82 (t, *J* = 5.0 Hz, 2H), 3.91 (t, *J* = 5.0 Hz, 2H), 1.80 (s, 6H). ^{13}C NMR (126 MHz, d_6-DMSO): δ 183.31, 153.66, 146.48, 146.33, 143.73, 140.93, 139.71, 129.50, 128.99, 122.96, 120.96, 115.96, 115.78, 115.02, 112.24, 58.47, 52.40, 49.67, 26.18. HRMS-ESI (m/z): [M+H] calcd for $C_{20}H_{21}N_2O_5$, 369.1450; found, 369.1439.

Synthesis of 2-(8-(methacryloyloxy)-3',3'-dimethyl-6-nitrospiro[chromene-2,2'-indolin]-1'-yl)ethyl methacrylate (**3**)

To a solution of **19** (600 mg, 1.6 mmol, 1 equiv) and 4-dimethylaminopyridine (360 mg, 2.9 mmol, 1.8 equiv) in 24ml of THF was added methacrylic anhydride (620 μL, 4.2 mmol, 2.6 equiv) dropwise. After stirring for 5 hours at room temperature, the reaction was quenched with 0.5 ml of methanol. The crude mixture was passed through a plug of basic alumina and eluted from the column with dichloromethane. The solution was concentrated and yielded a viscous purple oil. The oil was further dried under high vacuum and subsequently recrystallized from boiling hexane to yield as a yellow crystal (330 mg, 0.65 mmol, 40%).

TLC (Methylene chloride) R_f: 0.70 (UV)

^{1}H NMR (500 MHz, $CDCl_3$): δ 7.95 (d, *J* = 2.7 Hz, 1H), 7.90 (t, *J* = 2.8 Hz, 1H), 7.12 (td, *J* = 7.6, 1.3 Hz, 1H), 7.01 (d, *J* = 7.1 Hz, 1H), 6.96 (d, *J* = 10.4 Hz, 1H), 6.83 (t, *J* = 7.3 Hz, 1H), 6.62 (d, *J* = 7.7 Hz, 1H), 6.07 (d, *J* = 5.6 Hz, 1H), 5.94 (d, *J* = 10.4 Hz, 1H), 5.87 (d, *J* = 5.5 Hz, 1H), 5.58 – 5.50 (m, 1H), 5.38 (t, *J* = 1.7 Hz, 1H), 4.26 (t, *J* = 6.1 Hz, 2H), 3.36 (t, *J* = 6.1 Hz, 2H), 1.91 (d, *J* = 5.5 Hz, 3H), 1.61 (d, *J* = 5.3 Hz, 3H), 1.21 (dd, *J* = 34.4, 5.4 Hz, 6H). ^{13}C NMR

(126 MHz, $CDCl_3$): δ 167.31, 164.91, 150.92, 146.64, 140.62, 138.36, 136.46, 136.07, 134.91, 128.50, 127.76, 127.29, 125.73, 121.65, 121.58, 120.21, 119.99, 119.45, 119.38, 107.47, 107.19, 62.89, 52.32, 42.69, 25.90, 19.60, 18.42, 17.82. HRMS-ESI (m/z): [M+H] calcd for $C_{28}H_{29}N_2O_7$, 505.1975; found, 505.1972.

2.4 NMR spectra

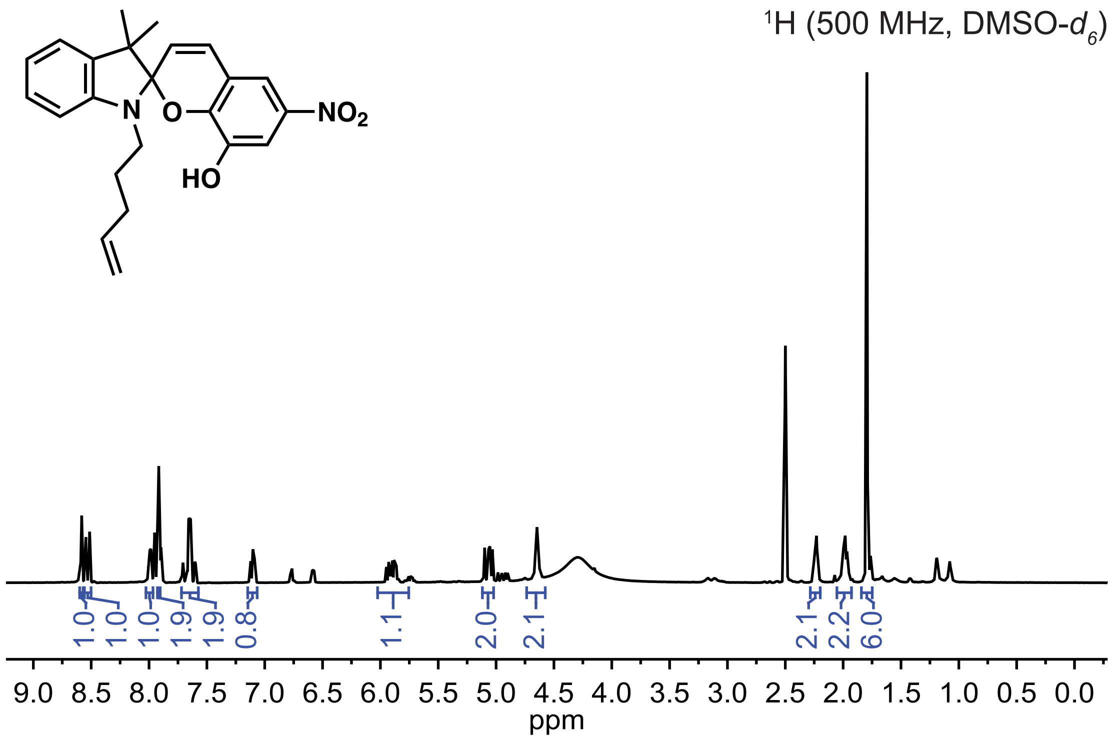

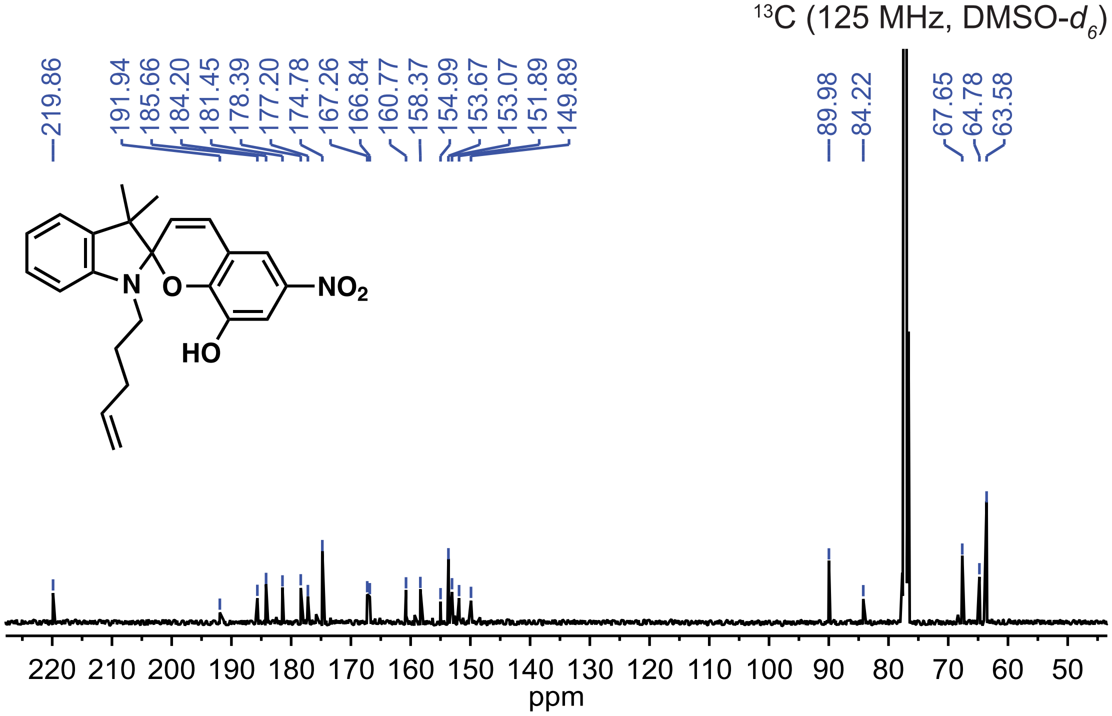

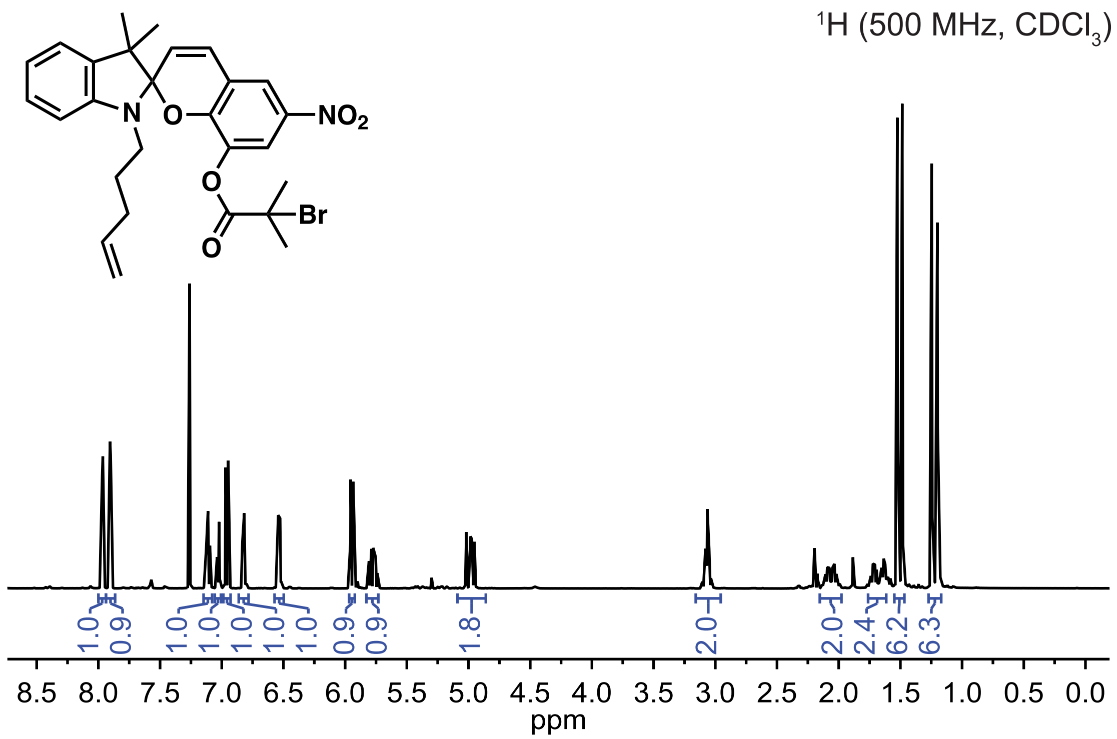

1H (500 MHz, CDCl3)
NO2
Br
N
O
1.0
0.9
1.0
1.0
1.0
1.0
1.0
0.9
0.9
1.8
2.0
2.0
2.4
6.2
6.3
8.5 8.0 7.5 7.0 6.5 6.0 5.5 5.0 4.5 4.0 3.5 3.0 2.5 2.0 1.5 1.0 0.5 0.0
ppm

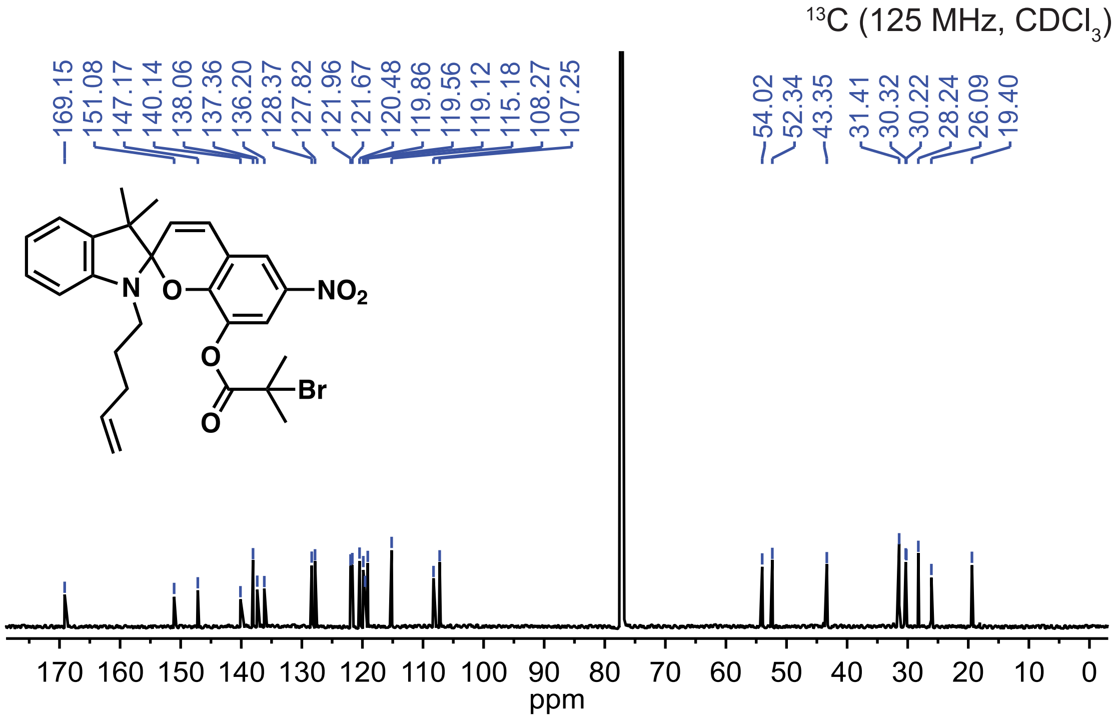

13C (125 MHz, CDCl3)
169.15
151.08
147.17
140.14
138.06
137.36
136.20
128.37
127.82
121.96
121.67
120.48
119.86
119.56
119.12
115.18
108.27
107.25
54.02
52.34
43.35
31.41
30.32
30.22
28.24
26.09
19.40
NO2
Br
N
O
170 160 150 140 130 120 110 100 90 80 70 60 50 40 30 20 10 0
ppm

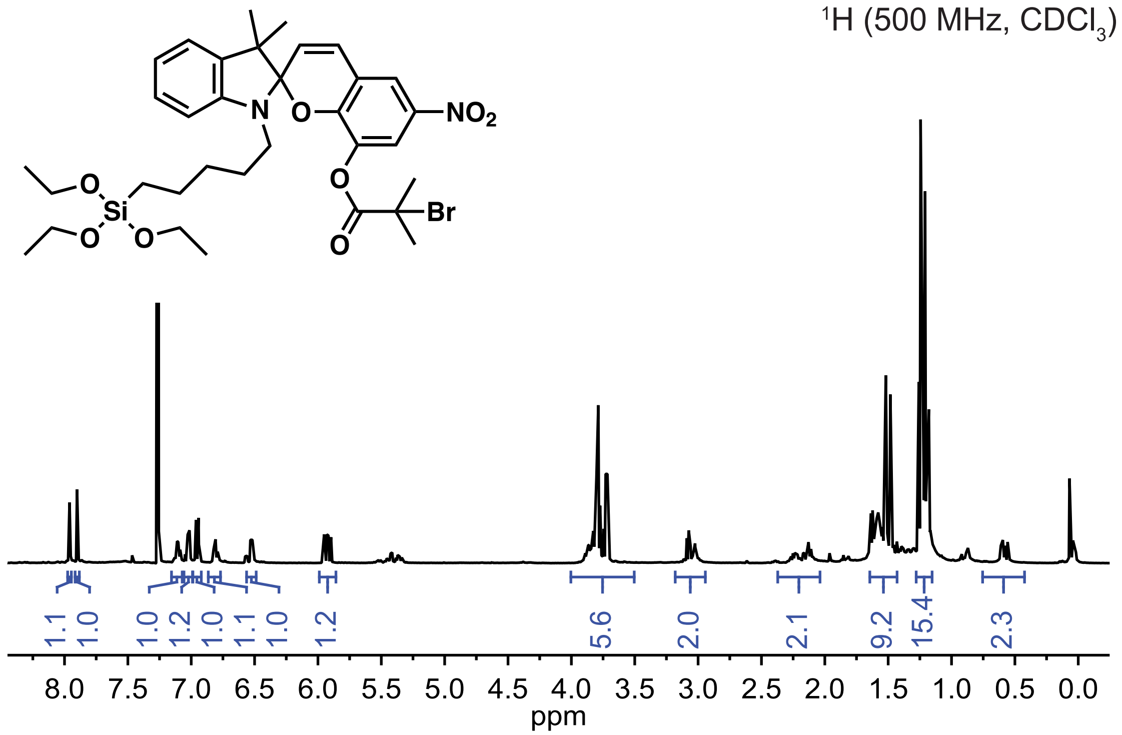
1H (500 MHz, CDCl3)
NO2
N
O
O
Si
O
O
O
O
Br
O
1.1
1.0
1.0
1.2
1.0
1.1
1.0
1.2
5.6
2.0
2.1
9.2
15.4
2.3
8.0 7.5 7.0 6.5 6.0 5.5 5.0 4.5 4.0 3.5 3.0 2.5 2.0 1.5 1.0 0.5 0.0
ppm

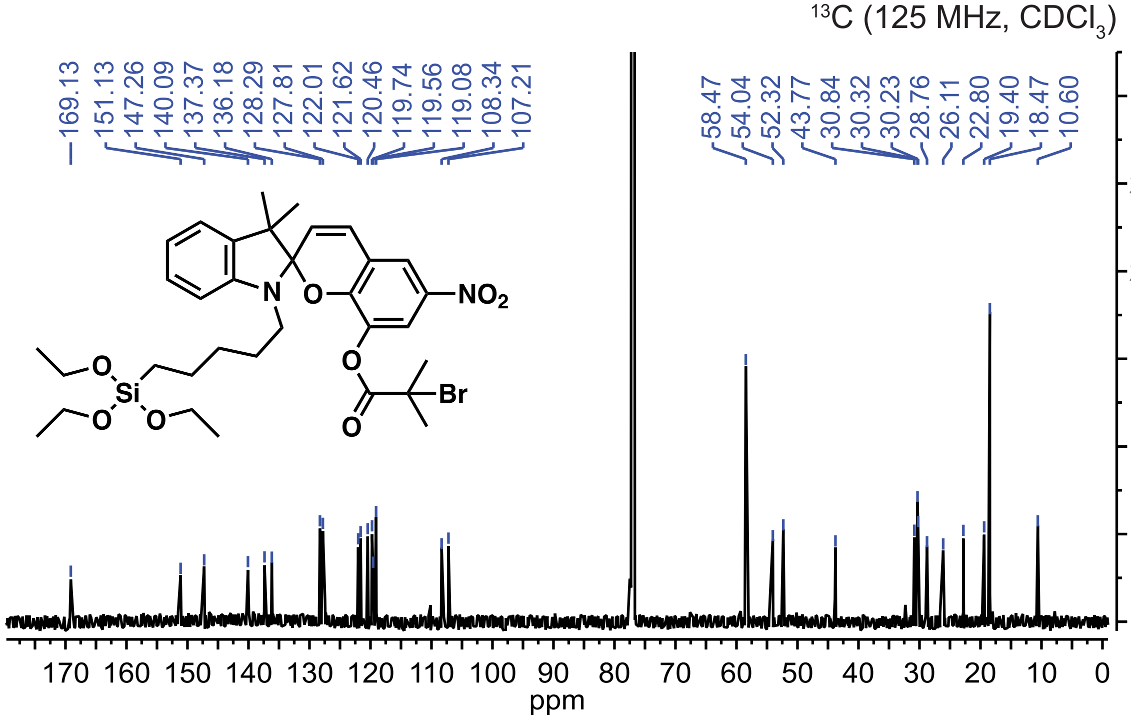
13C (125 MHz, CDCl3)
169.13
151.13
147.26
140.09
137.37
136.18
128.29
127.81
122.01
121.62
120.46
119.74
119.56
119.08
108.34
107.21
58.47
54.04
52.32
43.77
30.84
30.32
30.23
28.76
26.11
22.80
19.40
18.47
10.60
NO2
N
O
O
Si
O
O
O
O
Br
O
170 160 150 140 130 120 110 100 90 80 70 60 50 40 30 20 10 0
ppm

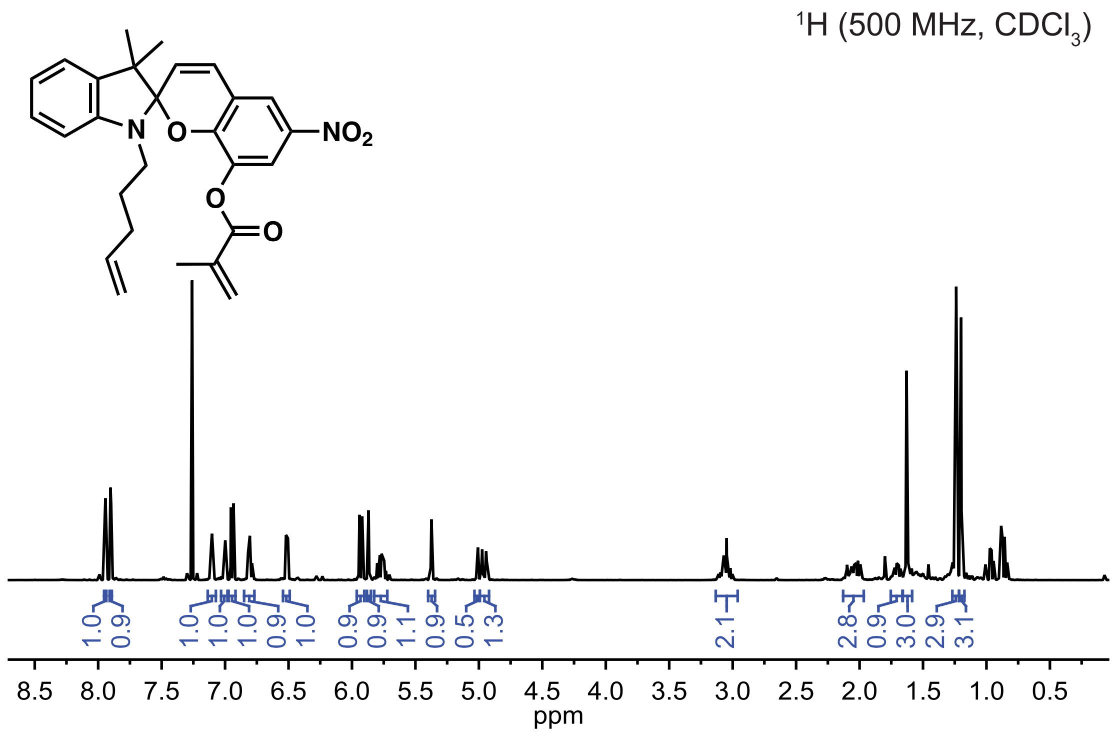
1H (500 MHz, CDCl3)
NO2
N
O
O
O
1.0 0.9 1.0 1.0 1.0 0.9 1.0 0.9 0.9 1.1 0.9 0.5 1.3 2.1 2.8 0.9 3.0 2.9 3.1
8.5 8.0 7.5 7.0 6.5 6.0 5.5 5.0 4.5 4.0 3.5 3.0 2.5 2.0 1.5 1.0 0.5
ppm

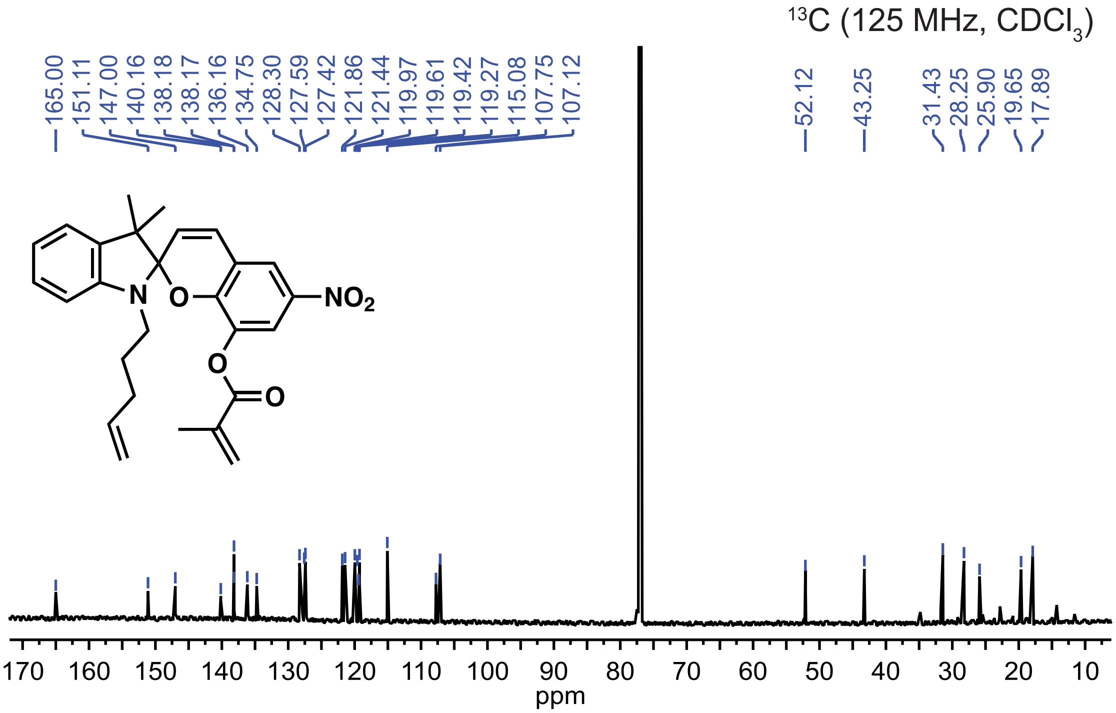
13C (125 MHz, CDCl3)
165.00 151.11 147.00 140.16 138.18 138.17 136.16 134.75 128.30 127.59 127.42 121.86 121.44 119.97 119.61 119.42 119.27 115.08 107.75 107.12
52.12
43.25
31.43 28.25 25.90 19.65 17.89
NO2
N
O
O
O
170 160 150 140 130 120 110 100 90 80 70 60 50 40 30 20 10
ppm

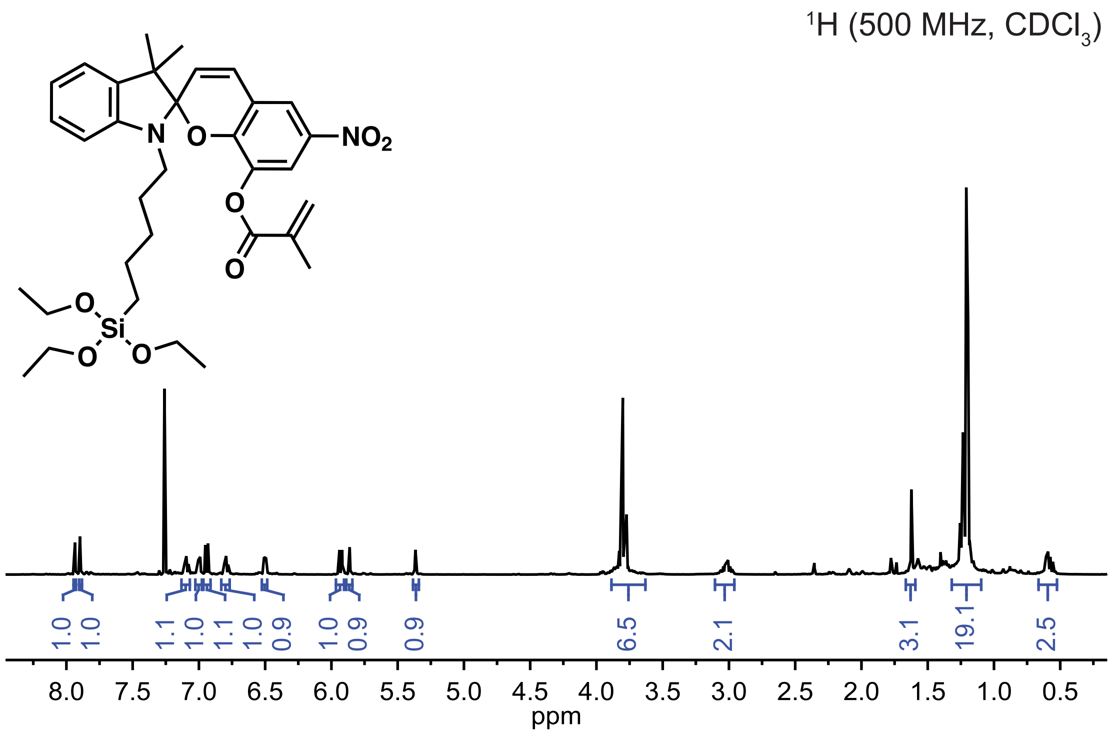

1H (500 MHz, CDCl3)
NO2
N
O
O
O
Si
1.0 1.0 1.1 1.0 1.1 1.0 0.9 1.0 0.9 0.9 6.5 2.1 3.1 19.1 2.5
8.0 7.5 7.0 6.5 6.0 5.5 5.0 4.5 4.0 3.5 3.0 2.5 2.0 1.5 1.0 0.5
ppm

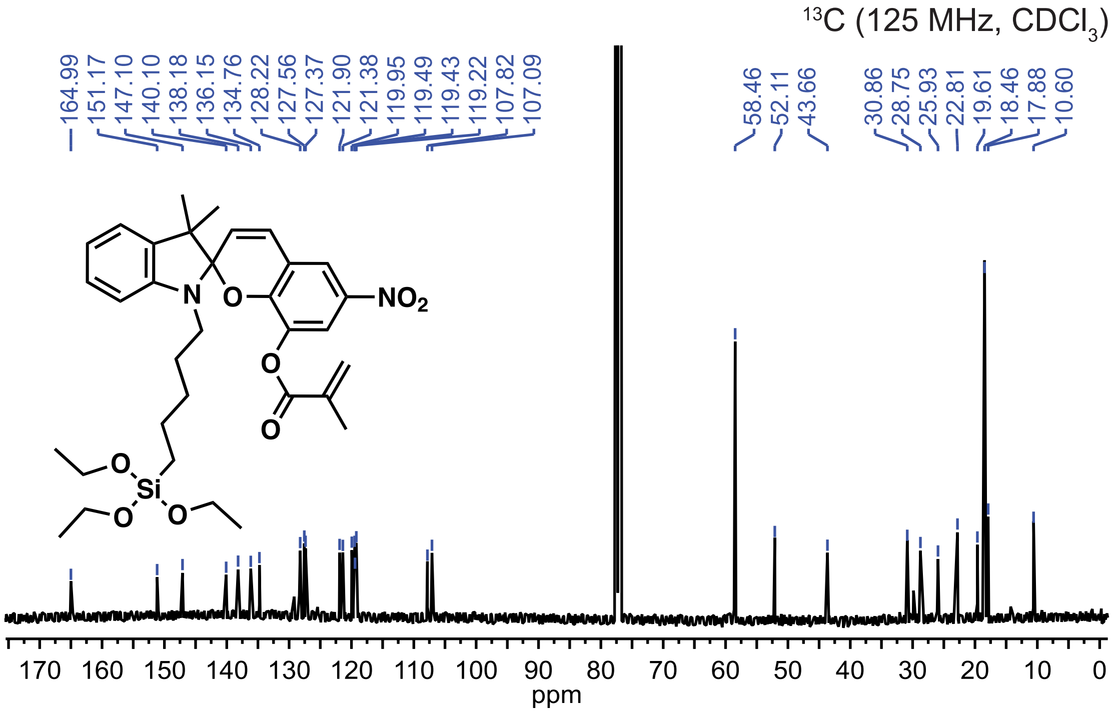

13C (125 MHz, CDCl3)
164.99 151.17 147.10 140.10 138.18 136.15 134.76 128.22 127.56 127.37 121.90 121.38 119.95 119.49 119.43 119.22 107.82 107.09
58.46 52.11 43.66
30.86 28.75 25.93 22.81 19.61 18.46 17.88 10.60
NO2
N
O
O
O
Si
170 160 150 140 130 120 110 100 90 80 70 60 50 40 30 20 10 0
ppm

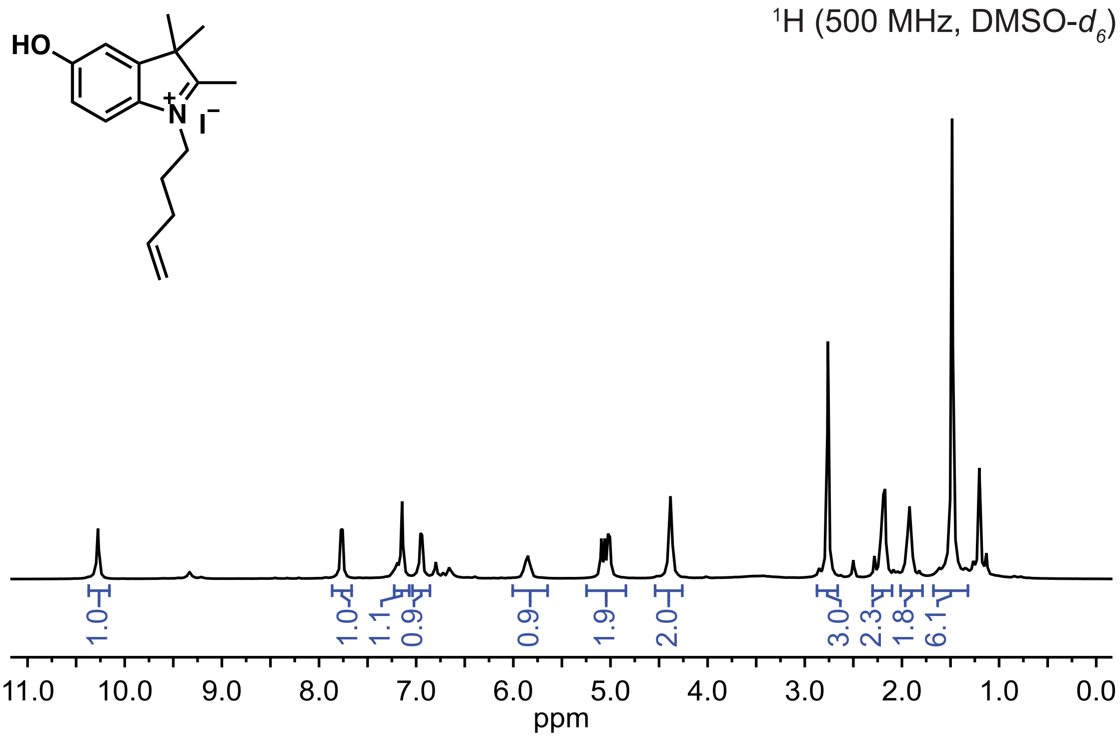

^{1}H (500 MHz, DMSO-d_6)
HO
N
I
1.0
1.0
1.1
0.9
0.9
1.9
2.0
3.0
2.3
1.8
6.1
11.0 10.0 9.0 8.0 7.0 6.0 5.0 4.0 3.0 2.0 1.0 0.0
ppm

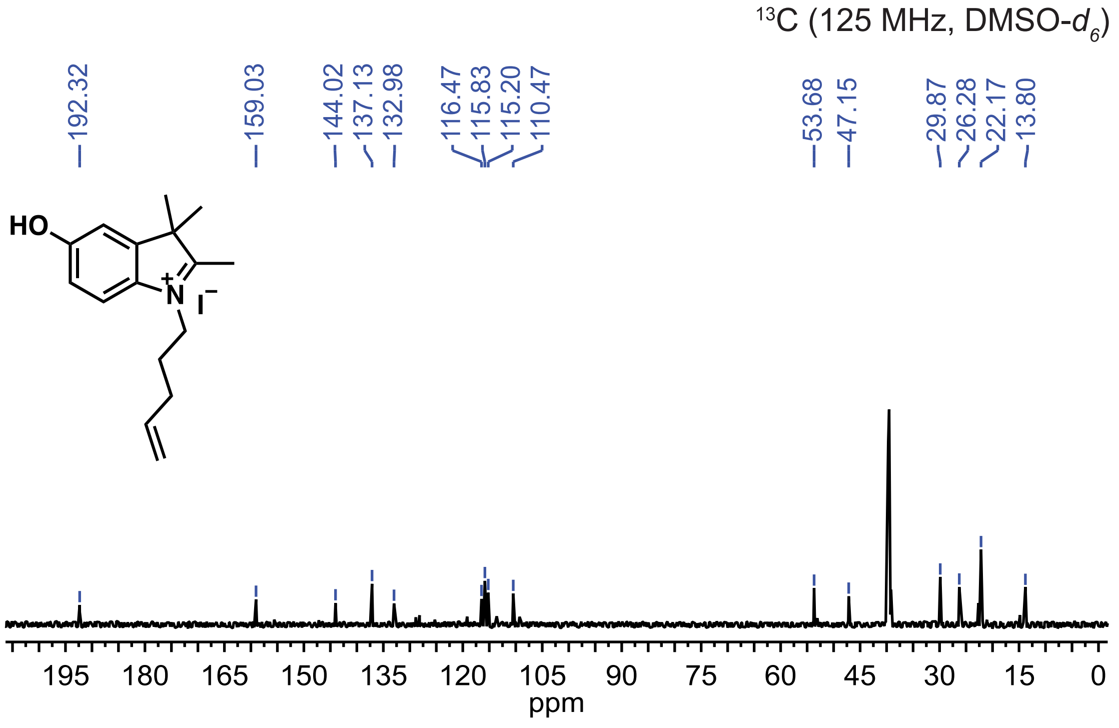

^{13}C (125 MHz, DMSO-d_6)
192.32
159.03
144.02
137.13
132.98
116.47
115.83
115.20
110.47
53.68
47.15
29.87
26.28
22.17
13.80
HO
N
I
195 180 165 150 135 120 105 90 75 60 45 30 15 0
ppm

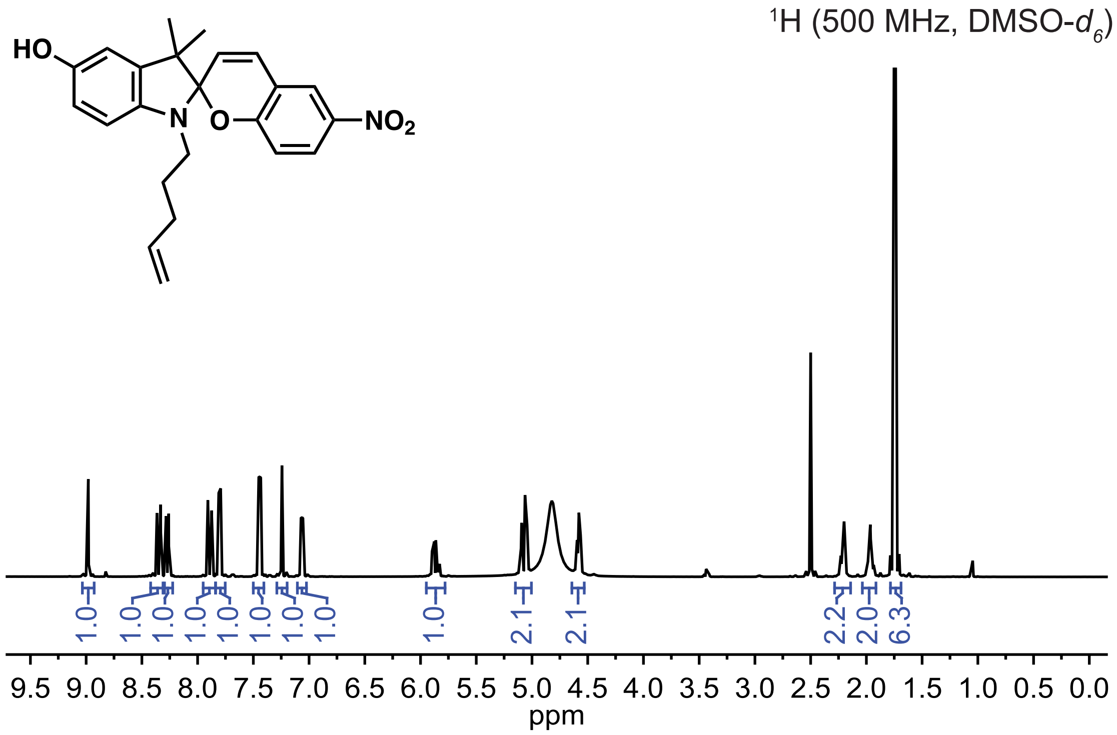

1H (500 MHz, DMSO-d6)
HO
N
O
NO2
1.0
1.0
1.0
1.0
1.0
1.0
1.0
1.0
1.0
2.1
2.1
2.2
2.0
6.3
9.5 9.0 8.5 8.0 7.5 7.0 6.5 6.0 5.5 5.0 4.5 4.0 3.5 3.0 2.5 2.0 1.5 1.0 0.5 0.0
ppm

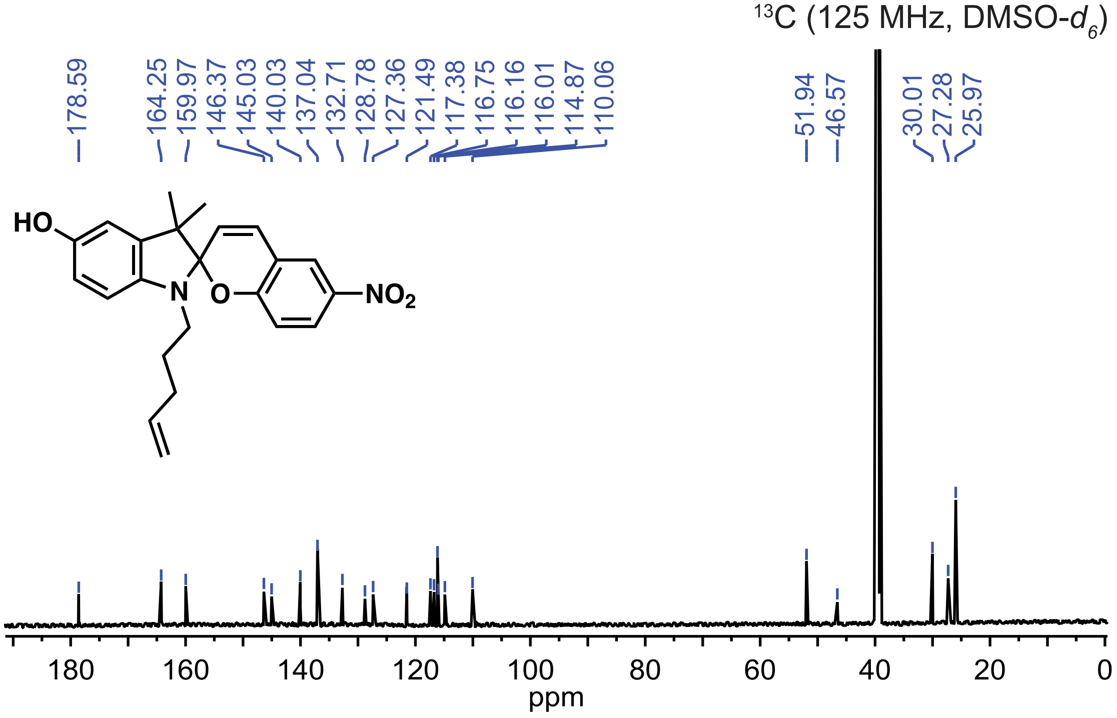

13C (125 MHz, DMSO-d6)
178.59
164.25
159.97
146.37
145.03
140.03
137.04
132.71
128.78
127.36
121.49
117.38
116.75
116.16
116.01
114.87
110.06
51.94
46.57
30.01
27.28
25.97
HO
N
O
NO2
180 160 140 120 100 80 60 40 20 0
ppm

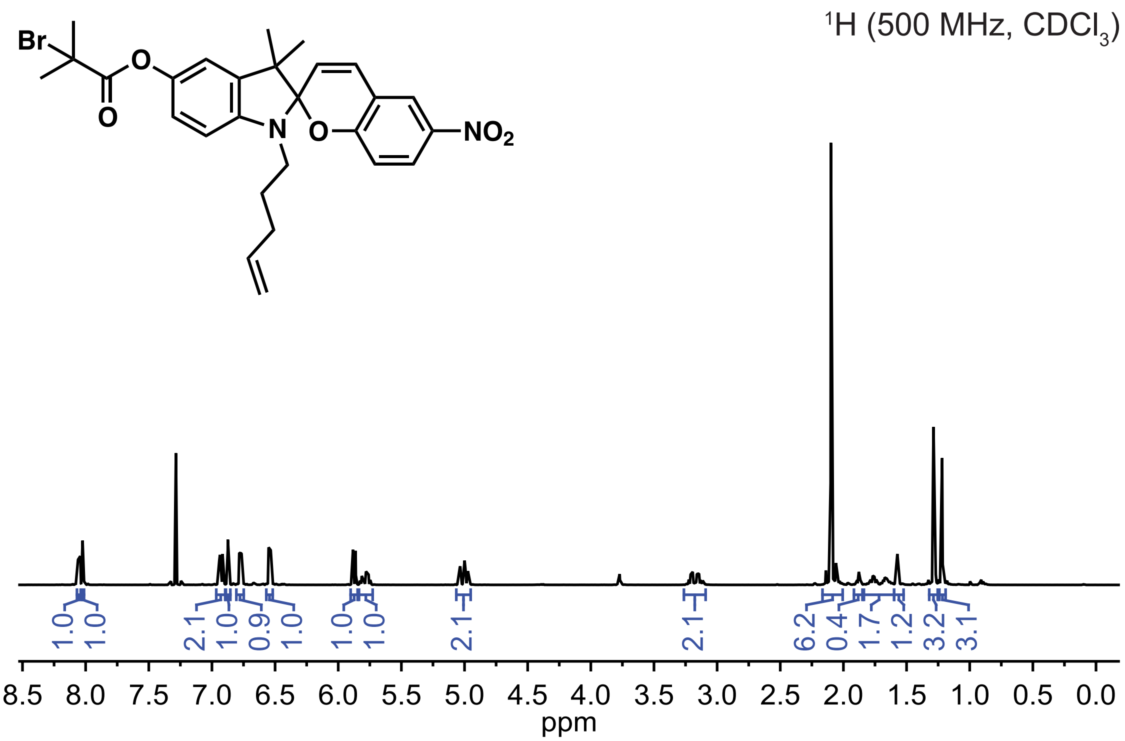

Br
O
O
N
O
NO2
1H (500 MHz, CDCl3)
1.0
1.0
2.1
1.0
0.9
1.0
1.0
1.0
2.1
2.1
6.2
0.4
1.7
1.2
3.2
3.1
8.5 8.0 7.5 7.0 6.5 6.0 5.5 5.0 4.5 4.0 3.5 3.0 2.5 2.0 1.5 1.0 0.5 0.0
ppm

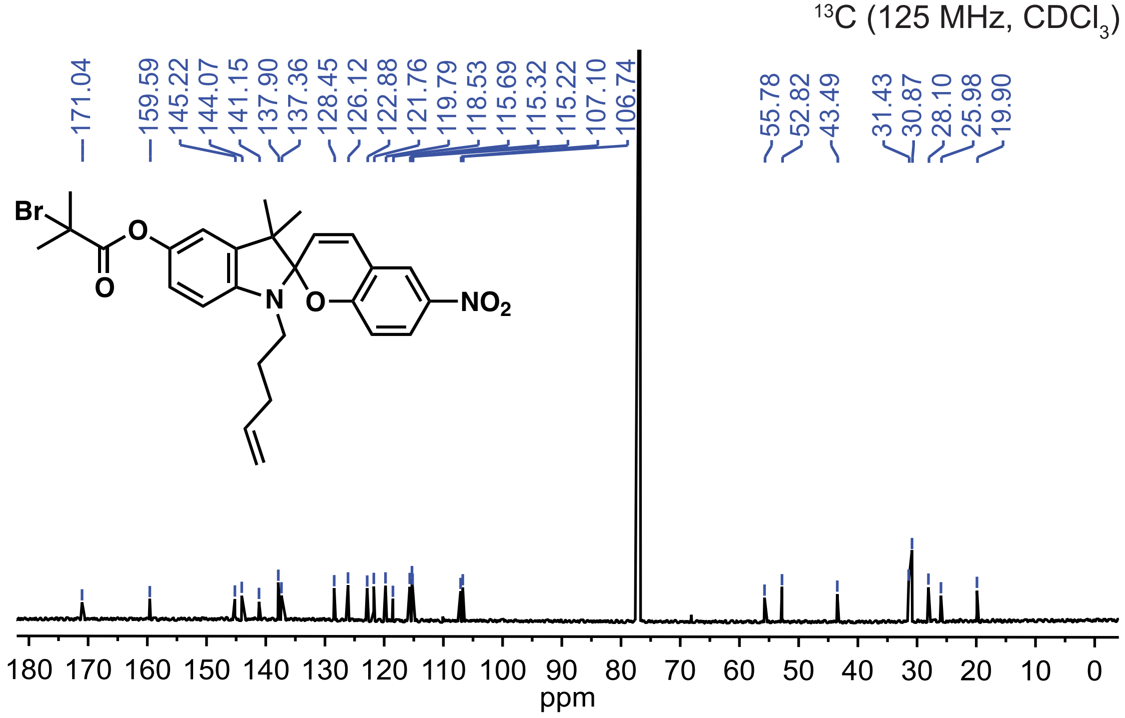

13C (125 MHz, CDCl3)
171.04
159.59
145.22
144.07
141.15
137.90
137.36
128.45
126.12
122.88
121.76
119.79
118.53
115.69
115.32
115.22
107.10
106.74
55.78
52.82
43.49
31.43
30.87
28.10
25.98
19.90
Br
O
O
N
O
NO2
180 170 160 150 140 130 120 110 100 90 80 70 60 50 40 30 20 10 0
ppm

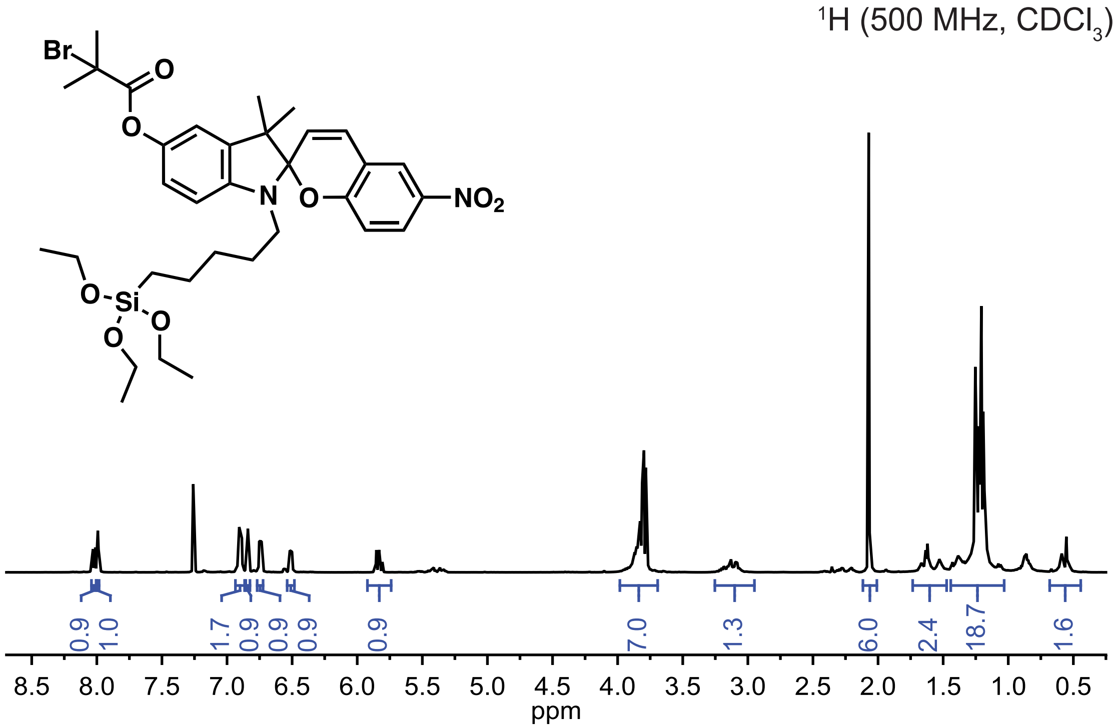

^{1}H (500 MHz, CDCl$_3$)
Br
O
O
N
O
NO$_2$
O
Si
O
O
0.9 1.0 1.7 0.9 0.9 0.9 0.9 7.0 1.3 6.0 2.4 18.7 1.6
8.5 8.0 7.5 7.0 6.5 6.0 5.5 5.0 4.5 4.0 3.5 3.0 2.5 2.0 1.5 1.0 0.5
ppm

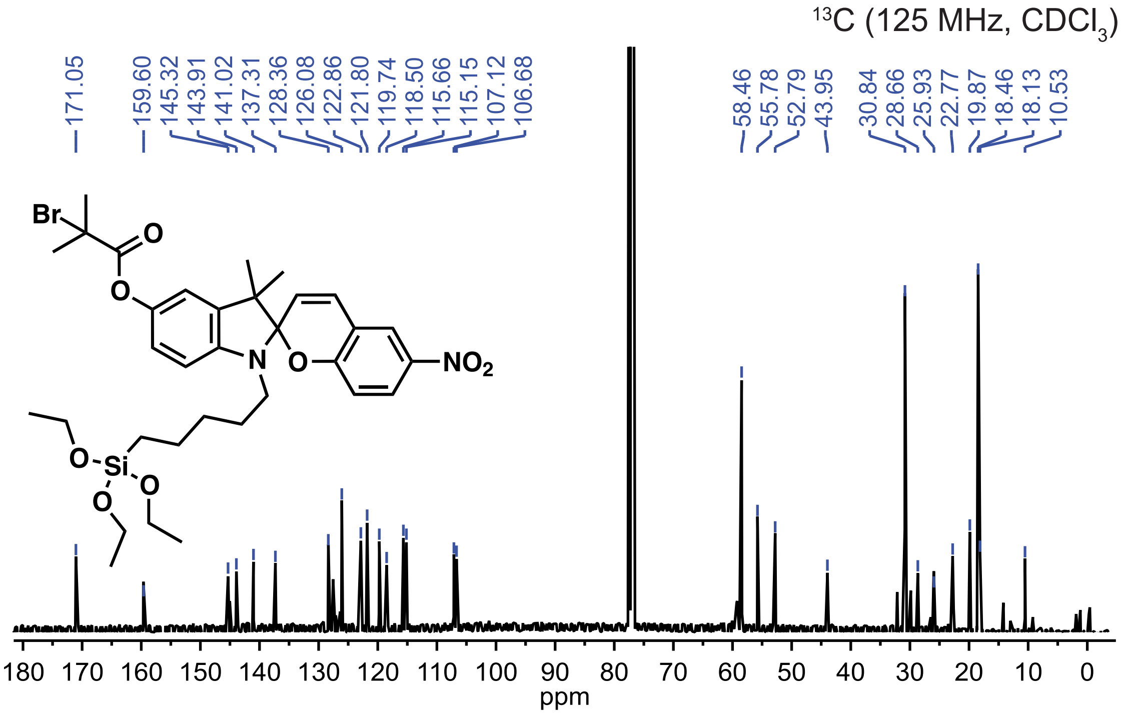

^{13}C (125 MHz, CDCl$_3$)
171.05
159.60 145.32 143.91 141.02 137.31 128.36 126.08 122.86 121.80 119.74 118.50 115.66 115.15 107.12 106.68
58.46 55.78 52.79 43.95
30.84 28.66 25.93 22.77 19.87 18.46 18.13 10.53
Br
O
O
N
O
NO$_2$
O
Si
O
O
180 170 160 150 140 130 120 110 100 90 80 70 60 50 40 30 20 10 0
ppm

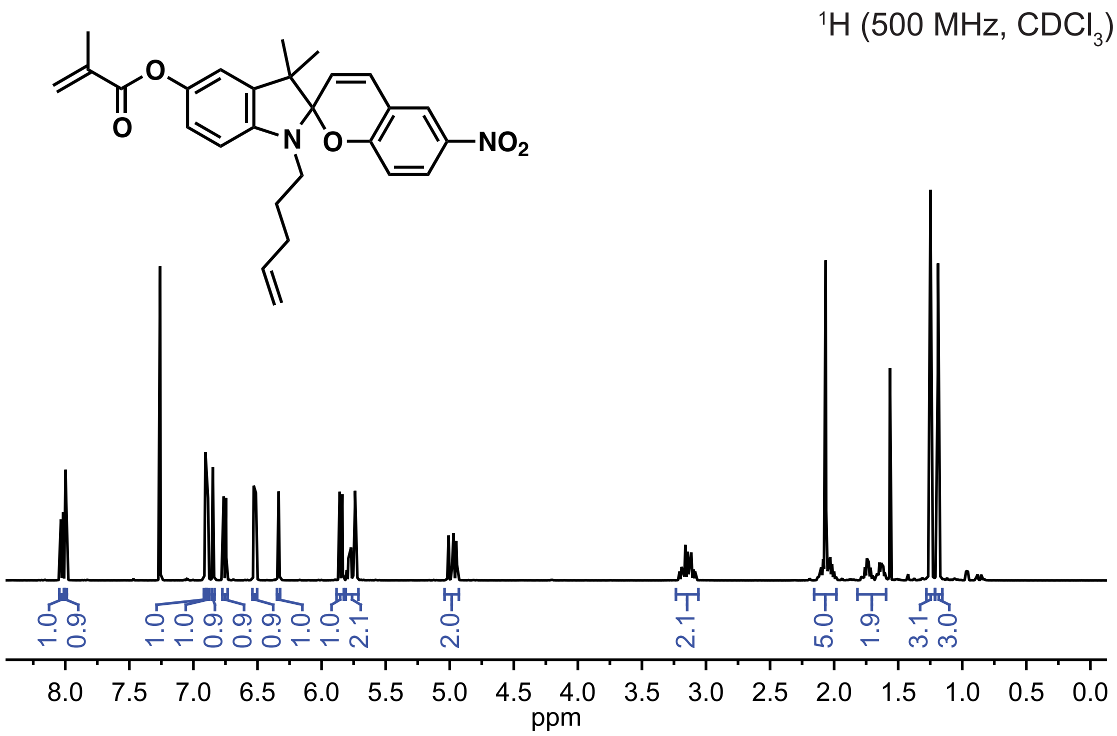

^{1}H (500 MHz, CDCl$_3$)
O
O
N
O
NO$_2$
1.0
0.9
1.0
1.0
0.9
0.9
0.9
1.0
1.0
2.1
2.0
2.1
5.0
1.9
3.1
3.0
8.0 7.5 7.0 6.5 6.0 5.5 5.0 4.5 4.0 3.5 3.0 2.5 2.0 1.5 1.0 0.5 0.0
ppm

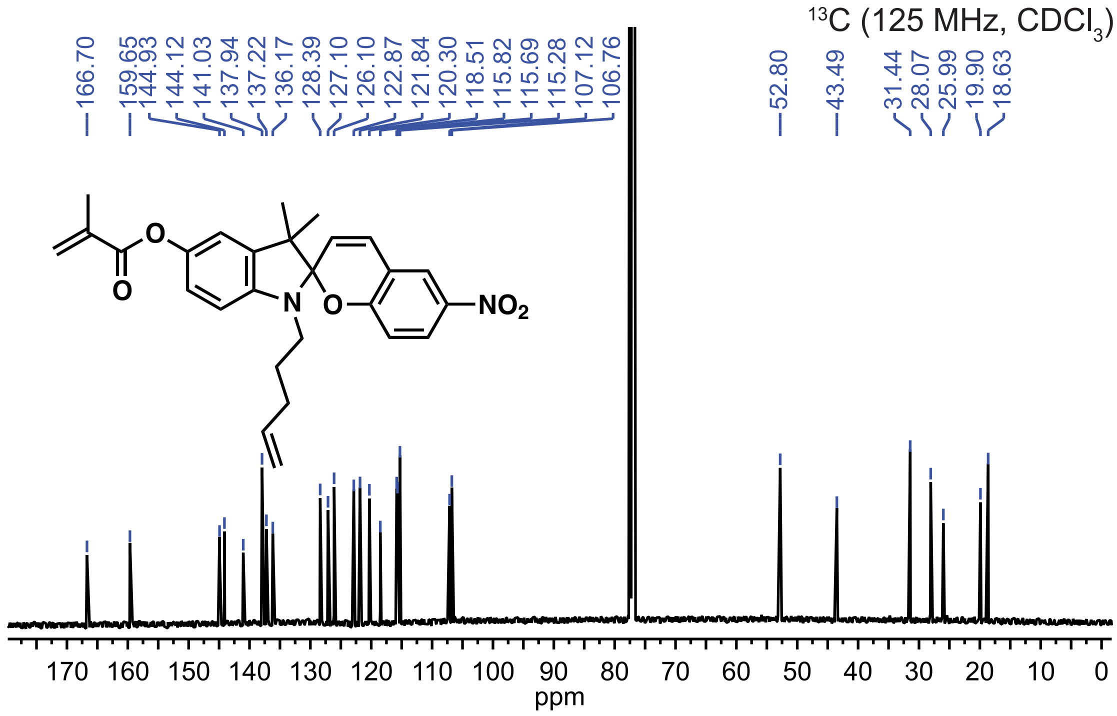

^{13}C (125 MHz, CDCl$_3$)
166.70
159.65
144.93
144.12
141.03
137.94
137.22
136.17
128.39
127.10
126.10
122.87
121.84
120.30
118.51
115.82
115.69
115.28
107.12
106.76
52.80
43.49
31.44
28.07
25.99
19.90
18.63
O
O
N
O
NO$_2$
170 160 150 140 130 120 110 100 90 80 70 60 50 40 30 20 10 0
ppm

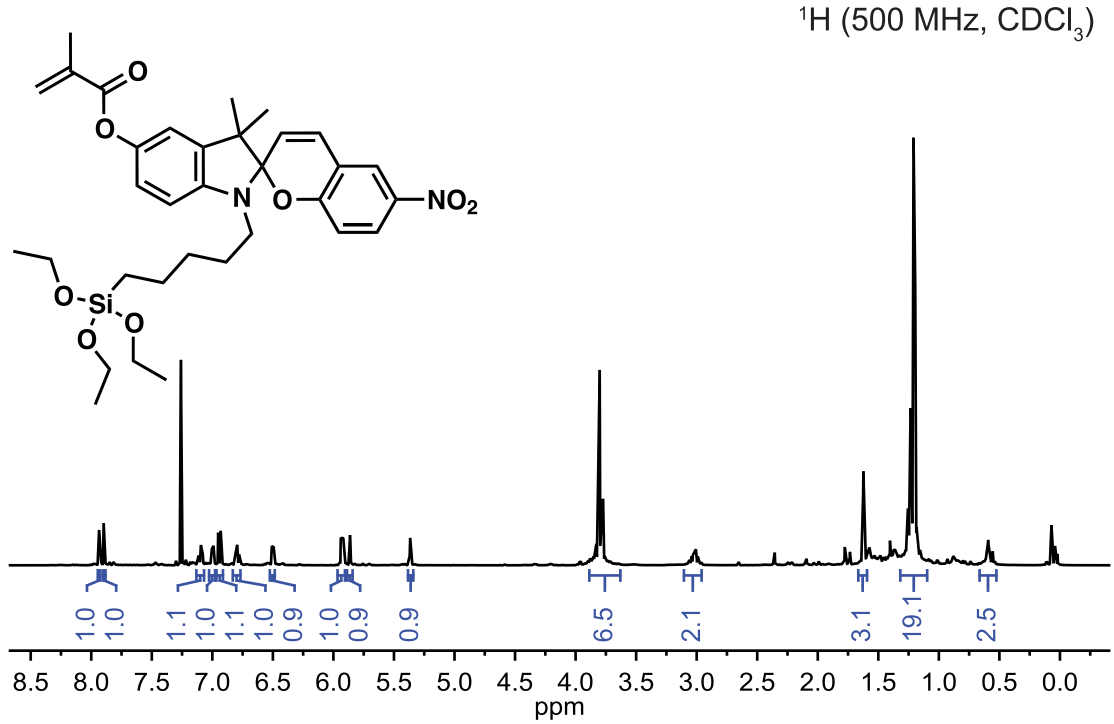

^{1}H (500 MHz, CDCl$_3$)
1.0
1.0
1.1
1.0
1.1
1.0
0.9
1.0
0.9
0.9
6.5
2.1
3.1
19.1
2.5
8.5 8.0 7.5 7.0 6.5 6.0 5.5 5.0 4.5 4.0 3.5 3.0 2.5 2.0 1.5 1.0 0.5 0.0
ppm

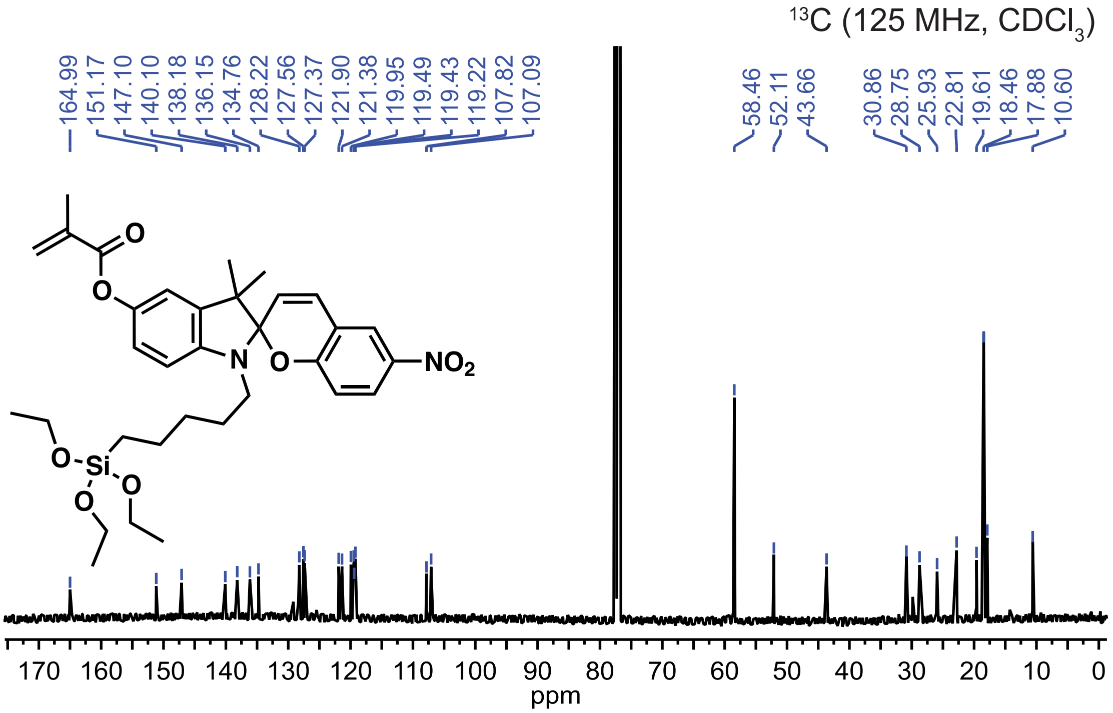

^{13}C (125 MHz, CDCl$_3$)
164.99
151.17
147.10
140.10
138.18
136.15
134.76
128.22
127.56
127.37
121.90
121.38
119.95
119.49
119.43
119.22
107.82
107.09
58.46
52.11
43.66
30.86
28.75
25.93
22.81
19.61
18.46
17.88
10.60
170 160 150 140 130 120 110 100 90 80 70 60 50 40 30 20 10 0
ppm

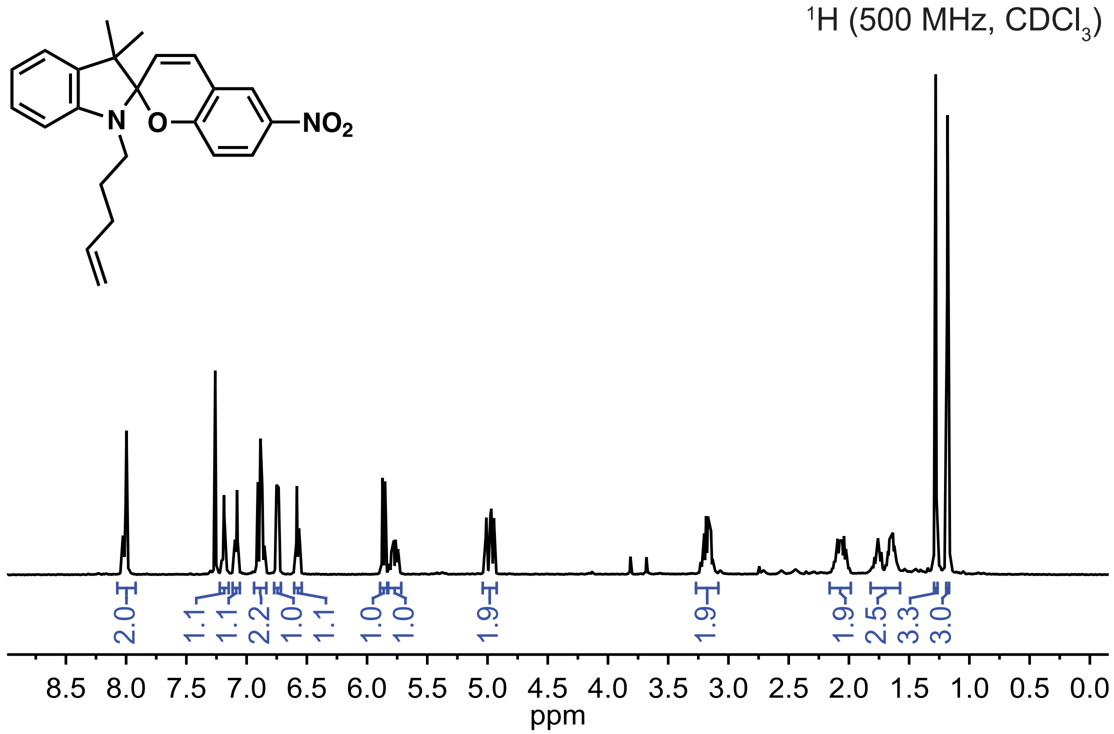

^{1}H (500 MHz, $CDCl_3$)
N
O
NO_2
2.0
1.1
1.1
2.2
1.0
1.1
1.0
1.0
1.9
1.9
1.9
2.5
3.3
3.0
8.5 8.0 7.5 7.0 6.5 6.0 5.5 5.0 4.5 4.0 3.5 3.0 2.5 2.0 1.5 1.0 0.5 0.0
ppm

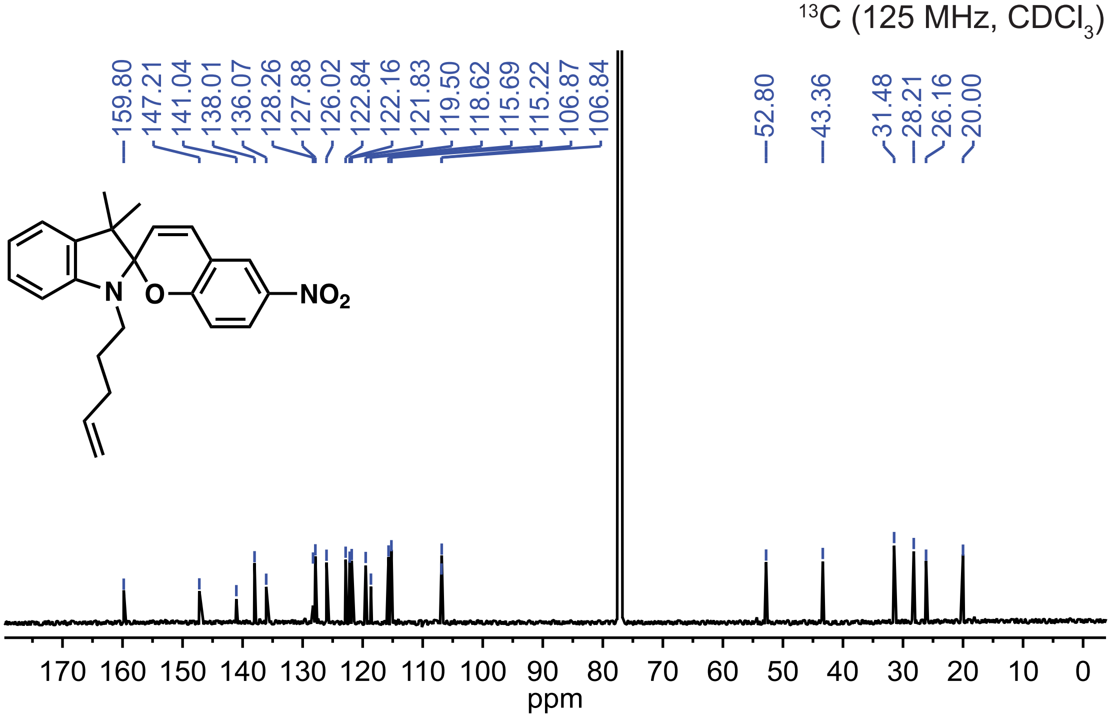

^{13}C (125 MHz, $CDCl_3$)
159.80
147.21
141.04
138.01
136.07
128.26
127.88
126.02
122.84
122.16
121.83
119.50
118.62
115.69
115.22
106.87
106.84
52.80
43.36
31.48
28.21
26.16
20.00
N
O
NO_2
170 160 150 140 130 120 110 100 90 80 70 60 50 40 30 20 10 0
ppm

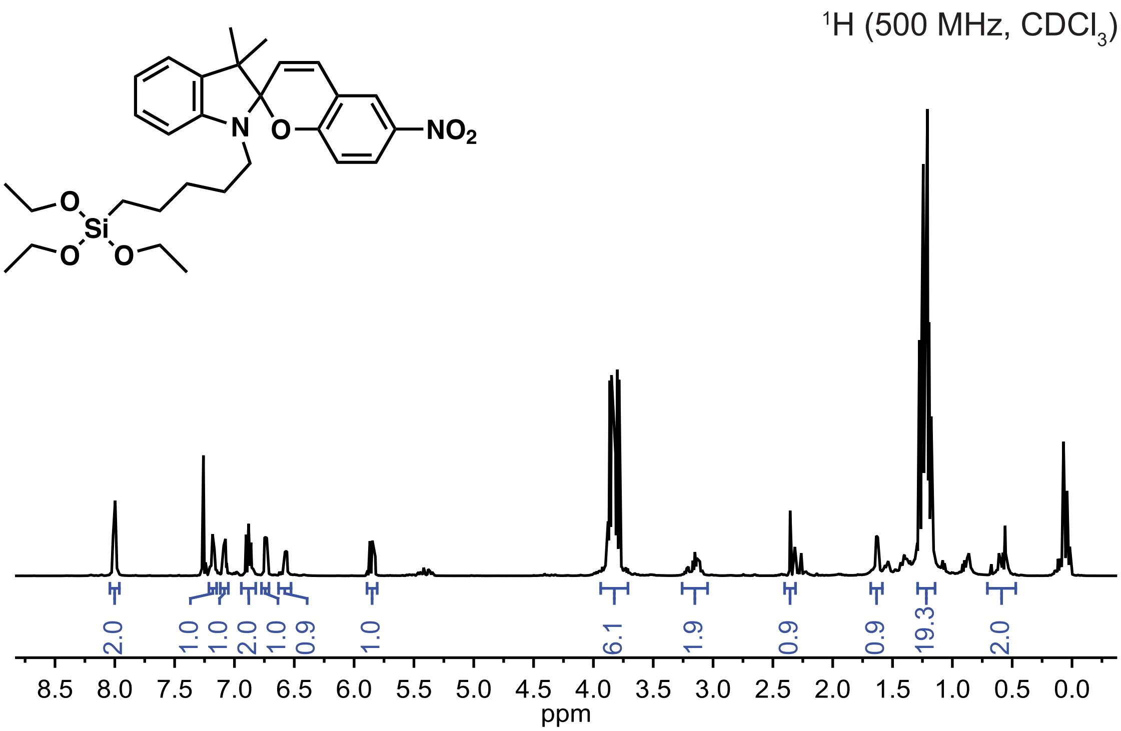
1H (500 MHz, CDCl3)
NO2
N
O
Si
2.0
1.0
1.0
2.0
1.0
0.9
1.0
6.1
1.9
0.9
0.9
19.3
2.0
8.5 8.0 7.5 7.0 6.5 6.0 5.5 5.0 4.5 4.0 3.5 3.0 2.5 2.0 1.5 1.0 0.5 0.0
ppm

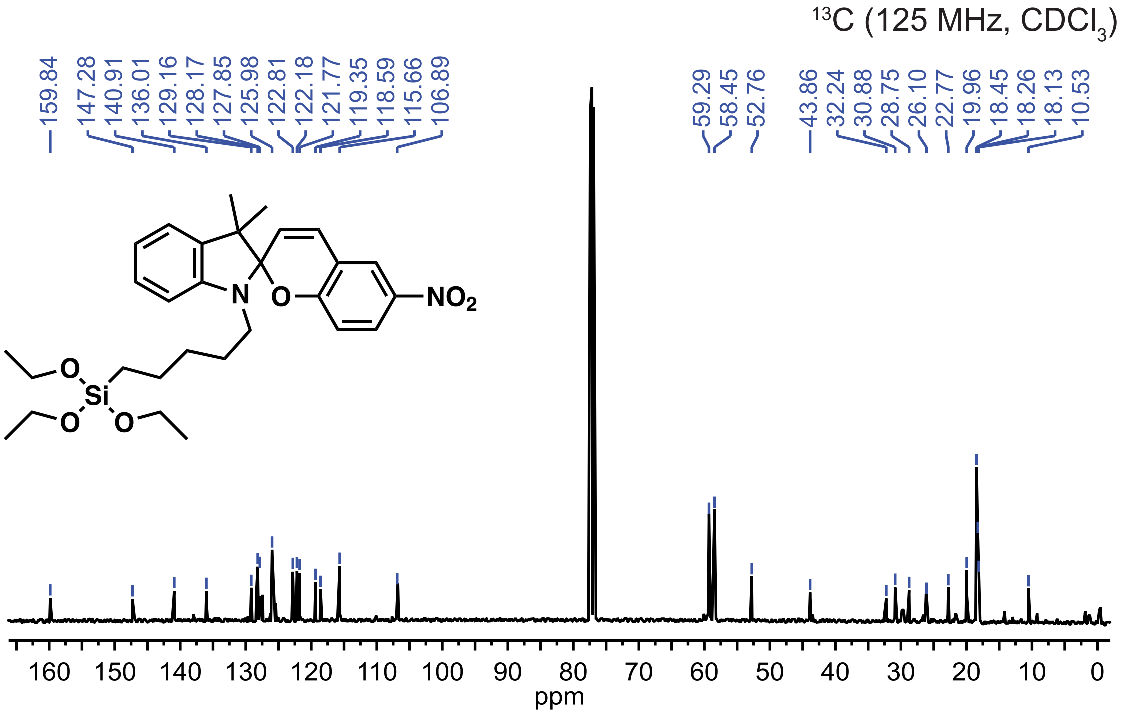
13C (125 MHz, CDCl3)
159.84
147.28
140.91
136.01
129.16
128.17
127.85
125.98
122.81
122.18
121.77
119.35
118.59
115.66
106.89
59.29
58.45
52.76
43.86
32.24
30.88
28.75
26.10
22.77
19.96
18.45
18.26
18.13
10.53
NO2
N
O
Si
160 150 140 130 120 110 100 90 80 70 60 50 40 30 20 10 0
ppm

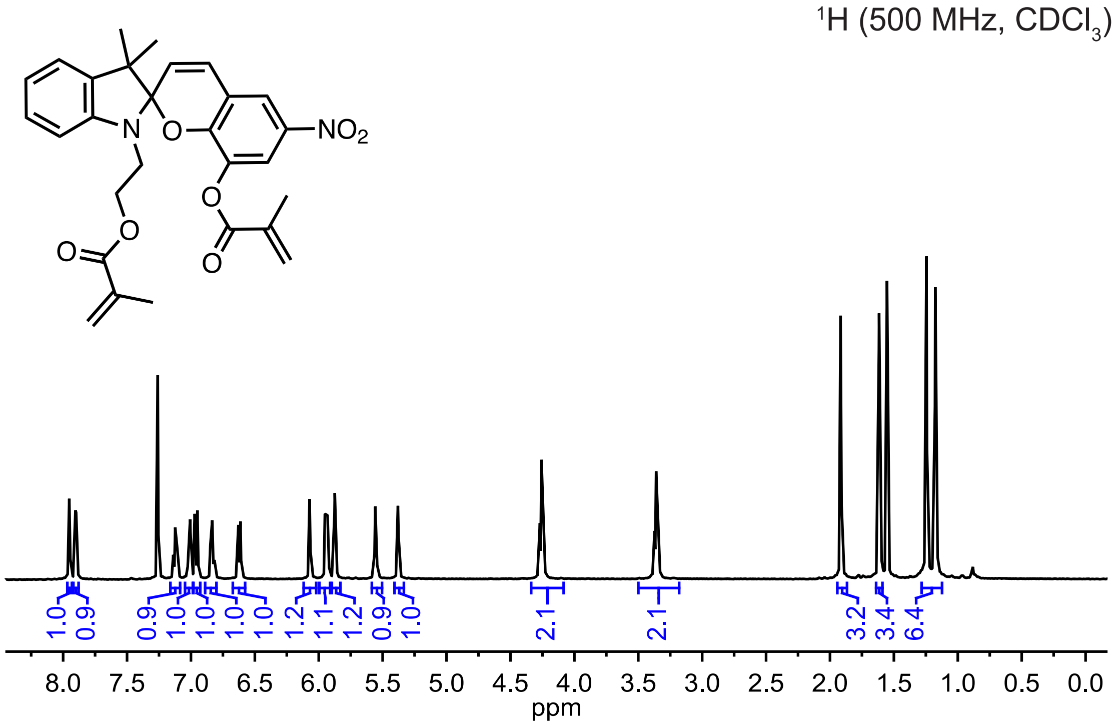

^{1}H (500 MHz, $CDCl_3$)
NO_2
N
O
1.0 0.9 0.9 1.0 1.0 1.0 1.0 1.2 1.1 1.2 0.9 1.0 2.1 2.1 3.2 3.4 6.4
8.0 7.5 7.0 6.5 6.0 5.5 5.0 4.5 4.0 3.5 3.0 2.5 2.0 1.5 1.0 0.5 0.0
ppm

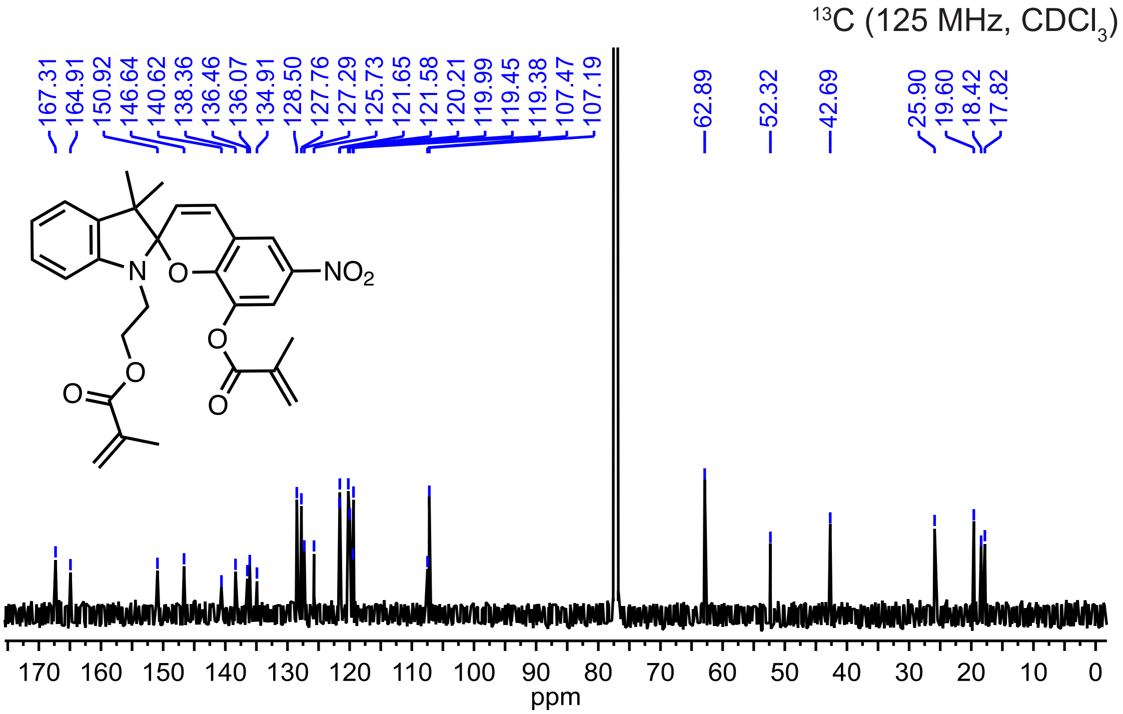

^{13}C (125 MHz, $CDCl_3$)
167.31 164.91 150.92 146.64 140.62 138.36 136.46 136.07 134.91 128.50 127.76 127.29 125.73 121.65 121.58 120.21 119.99 119.45 119.38 107.47 107.19
62.89 52.32 42.69 25.90 19.60 18.42 17.82
NO_2
N
O
170 160 150 140 130 120 110 100 90 80 70 60 50 40 30 20 10 0
ppm

3. Surface Functionalization of SiO_2 Particles

3.1 Instability of SP in ammonia solution

12 (5 mg, 0.013 mmol) was dissolved in 2ml of THF or toluene, followed by adding 0.1 ml of base. To confirm the photochromic behavior, UV light was exposed to the solution. After adding ammonia solution (30 wt% in water), SP loses photochromic behavior, indicating the degradation of SP structures. Base-catalyzed hydrolysis is the possible degradation mechanism that generates Fisher's base and salicylaldehyde from the merocyanine form.[3] With the addition of triethylamine, SP retains the reversible photochromic behavior in both polar (THF) and non-polar (toluene) solvent. Therefore, we chose triethylamine as a basic catalyst for surface functionalization of SiO_2 particles.

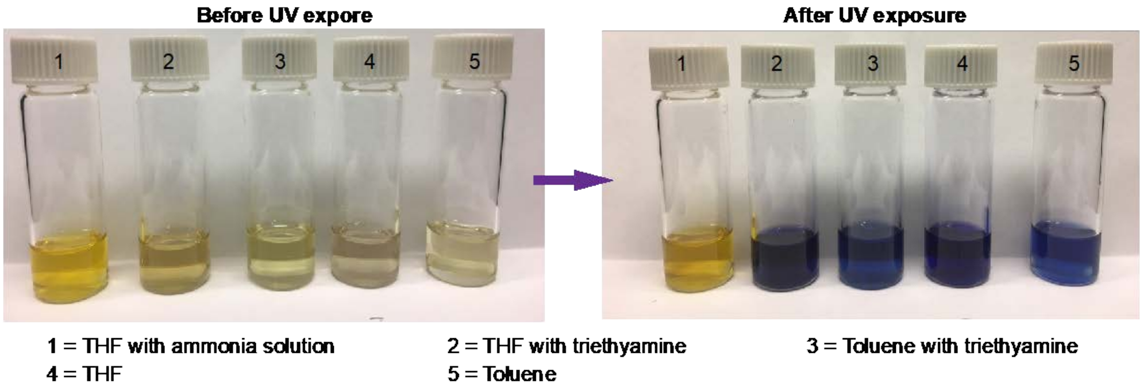

Figure S1. Stability of SP in various solvent conditions. All of the solutions except 1 (THF with ammonia solution) exhibits photochromic behavior, indicating degradation of SP structure in aqueous basic solution.

3.2 Optimized procedure for surface functionalization with SP

A representative procedure is utilized for surface functionalization of SiO_2 particles with **1** or **4**. 665 mg of SiO_2 solution (MEK-ST, 10–15nm, 30 wt% in Methyl ether ketone) was diluted with 5 ml of toluene and sonicated for 10 min to get a stable dispersion. Then, 0.4 ml of triethylamine was added to the SiO_2 dispersion. Triethoxysilane functionalized SP (0.27 mmol) was re-dissolved in 2 ml of toluene and transferred dropwise to the SiO_2 dispersion. After stirring overnight at room temperature, the functionalized SiO_2 particles were separated by centrifugation (IEC Multi centrifuge, Thermo Electron Corp., 5000 rpm, 5 min), followed by multiple washing (with toluene)/centrifugation cycles. Then, the powder was dried in vacuum and collected for further

polymerization. 1μm-sized SiO_2 particles were also functionalized in the same manner and characterized by fluorescence microscope (**Figure S2**).

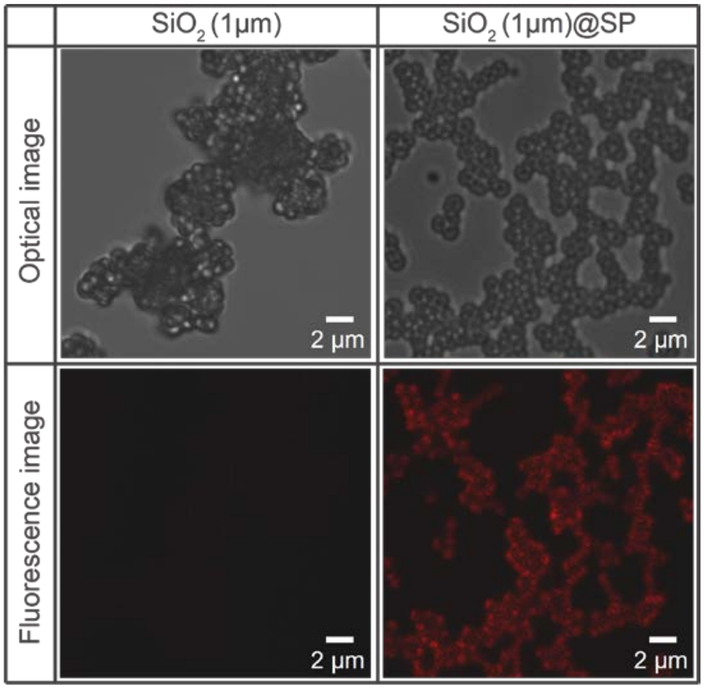

Figure S2. Optical and fluorescence images of bare SiO_2 particles (diameter = 1μm) and SP-functionalized SiO_2 (SiO_2@SP) after UV exposure. Only SP coated SiO_2 exhibited strong red fluorescence signal ($\lambda_{emission}$ ~ 620 nm) with a green excitation laser ($\lambda_{excitation}$ = 514 nm).

4. Surface-initiated polymerization and composite

4.1 Surface-initiated SET-LRP from SP functionalized SiO_2 particles

In a typical reaction, a Schlenk flask was charged with 50 mg of bromine-functionalized SiO_2 particle and 4 cm of copper(0) wire under N_2 atmosphere. 1 ml of DMSO was added to the flask and additional sonication was performed to disperse the SiO2. Then, 1 ml of methyl acrylate and 16 µL of Me_6TREN were injected, followed by three cycles of freeze-pump-thaw. After the flask was filled with N_2, the solution was stirred in a water bath for 2 h. The viscous solution was diluted with small amount of THF and precipitated in cold MeOH to yield polymer-grafted SiO2 particles. The polymer composites were further dried in a vacuum oven at 80 °C overnight to remove residual solvents. Control samples were prepared in the same manner.

4.1.1 Surface-initiated polymerization from micron-sized particles

Following the general method described in Section 4.1, PMA was successfully grown from the surface of SiO_2, confirmed by SEM (**Figure S3**).

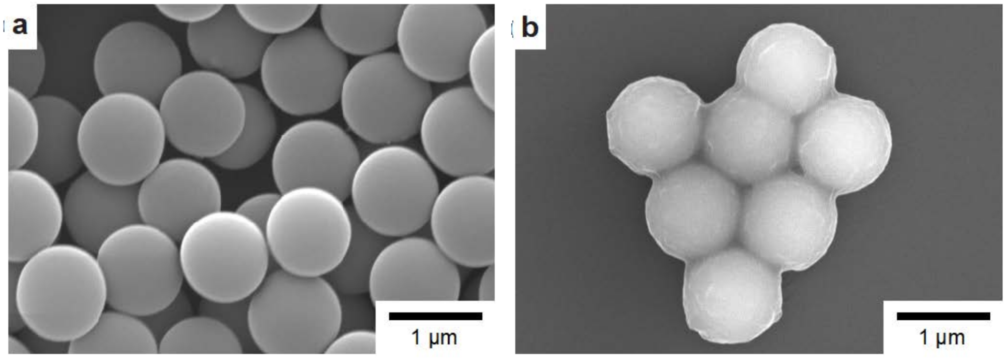

Figure S3. SEM images of SiO_2 microspheres. a, Bare SiO_2 particles (diameter = 1µm). **b,** Surface initiated polymerized PMA from SiO_2.

4.1.2 TEM images of bare SiO_2 nanoparticle

For comparison to the polymer-grown particles, TEM images of bare SiO_2 particles are included (**Figure S4**).

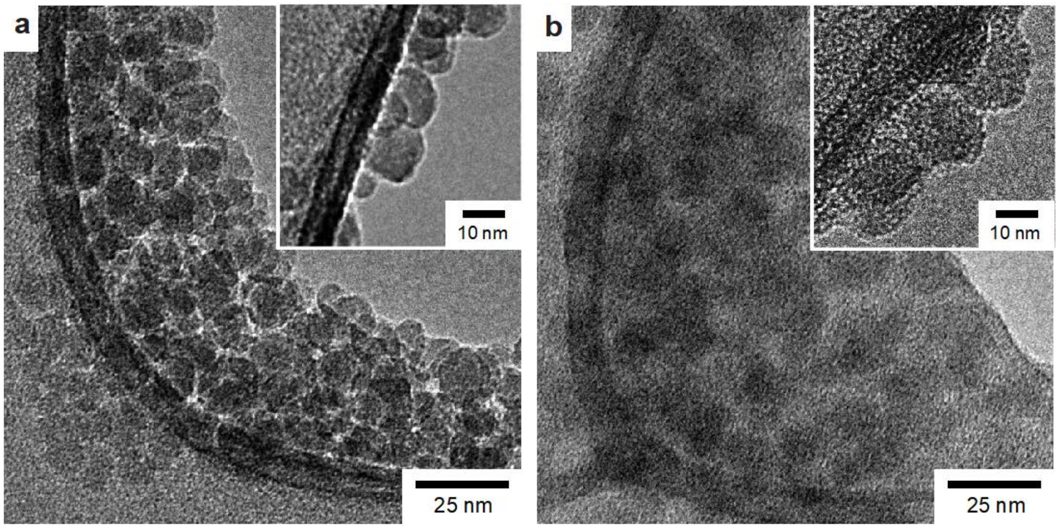

Figure S4. TEM images of SiO_2 nanoparticles. a, Bare SiO_2 nanoparticles (diameter = 10–15nm). **b,** PMA grown particles. Inset: Enlarged view of the SiO_2 nanoparticles. In contrast to PMA-grown SiO_2 nanoparticles, their surface was clean, and no polymer residue was observed.

4.2 Calculation of grafting density

Following the method described by Li et al.,[4] the grafting density of PMA on the SiO_2 particles was characterized. The weight percent of grafted PMA (*x*) and SiO_2 (*y*) were determined using thermogravimetric analysis (**Figure S5a, b**). Also, the molecular weight and dispersity of grafted PMA was characterized by GPC after removing SiO_2 with HF treatment (**Figure S5c**).

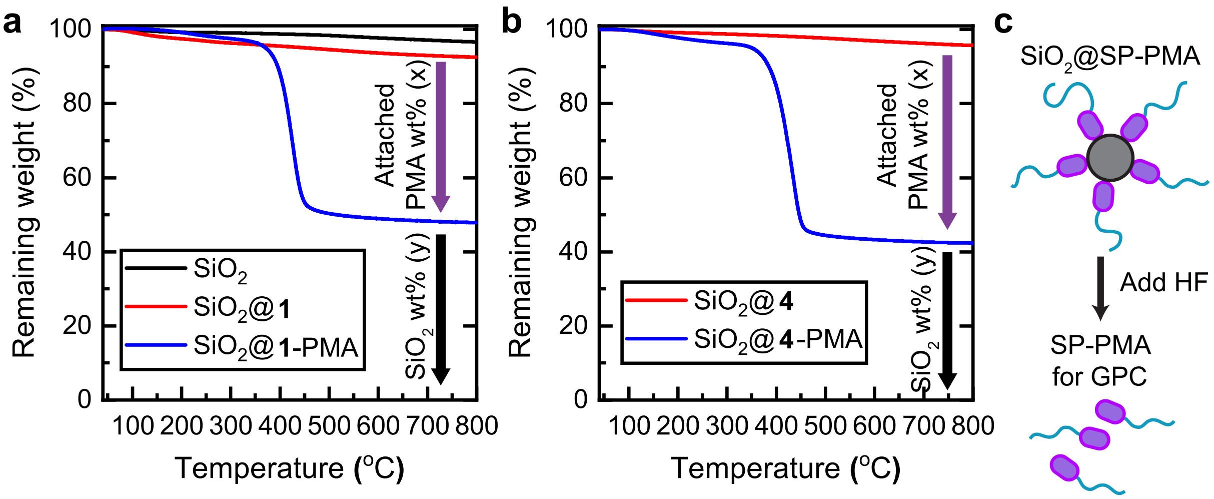

Figure S5. Method to determine PMA grafting density on functionalized SiO_2 particles. a, b TGA traces of bare SiO_2 (10–15nm), SiO_2 functionalized with active or control SP (SiO_2@**1**, SiO_2@**4**), and surface-initiated PMA from SiO_2 (SiO_2@**1**-PMA, SiO_2@**4**-PMA). **c,** Schematics of obtaining free polymers from the PMA grafted SiO_2 nanoparticles. The molecular weight and dispersity index of the free polymers was acquired using GPC.

Assuming the SiO_2 nanoparticle is a sphere with average radius (*r*) of 6.25×10^{-9} m and density (d) is 2.07×10^3 kg/m^3, the number of SiO_2 particles (N_{SiO_2}) is represented,

$$N_{SiO_2} = \frac{\text{Total weight of silica}}{\text{Weight of single silica}} = \frac{y}{\frac{4}{3}\pi r^3 d}$$

The total surface area of SiO_2 particles (*S*) is,

$$S = N_{SiO_2} \times 4\pi r^2$$

The number of polymer chains (N_{PMA}) is,

$$N_{PMA} = \frac{\text{Weight of total polymers}}{\text{Weight of single polymer}} = \frac{x}{M_n / N_A}$$

where M_n is number-averaged molecular weight and N_a is the Avogadro constant.

Finally, the grafting density (GD) is calculated by dividing the total surface area of SiO_2 (S) with the total number of polymer chains (N_{PMA}),

$$GD = \frac{S}{N_{PMA}}$$

The samples used for the solution activation and composite fabrication have a grafting density of 0.011 chains/nm^2. The samples tested for the DSC measurement have a slightly deviated grafting density depending on the molecular weights.

Table S1. Grafting density of PMA (GD) when different molecular weight of PMAs is attached

M_n (g mol^{-1})	GD (chains/nm^2)
64000	0.04
82000	0.02
215000	0.04
277000	0.01

4.3 Characterization of glass transition temperature (T_g) of PMA

When attractive interactions are present at a polymer–substrate interface, the confinement effects can increase the T_g of the polymer.[5,6] The ester group from PMA is believed to form hydrogen bonds with hydroxyl groups on the SiO_2,[7,8] so we assume that the T_g of the attached PMA on the SiO_2 is higher than that of free PMA if both of them have the same molecular weight. We measured the T_g of attached PMA to the SiO_2 and free PMA obtained by removing SiO_2 with HF by varying the molecular weight of PMA (**Figure S6Error! Reference source not found.**). At lower molecular weights, the attached polymers on the SiO_2 have a higher T_g than free polymers, while this difference is reduced as the molecular weight increases. This difference might be related with the different segmental mobility of polymer depending on the distance from the attractive substrates.[9] Since low molecular weight PMA can form a thinner layer on the SiO_2, the chain mobility is greatly reduced by the attractive interaction from the surface. This effect is minimized when the PMA forms a thicker layer. The T_g dependence on molecular weight was fitted to the Flory-Fox equation.

$$T_g = T_{g,\infty} - \frac{K}{M_n} \quad (1)$$

where $T_{g\infty}$ is the maximum T_g at a theoretical infinite molecular weight and K is polymer specific constant related with the chain-end free volume. In both cases, $T_{g\infty}$ is in the similar range of 20 °C, while K value decreases in half for attached PMA indicating the reduced free volume by attractive interaction with SiO_2.

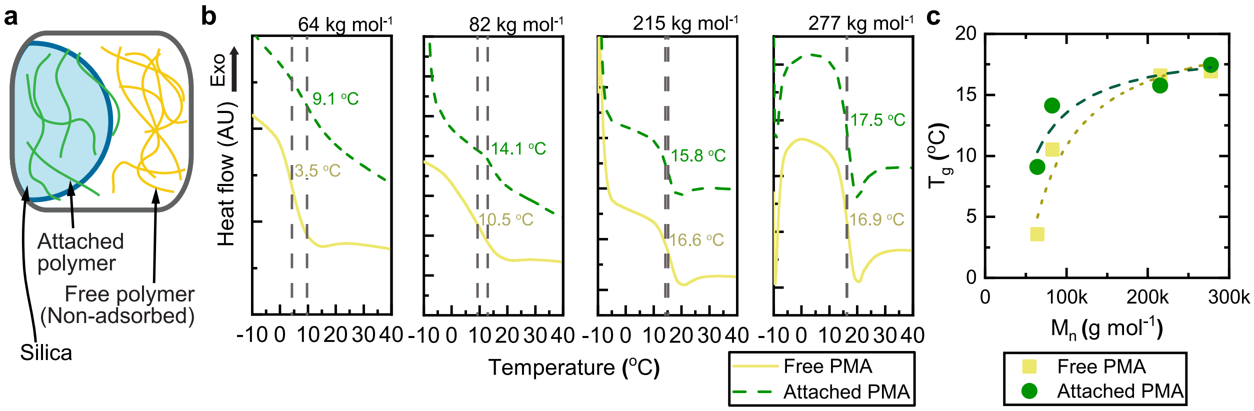

Figure S6. Glass transition temperature (T_g) of free and attached PMA. a, Attached polymers on the SiO_2 (green color) have different thermomechanical properties compared to free polymers due to attractive interaction between SiO_2 and PMA. **b,** Second DSC heating curves for free PMA (yellow green, solid lines) and attached PMA on SiO_2 (green, dash lines) with different molecular weights. The T_g is marked by black dashed lines. **c,** T_g of free PMA (red square) and attached PMA (blue circle). Dotted lines are fitted curves to the Flory-Fox equation.

4.4 Solution activation

Active (SiO_2@**1**-PMA) or control (SiO_2@**4**-PMA) samples with the attached PMA having molecular weight of 277 kg mol^{-1} (25 mg) was dispersed in 10 ml of methyl ether ketone by gentle bath sonication. The solution was transferred to the Suslick cell submerged in a cooling bath to achieve a consistent internal temperature of 3–5 °C of the reaction mixture throughout the experiments. Before sonication, a 2.0 ml of aliquot was removed and the UV spectrum was acquired (Pristine). The aliquot was then returned to the cell. Then, the solution was exposed to pulsed ultrasound (1.0 s on, 1.0 s off, 20 kHz) over 40 minutes. In every 10 minute, an aliquot was removed and UV spectrum was acquired.

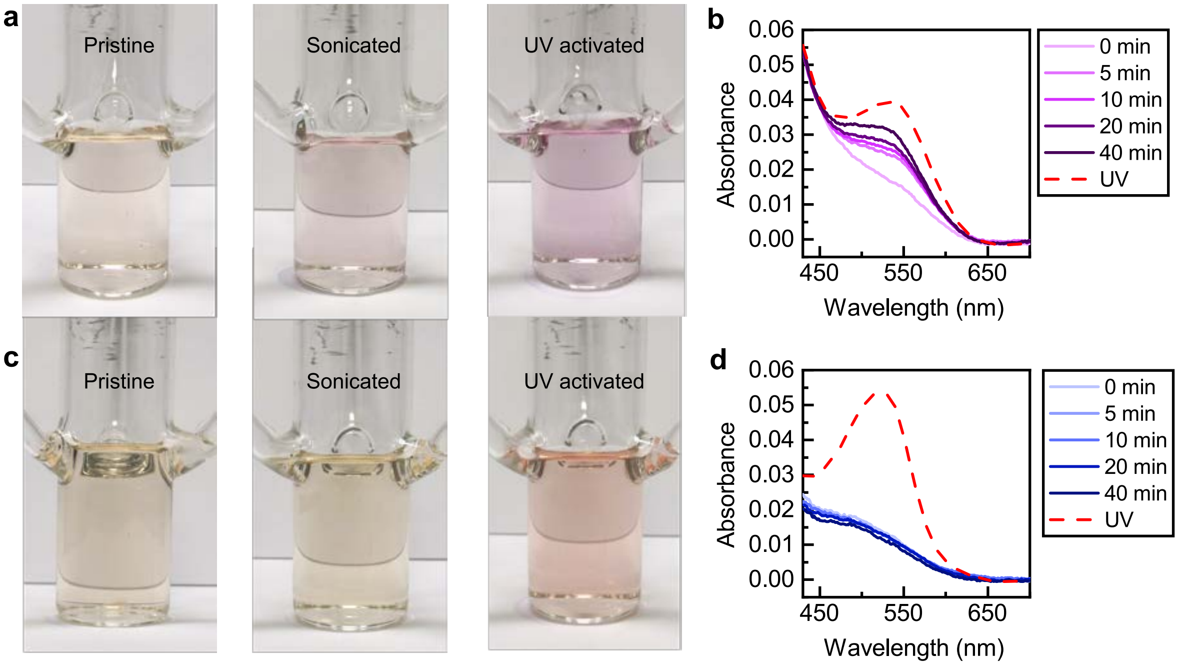

Figure S7. The change in color for active and control samples under sonication. a, Optical images of active (SiO_2@**1**-PMA) samples in the pristine, after sonication (40 min), and after UV activation. **b,** UV–vis spectra of sonicated active samples. **c,** Optical images of control (SiO_2@**4**-PMA) samples in the pristine, after sonication (40 min), and after UV activation. **d,** UV–vis spectra of sonicated control samples. Only active samples exhibit color change after sonication. However, both solutions exhibit spectrum change after UV exposure (red dashed lines).

4.5 Composites from linear PMA and SP functionalized SiO_2 nanoparticles

4.5.1 Fabrication method

Pristine PMA synthesized by SET-LRP from ethyl α-bromoisobutyrate initiator (M_n = 218 kg mol^{-1}, Đ = 1.15) was dissolved in methyl ether ketone. SP functionalized SiO_2 particles (SiO_2@**1**-PMA, SiO_2@**4**-PMA, and SiO_2@**6**) were dispersed in methyl ether ketone in separate vials. Then, the PMA solution and the dispersion of the functionalized SiO_2 particles were combined and stirred for at least 18 h. The weight fraction of pure SiO_2 in the composite solution was set to 8 wt%. The final dispersion was poured on a Teflon sheet and dried in a vacuum oven at 80 °C overnight. The dried polymer composite was molded into tensile dog-bone samples (gauge length: 5 mm, width: 2 mm, thickness: 0.5 mm) via compression molding. Three types of specimens were prepared: **A-L-Int** (the PMA composites containing SiO_2@**1**-PMA), **C-L-I** (the PMA composites containing SiO_2@**4**-PMA), and **C-L-II** (the PMA composites containing SiO_2@**6**).

4.5.2 Tension test

Dog-bone specimens were tested was uniaxially deformed at a strain rate of 0.1 s^{-1} by two opposing actuators while capturing optical images. Load was recorded using a 50-lb capacity load cell (Honeywell Sensotec) attached to one of the actuators. The acquired load-displacement data were converted to engineering stress and strain. Fluorescence intensity was calculated by averaging the red channel intensity of the gauge section of the sample. The stress–deformation ratio curve for each sample and the optical images of tensile samples are summarized in **Figure S8**.

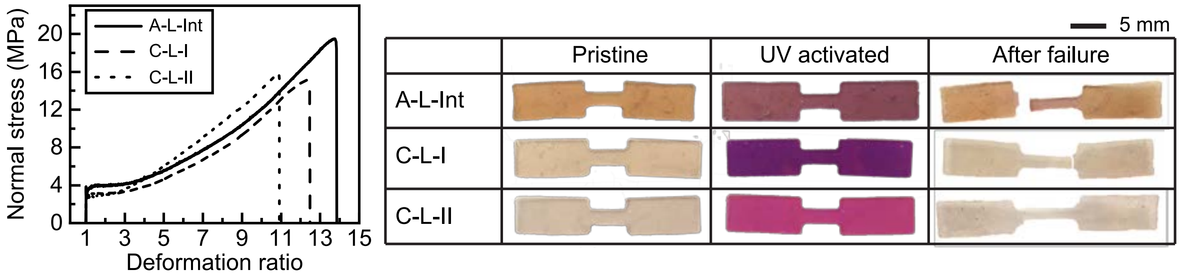

Figure S8. Tension test of PMA composites containing active (A-L-Int) or control particles (C-L-I and C-L-II). Stress–deformation ratio response (left) and digital images of tensile specimens (right).

Only active sample exhibited color change in the gauge area under tension (**Figure S9**).

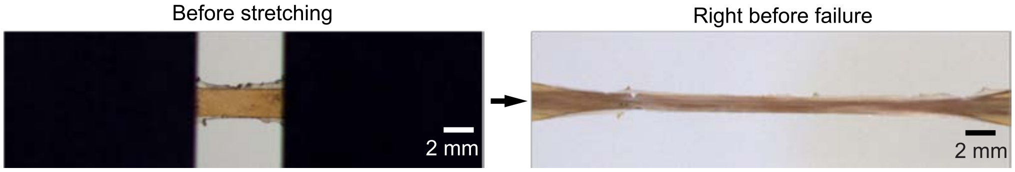

Figure S9. Tension test of PMA composites containing active particles. Dog-bone shaped specimen change color in the gauge area while stretching.

5. Mechanophore-linked cross-linked PMA

5.1 Cross-linked PMA (xPMA) with SP-functionalized SiO_2 particles

5.1.1 Polymerization procedure

The cross-linked reaction was conducted in a pre-made glass mold made from sandwiching silicone between two glass sheets. The glass mold was thoroughly flushed with N_2 to remove any oxygen in the environment. Typically, benzoyl peroxide (16.96 mg, 0.07 mmol, 1 eq), methacrylate functionalized SiO_2 particles (30 mg) were combined in a sealed vial and several cycles of vacuum purging and backfilling with N_2 were conducted. Then, methyl acrylate (2 ml, 22.21 mmol, 336 eq) was added to the vial and the solution was sonicated until it became a well-dispersed solution. Ethylene glycol dimethacrylate (35.49 µL, 0.19 mmol, 2.69 eq) and N,N-dimethylaniline (4 µL, 0.032 mmol, 0.45 eq) were added to the vial and sonicated. The final mixture was injected the glass molds while flushing with N_2 in a zip-lock bag. The sealed bag containing the glass mold kept in the freezer. After at least 12 h, the samples were removed from the mold and washed with methanol to remove residual monomers. The final films were dried in a vacuum oven. For manual testing, the xPMA sheets were cut into 5 mm × 30 mm strips. For tensile test, the film was laser cut into dog-bone shaped specimens (gauge length: 5 mm, width: 2 mm, thickness: 0.7 mm). Control samples were prepared in the same manner.

5.1.2 Tensile activation test: Optical and fluorescence images

Dog-bone specimens were tested with a combined mechanical and optical testing setup.[10] A 532-nm laser (0.6 mW) was used for the excitation light source for fluorescence measurements. The specimen was uniaxially deformed at a strain rate of 0.1 s^{-1} by two opposing actuators. Load was recorded using a 50-lb capacity load cell (Honeywell Sensotec) attached to one of the actuators. Excitation light was excluded by a long-pass emission filter (580 nm cutoff) and fluorescence images were captured with a color CCD (AVT Stingray F504c) on every second. The acquired load–displacement data were converted to engineering stress and strain. Fluorescence intensity was calculated by averaging the red channel intensity of the gauge section of the sample.Both optical images and fluorescence images were obtained while stretching. Formation of merocyanine under stress changed color from yellow to purple and emitted red fluorescence signals.

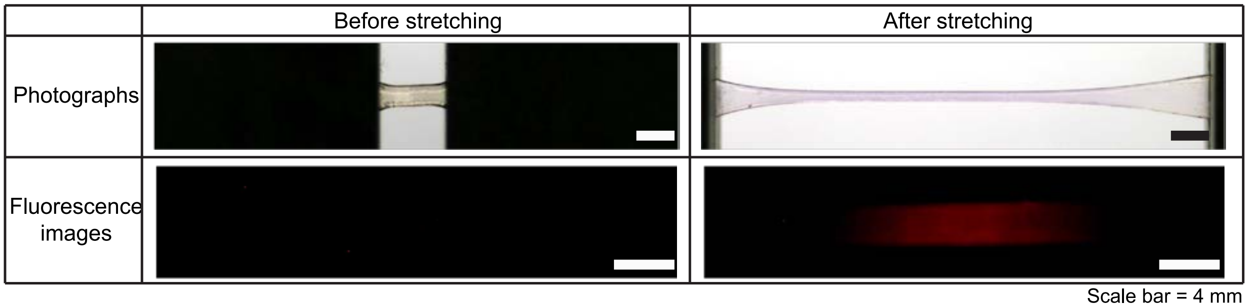

Figure S10. Tension test of xPMA composites containing active particles (A-Int). Dog-bone shaped specimen change color and emit fluorescence in the gauge area under tension.

5.1.3 Effect of the amount of SiO_2 on the mechanical properties

For composites with higher loading of SiO_2 (4.5wt%), both elastic modulus and tensile strength increased. However, there was no improvement on maximum deformation ratios compared to the composites with lower loading of SiO_2 (1.5wt%).

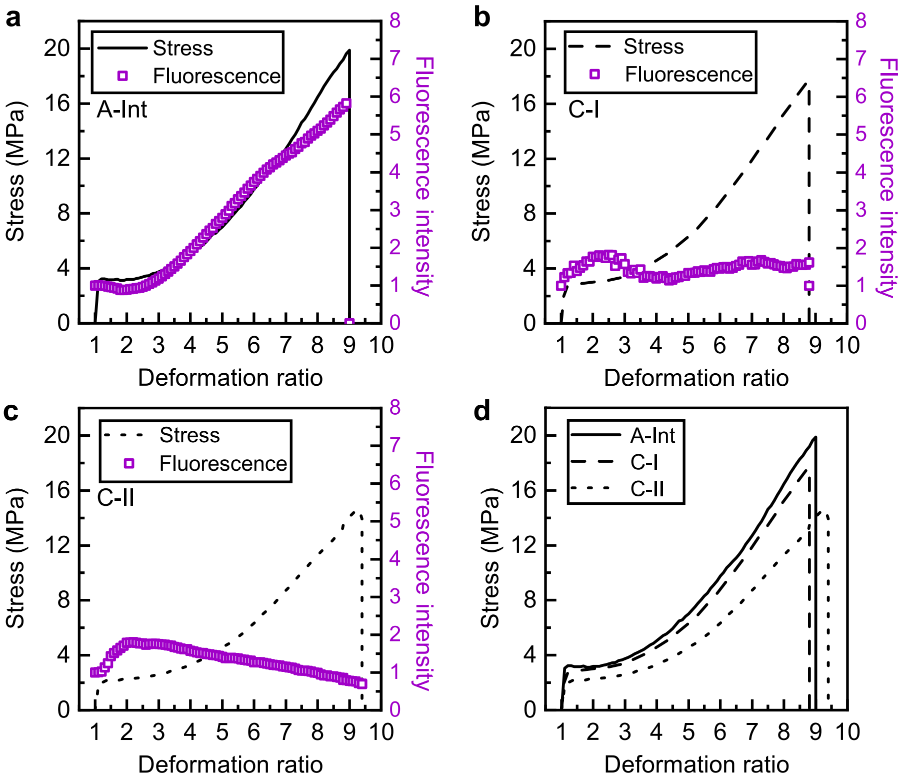

Figure S11. Combined stress and fluorescence data as a function of deformation ratio for xPMA specimen containing SP-functionalized SiO_2 (4.5 wt%) and 0.8 mol% of EGDMA. **a,** A-Int. **b,** C-I. **c,** C-II. Change in fluorescence signal is only observed from the "A-Int" sample. **d,** Comparison of stress–deformation ratio curves for each specimen. The "A-Int" sample exhibit enhanced mechanical properties compared to other control samples.

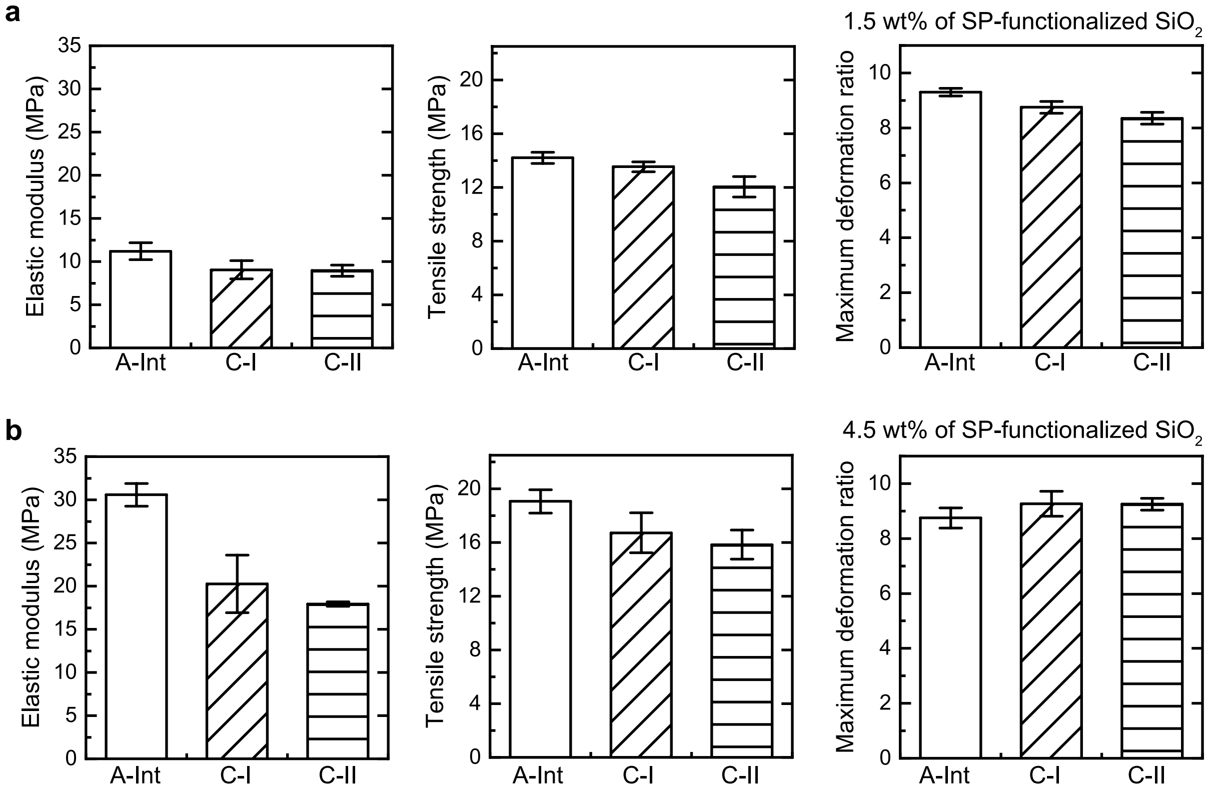

Figure S12. Comparison of characteristic mechanical properties of xPMA composites. Elastic modulus, tensile strength, and maximum deformation ratio were compared for **A-Int**, **C-I**, and **C-II** when **a**, 1.5 wt% of SP-functionalized SiO_2 was added or **b**, 4.5 wt% of SP-functionalized SiO_2 was added.

The strain energy was calculated from the area under the stress–strain curve for each sample (**Figure S13**). The **A-Int** samples have higher strain energy than two control samples, regardless of the amount of SiO_2.

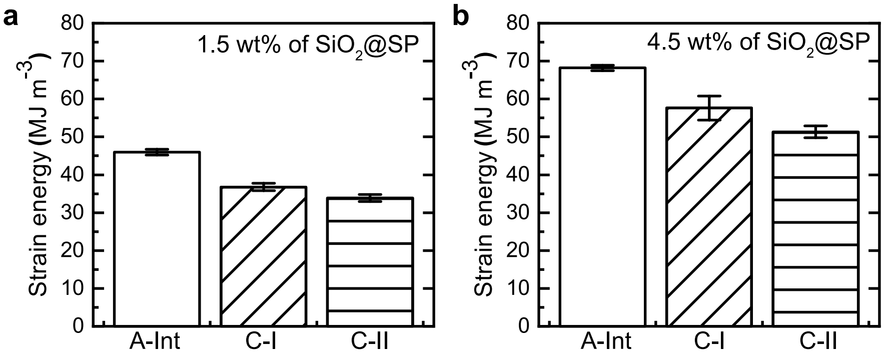

Figure S13. Strain energy of the xPMA composites. a, Composites containing 1.5 wt% of SP functionalized SiO_2. **b**, Composites containing 4.5 wt% of SP functionalized SiO_2.

5.2 Comparison of the mechanical reactivity of **A-Int** to that of **A-Bulk**.

5.2.1 Characterization of attached SP to SiO_2 particles

The attached amount of SP to SiO_2 was determined by TGA, which was around 2.5 wt% (**Figure S14**).

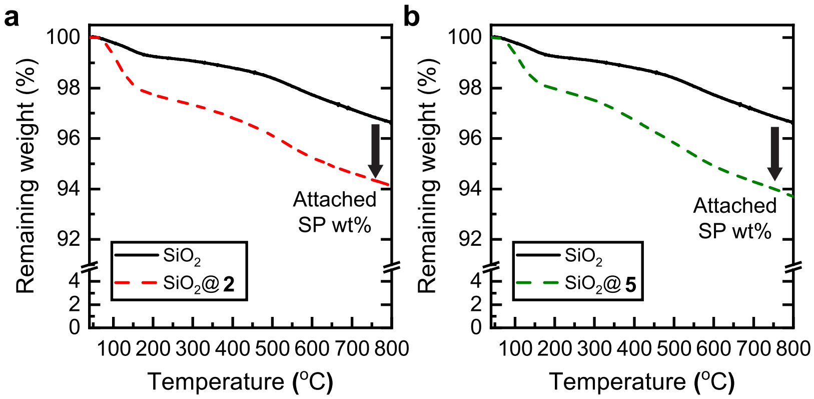

Figure S14. Determination of attached SP amount on SiO_2 particles. a, TGA traces of bare SiO_2 and SiO_2@**2** and **b**, TGA traces of bare SiO_2 and SiO_2@**5**

5.2.2 Polymerization procedure for bulk specimens

The glass mold was prepared from the same method described in 5.1.1. Typically, the cross-linked PMA (0.5 mol%) containing SP at the interfaces in the composite was prepared by following methods. Benzoyl peroxide (16.96 mg, 0.07 mmol, 1 eq), methacrylate functionalized SiO_2 particles (SiO_2@**2**, 30 mg, 1.3×10^{-3} mmol of **2** attached) were combined in a sealed vial and several cycles of vacuum purging and backfilling with N_2 were conducted. Then, methyl acrylate (2 ml, 22.21 mmol, 336 eq) was added to the vial and the solution was sonicated until it became a well-dispersed solution. Ethylene glycol dimethacrylate (22 µL, 0.12 mmol, 1.68 eq) and N,N-dimethylaniline (4 µL, 0.032 mmol, 0.45 eq) were added to the vial and sonicated. The final mixture was injected to the glass molds while flushing with N_2 in a zip-lock bag. The sealed bag containing the glass mold kept in the freezer.

The cross-linked PMA (0.5 mol%) containing SP at the PMA matrix was fabricated using the similar method. Benzoyl peroxide (16.96 mg, 0.07 mmol, 1 eq), methacrylate functionalized SiO_2 particles (SiO_2@**5**, 30 mg), and **3** (1 mg, 1.3×10^{-3} mmol, the same amount of active SP from the interfacial case) were combined in a sealed vial and several cycles of vacuum purging and backfilling with N_2 were conducted. Then, methyl acrylate (2 ml, 22.21 mmol, 336 eq) was added to the vial and the solution was sonicated until it became a well-dispersed solution. Ethylene glycol dimethacrylate (21 µL, 0.11 mmol, 1.68 eq) and N,N-dimethylaniline (4 µL, 0.032 mmol, 0.45 eq)

were added to the vial and sonicated. The final mixture was injected the glass molds while flushing with N_2 in a zip-lock bag. After at least 12 h, the samples were removed from the mold and washed with methanol to remove residual monomers. The final films were dried in a vacuum oven and laser cut into dog-bone shaped specimens.

5.2.3 Mechanical properties of A-Int and A-Bulk specimens with increasing concentrations of crosslinker

Four or five of each specimen type were tested and the stress–deformation ratios were recorded (**Figure S15**).

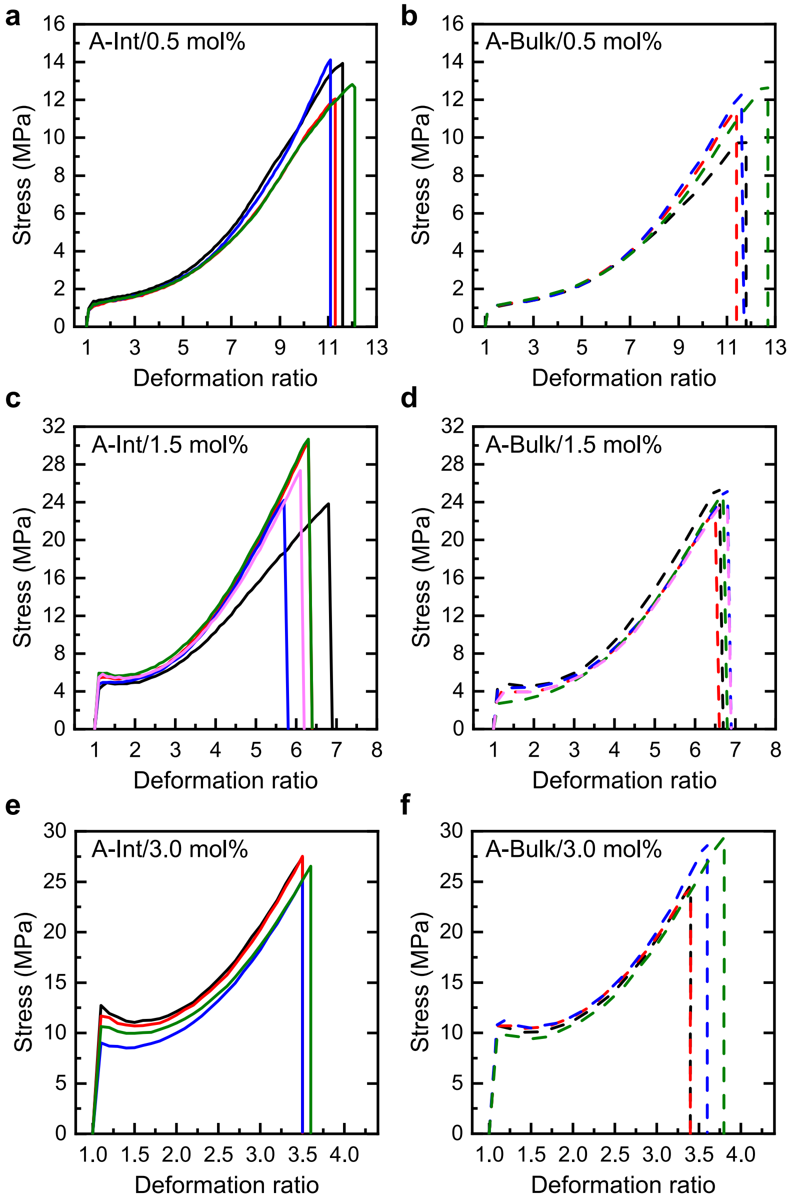

Figure S15. Stress–deformation ratio curves for xPMA composites. Both A-Int and A-Bulk kept similar mechanical properties.

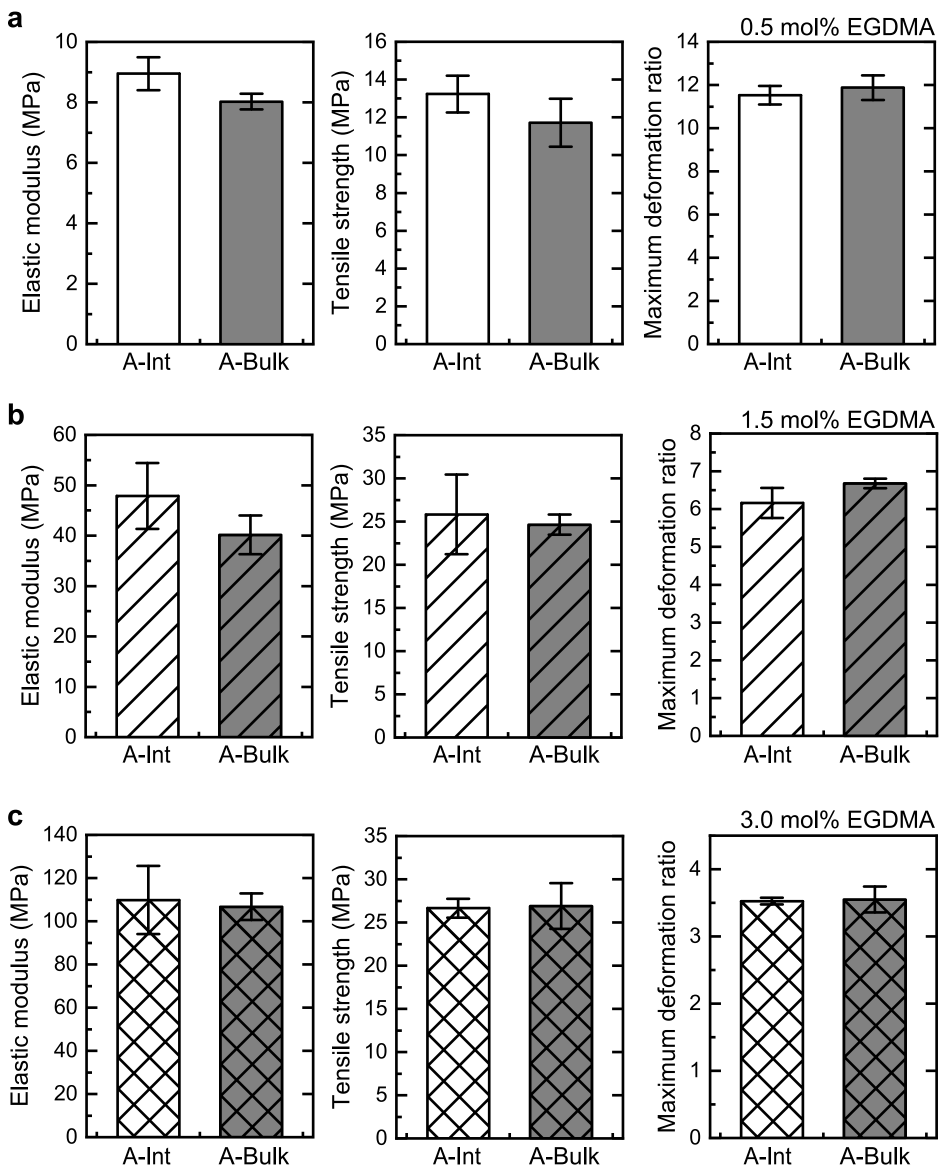

Figure S16. Comparison of characteristic mechanical properties of xPMA incorporating A-Int and A-Bulk. Elastic modulus, tensile strength, and maximum deformation ratio were compared when the overall cross-linking density is **a**, 0.5 mol%, **b**, 1.0 mol%, and **c**, 3.0 mol%. A-Int and A-Bulk have the similar mechanical properties.

5.2.4 Activation stress and activation true stress

Corresponding stress value at the onset deformation ratio (Λ) was defined as the activation stress (σ_{act}). By assuming an incompressible rubber (Poisson's ratio = 0.5), the activation true stress ($\sigma_{true,act}$)was calculated by the following equation:

$$\sigma_{true,act} = \sigma_{act} \times \Lambda \tag{2}$$

Table S2. Corresponding activation stress and true stress values at different cross-linking density

	σ_{act} (MPa)			$\sigma_{true,act}$ (MPa)	
	**A-Int**	**A-Bulk**		**A-Int**	**A-Bulk**
0.5 mol%	1.3 ± 0.1	2.0 ± 0.1		2.0 ± 0.2	9.1 ± 0.6
1.5 mol%	5.2 ± 0.6	5.2 ± 1.0		7.4 ± 0.8	15 ± 3
3.0 mol%	11 ± 2	11 ± 0.6		12 ± 2	20 ± 1

6. Finite Elements Smulations

Finite Elements (FE) simulations were performed using ABAQUS/CAE 6.13.

A generic area was selected from TEM images of the cross-section of the xPMA/SiO_2 composite and then modeled as shown in **Figure 4a** of the manuscript. In particular, the analyzed area contains eight silica particles with a diameter of 18 nm, embedded into a PMA matrix, and located at different distances between each other.

The PMA/SiO_2 composite was modeled as a 2D deformable body. While SiO_2 was considered a simple elastic material (Young's modulus= 73 GPa, and Poisson's ratio= 0.15), hyperelastic properties were assigned to the PMA matrix. Specifically, the Arruda-Boyce hyperelastic model was used to describe the strain energy potential, and the model coefficients were obtained by fitting experimental test data of PMA samples.[11]

A linear displacement was applied to the two free edges along the y direction (see **Figure 4**) until reaching a final deformation ratio of 3.5. The 2D model was discretized by CPS8R elements, and mesh refinement studies were performed in order to validate the accuracy of the model.

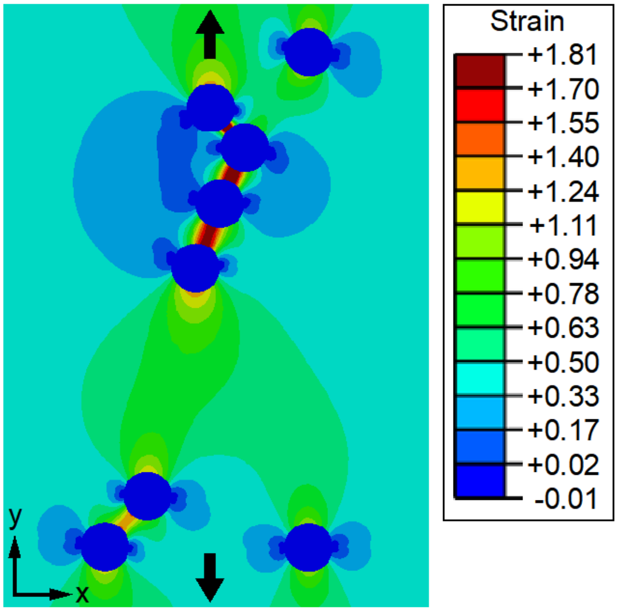

Figure S17. Normalized strain profile for xPMA/SiO2 composites under tension.

7. References

[1] D. A. Davis, A. Hamilton, J. Yang, L. D. Cremar, D. Van Gough, S. L. Potisek, M. T. Ong, P. V. Braun, T. J. Martínez, S. R. White, J. S. Moore, N. R. Sottos, T. J. Martinez, S. R. White, J. S. Moore, N. R. Sottos, *Nature* **2009**, *459*, 68.

[2] G. R. Gossweiler, G. B. Hewage, G. Soriano, Q. Wang, G. W. Welshofer, X. Zhao, S. L. Craig, *ACS Macro Lett.* **2014**, *3*, 216.

[3] T. Stafforst, D. Hilvert, *Chem. Commun.* **2009**, 287.

[4] J. Li, T. Shiraki, B. Hu, R. A. E. Wright, B. Zhao, J. S. Moore, *J. Am. Chem. Soc.* **2014**, *136*, 15925.

[5] P. Rittigstein, J. M. Torkelson, *J. Polym. Sci. Part B-Polymer Phys.* **2006**, *44*, 2935.

[6] P. Rittigstein, R. D. Priestley, L. J. Broadbelt, J. M. Torkelson, *Nat. Mater.* **2007**, *6*, 278.

[7] R. Y. Hong, H. P. Fu, Y. J. Zhang, L. Liu, J. Wang, H. Z. Li, Y. Zheng, *J. Appl. Polym. Sci.* **2007**, *105*, 2176.

[8] D. W. Janes, J. F. Moll, S. E. Harton, C. J. Durning, *Macromolecules* **2011**, *44*, 4920.

[9] D. S. Fryer, R. D. Peters, E. J. Kim, J. E. Tomaszewski, J. J. De Pablo, P. F. Nealey, C. C. White, W. L. Wu, *Macromolecules* **2001**, *34*, 5627.

[10] B. a. Beiermann, S. L. B. Kramer, P. a. May, J. S. Moore, S. R. White, N. R. Sottos, *Adv. Funct. Mater.* **2014**, *24*, 1529.

[11] E. M. Arruda, M. C. Boyce, *J. Mech. Phys. Solids* **1993**, *41*, 389.
